# Inhibition of human mevalonate kinase by allosteric inhibitors of farnesyl pyrophosphate synthase

**DOI:** 10.1002/2211-5463.13853

**Published:** 2024-06-23

**Authors:** Saman Salari, Hiu‐Fung Lee, Youla S. Tsantrizos, Jaeok Park

**Affiliations:** ^1^ Department of Biochemistry Memorial University of Newfoundland St. John's Canada; ^2^ Department of Chemistry McGill University Montreal Canada

**Keywords:** farnesyl pyrophosphate, feedback inhibition, isoprenoid synthesis, mevalonate kinase, mevalonate pathway, phosphonate compounds

## Abstract

Mevalonate kinase is a key regulator of the mevalonate pathway, subject to feedback inhibition by the downstream metabolite farnesyl pyrophosphate. In this study, we validated the hypothesis that monophosphonate compounds mimicking farnesyl pyrophosphate can inhibit mevalonate kinase. Exploring compounds originally synthesized as allosteric inhibitors of farnesyl pyrophosphate synthase, we discovered mevalonate kinase inhibitors with nanomolar activity. Kinetic characterization of the two most potent inhibitors demonstrated *K*_i_ values of 3.1 and 22 nm. Structural comparison suggested features of these inhibitors likely responsible for their potency. Our findings introduce the first class of nanomolar inhibitors of human mevalonate kinase, opening avenues for future research. These compounds might prove useful as molecular tools to study mevalonate pathway regulation and evaluate mevalonate kinase as a potential therapeutic target.

## Graphical abstract

Mevalonate kinase constitutes a key regulatory step in the mevalonate pathway, an established drug target for its role in the synthesis of isoprenoid molecules. Using a structure‐based rational approach, we identified several synthetic compounds that inhibit mevalonate kinase with nanomolar potency. Further analysis revealed their mode of inhibition and the structural features that correlate with their inhibitory potency.

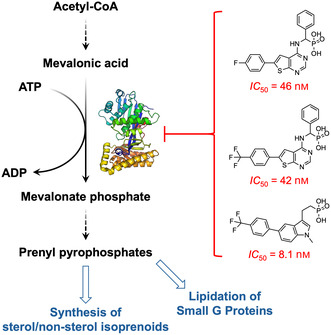

**Abbreviations**
FPP: farnesyl pyrophosphate
FPPS: farnesyl pyrophosphate synthase
MK: mevalonate kinase
MVA: mevalonic acid

Human mevalonate kinase (MK, EC2.7.1.36) is an 85 kDa homodimeric enzyme that catalyzes the phosphorylation of mevalonic acid (MVA) to mevalonate‐5‐phosphate [1]. It is a key regulatory enzyme of the mevalonate pathway [2], which produces the building blocks for a diverse class of molecules known as isoprenoids [3]. Isoprenoids play essential roles in various aspects of life, including gene expression, signal transduction, and electron transport. Consequently, dysregulation of the mevalonate pathway holds significant implications for human health and has been associated with hypercholesterolemia [4], cancer [5], Alzheimer's disease [6], muscular dystrophy [7], and other inflammatory and neurological disorders [8, 9, 10]. Notably, genetic deficiency in MK activity has been established as the cause of the latter diseases, such as hyperimmunoglobulinemia D and periodic fever syndrome [9].

In healthy cells, a complex web of feedback loops tightly regulates the mevalonate pathway at both transcriptional and post‐translational levels [11, 12]. For example, the transcription of MK [13], as well as that of other pathway enzymes, HMG‐CoA synthase [14], HMG‐CoA reductase [15], and farnesyl pyrophosphate (FPP) synthase [16, 17], is negatively regulated by the downstream metabolite cholesterol (Fig. 1). At the enzymatic level, pathway metabolites can directly bind to and inhibit upstream enzymes (Fig. 1). For example, FPP inhibits human MK by competing with ATP, the phosphoryl donor substrate of the enzyme [18, 19, 20]. FPP can also inhibit FPP synthase (FPPS), the enzyme immediately responsible for its production. In this case, it acts as an allosteric effector that locks the enzyme's conformation in an inactive state [21]. The multi‐layered feedback mechanisms highlight the critical importance of preventing uncontrolled isoprenoid synthesis, which can lead to cellular dysfunction and the development of diseases.

**Fig. 1:**
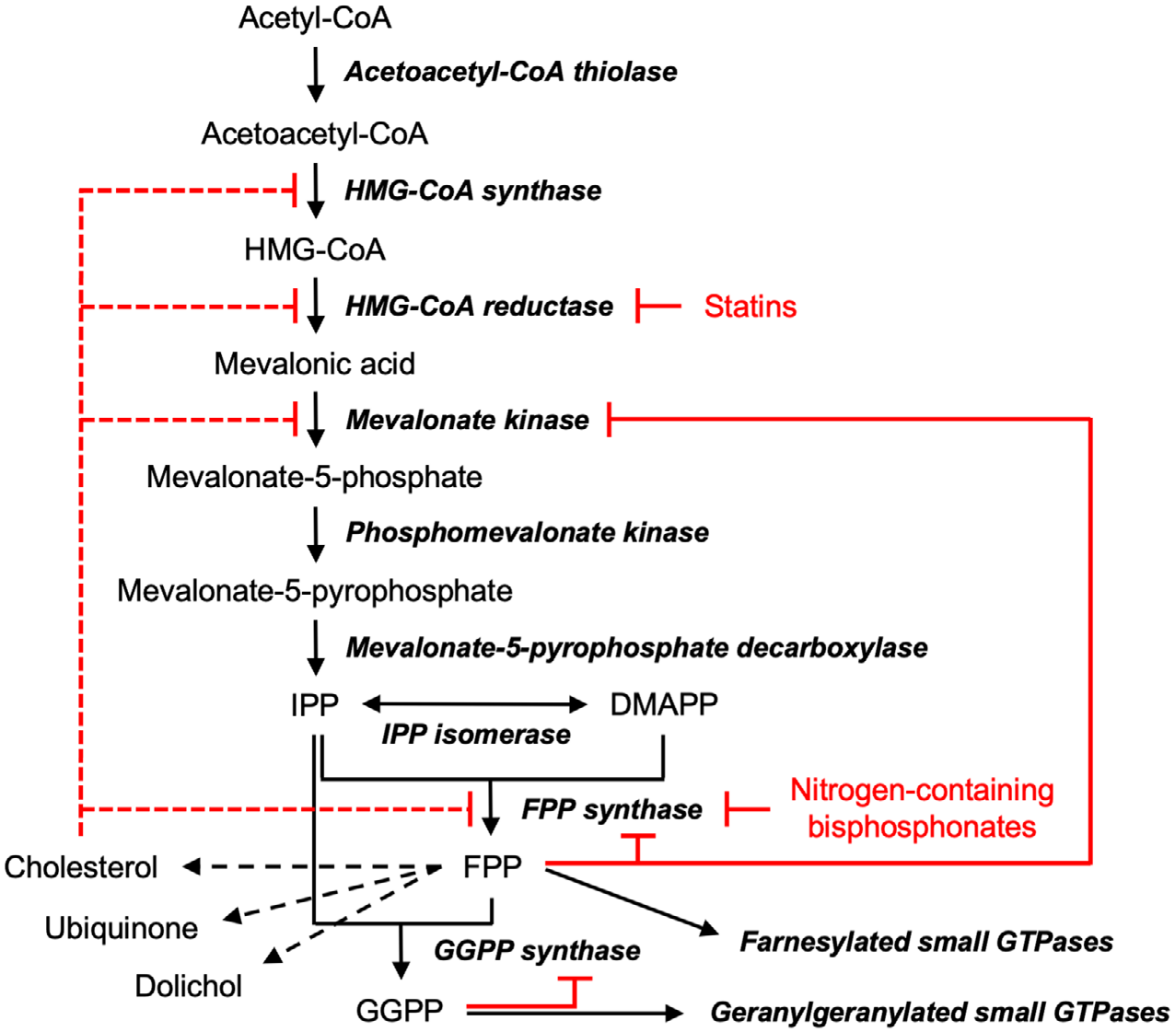
Mevalonate pathway and its regulation. The names of the pathway enzymes are given in bold italics. Dotted arrows indicate multi‐enzyme processes downstream of the mevalonate pathway. Dotted red lines represent the downregulation of pathway enzyme expression by cholesterol through sterol regulatory elements, while solid red lines indicate direct inhibition of pathway enzymes by downstream metabolites. Sites of pharmacological intervention by current clinical agents are also indicated. Abbreviations for isoprenyl pyrophosphates: DMAPP, dimethylallyl pyrophosphate; FPP, farnesyl pyrophosphate; GGPP, geranylgeranyl pyrophosphate; IPP, isopentenyl pyrophosphate.

The profound implications of the mevalonate pathway on cellular activities make it a prominent pharmacological target. Current clinical agents targeting this pathway include two blockbuster classes of drugs: statins, used to treat hypercholesterolemia [22], and nitrogen‐containing bisphosphonates, used to treat bone‐related disorders such as osteoporosis [23] (Fig. 1). Efforts to discover new drugs targeting the mevalonate pathway have focused on the anticancer benefits of the pathway inhibition. Elevated activity of this pathway has been observed in cancer cells, indicating their dependency on the continuous availability of MVA‐derived metabolites [5, 12]. Therefore, downregulation of the mevalonate pathway has emerged as an attractive approach to enhance cancer therapy [24]. Notably, allosteric inhibitors of FPPS have gained significant attention as potential lead molecules. Several different classes of compounds have been explored by multiple research groups [25, 26, 27, 28, 29, 30, 31]. Our investigations have identified monophosphonate‐containing compounds that closely mimic FPP in their inhibition mechanism [26, 30, 31]. While the clinical relevance of allosteric inhibitors of FPPS has yet to be reported, their discovery has presented an intriguing possibility. Given their mechanism of FPPS inhibition—by allostery through the FPP‐binding site—they may also inhibit MK, which shares FPP as a common feedback regulator.

In the present study, we investigate the hypothesis that monophosphonate inhibitors of FPPS can also inhibit MK. By screening representative compounds from our library of FPPS allosteric inhibitors, we identify those that inhibit MK with nanomolar potency. Kinetic studies reveal their competitive relationship with ATP and uncompetitive relationship with MVA. Further analysis highlights key features of these compounds that may contribute to their inhibitory potency and can be explored in future structure–activity relationship studies.

## Materials and methods

### Chemicals and reagents

Common buffers and salts were purchased from BioShop Canada. All other chemicals, including enzyme substrates, were purchased from MilliporeSigma unless specified otherwise. To note, MVA was purchased as mevalonolactone (DL‐; MilliporeSigma) and converted as previously described [32], by treatment with KOH and subsequent pH adjustment to 7.5.

### Expression and purification of human MK

**Fig. 2:**
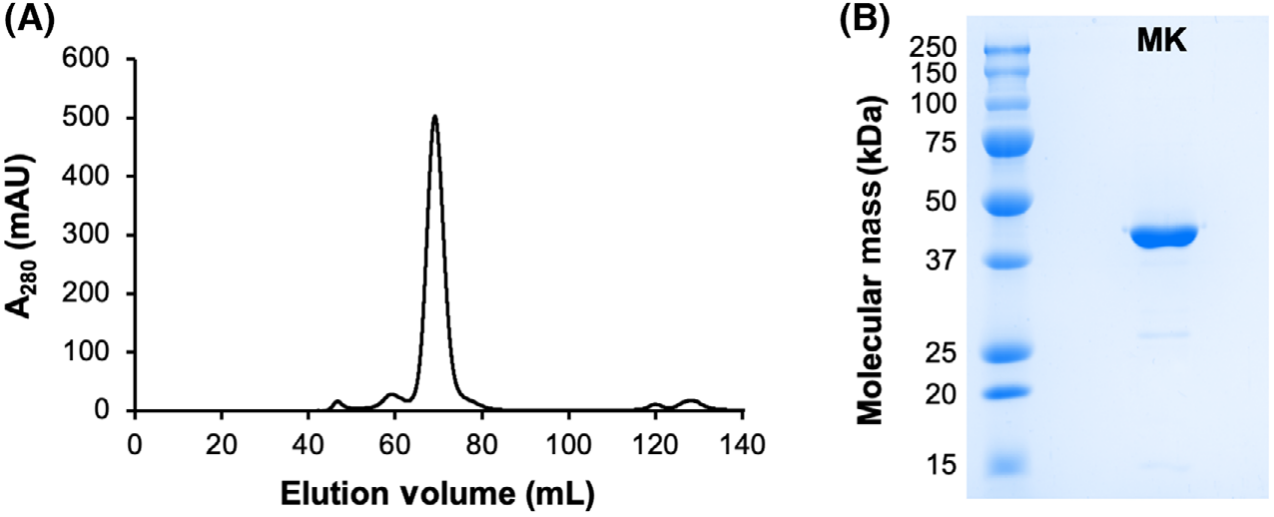
Purification of recombinant human MK. (A) Elution profile from size exclusion chromatography. Fractions with A_280_ > 300 mAU were pooled and stored for subsequent experiments. (B) SDS/PAGE analysis of purified MK. Sample purity was estimated by a BioRad ChemiDoc Imaging System.

A pET‐15b plasmid encoding human MK (UniProt Q03426) with an N‐terminal His_6_‐tag was transformed into *E. coli* BL21(DE3) cells (New England Biolabs, Whitby, ON, Canada). The cells were cultured in LB at 37 °C until the OD_600_ of the culture reached 0.6. Expression of the recombinant enzyme was induced by 0.1 mm IPTG for 6 h at 37 °C. The cells were lysed by sonication, and the lysate was subjected to Ni‐affinity chromatography (HisTrap HP, Cytiva, Vancouver, BC, Canada) followed by size exclusion chromatography (HiLoad Superdex 200 PG, Cytiva) (Fig. 2A). Subsequent analysis by SDS/PAGE revealed a sample purity of 97.2% (Fig. 2B). The purified enzyme was flash‐frozen in liquid nitrogen and stored at −80 °C. The final sample buffer (i.e., the size exclusion buffer) consisted of 10 mm HEPES (pH 7.5), 500 mm NaCl, 2 mm β‐mercaptoethanol, and 5% glycerol.

### Kinetic characterization of MK

MK activity was measured by using a coupled enzyme assay as previously described [20]. The reporter enzymes, pyruvate kinase and lactate dehydrogenase, were purchased as a pre‐mixed buffered solution from MilliporeSigma. Reactions proceeded in a buffer containing 50 mm HEPES (pH 7.5), 100 mm KCl, 10 mm MgCl_2_, 0.15 mm phosphoenolpyruvate, 0.15 mm NADH, ~ 8 units of pyruvate kinase, ~ 12 units of lactate dehydrogenase, variable concentrations of ATP and MVA, and 0.5 μg mL^−1^ of MK in a total volume of 1 mL. All reactions were carried out at 32 °C in triplicate unless otherwise noted. Each reaction was initiated by adding 10 μL of MK (50 μg mL^−1^; diluted from the −80 °C stock in 50 mm HEPES, pH 7.5) to 990 μL of the reaction buffer containing all the other components except MK (pre‐incubated at 32 °C for 5 min) and immediately transferring to a 1 mL disposable polystyrene cuvette (Fisherbrand) pre‐placed in the temperature‐controlled cell holder of an Agilent 8453 UV–visible Spectroscopy System. Blank reactions were added with 10 μL of 50 mm HEPES (pH 7.5) only. Reaction progress was continuously monitored by measuring the A_340_ for 1 min. To convert the measured absorbance to product concentration, an absorption coefficient of NADH determined from a calibration curve (5.4107 cm^−1^ mm ^−1^) was used. Initial rate experiments were conducted by varying the concentration of one substrate while keeping the other at a saturating concentration of 5 mm. Kinetic parameters were determined by fitting the rate data to the Michaelis–Menten model (Eqn 1),

$$v_{i}=\frac{V_{\max}\left[S\right]}{K_{m}+\left[S\right]},$$

where [*S*] represents the concentration of the varied substrate. Data analysis was performed with graphpad prism software (GraphPad Software, Boston, MA, USA).

### Synthesis of phosphonate compounds

The synthesis of compounds **1**–**8**, **15**, **17**, **18**, **20**, **22**, and **23** has been previously described [26, 30, 33, 34, 35, 36]. Their former compound codes and reference publications are annotated in Tables 1 and 2. The synthesis of compounds **9**–**14**, **16**, **19**, **21**, and **24** is detailed below. All compounds were fully characterized by ^1^H, ^13^C, and ^31^P NMR in D_2_O as the solvent, with the addition of 1–3 equivalents of NaOD to make the mono‐, di‐, or trisodium salt for better aqueous solubility (Supporting Information). MS and C18 reversed phase HPLC were also used to confirm the structure and acceptable purity of the compounds (Supporting Information).

**Table 1:** Chemical structures of the monophosphonate compounds tested in this study. For previously reported compounds, the former compound code and the reference publication are indicated in brackets.

Compound	Structure	Compound	Structure
**1** (**7**, ref. [30])	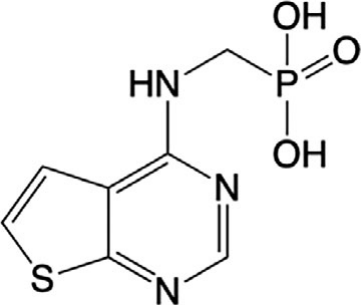	**2** (**24**, ref. [30])	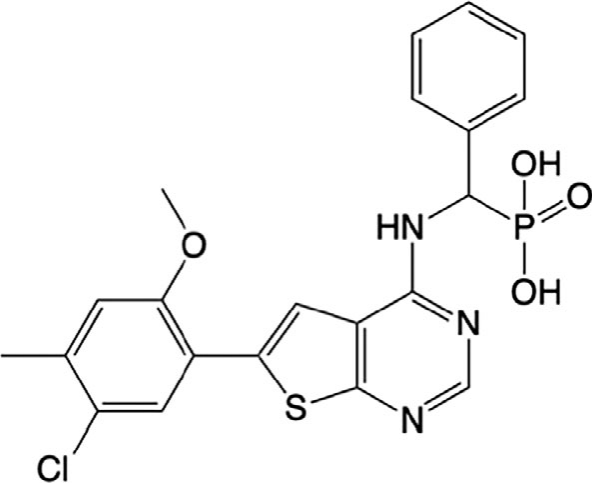
**3** (**36**, ref. [30])	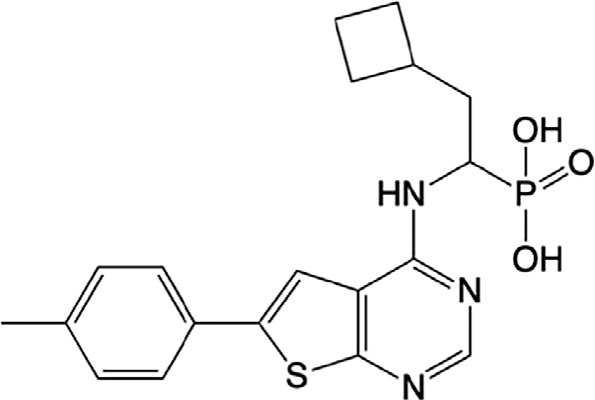	**4** (**37**, ref. [30])	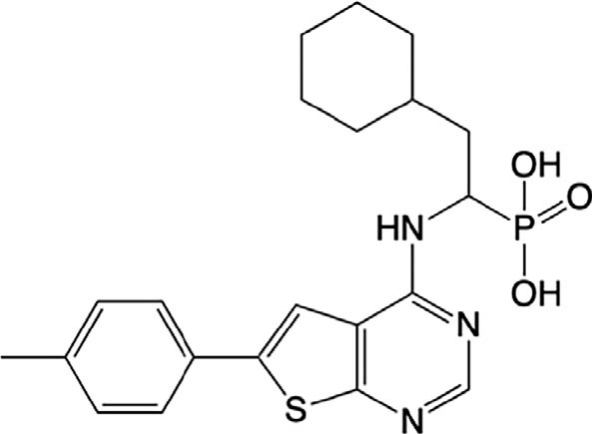
**5** (**38**, ref. [30])	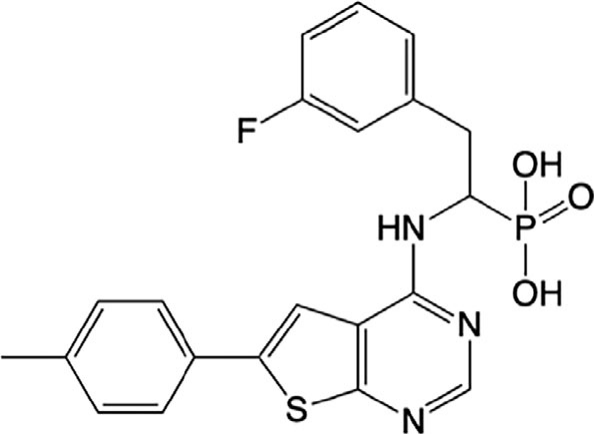	**6** (**20**, ref. [30])	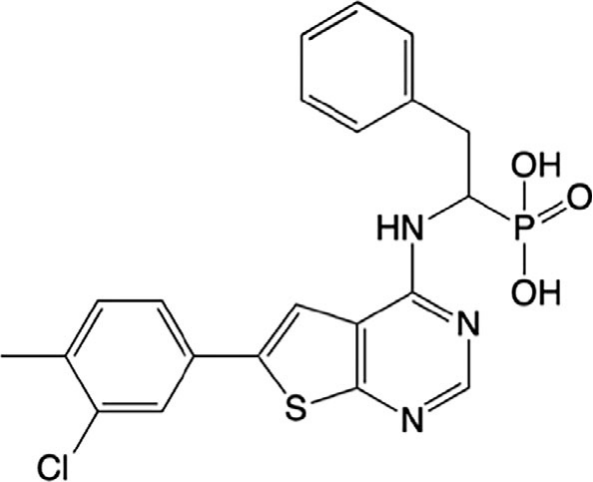
**7** (**22**, ref. [30])	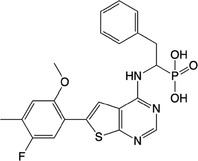	**8** (**21**, ref. [30])	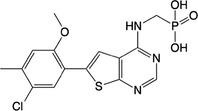
**9**	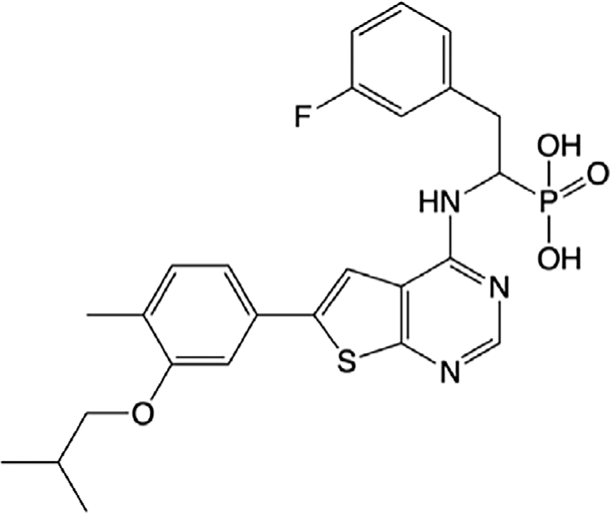	**10**	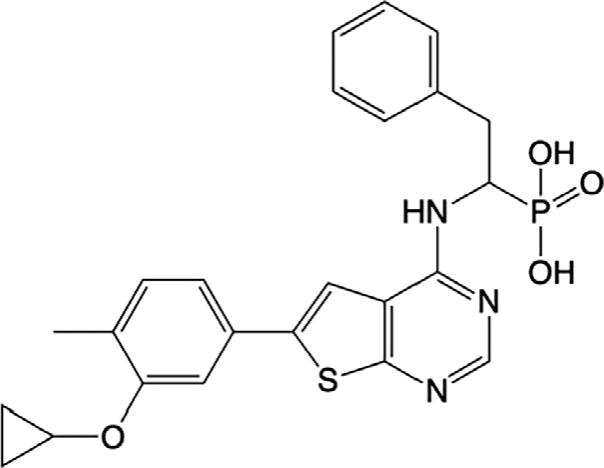
**11**	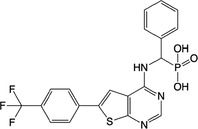	**12**	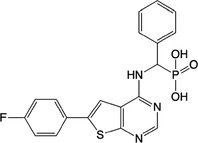
**13**	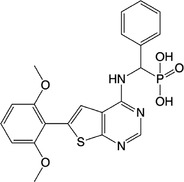	**14**	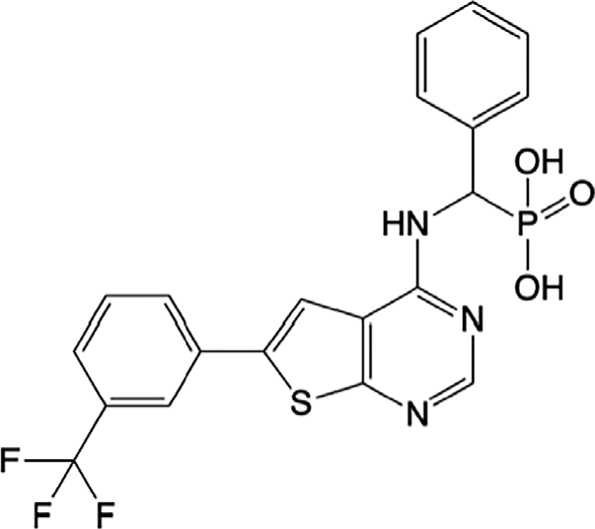
**15** (**23**, ref. [30])	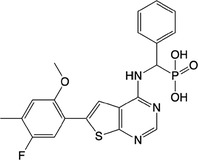	**16**	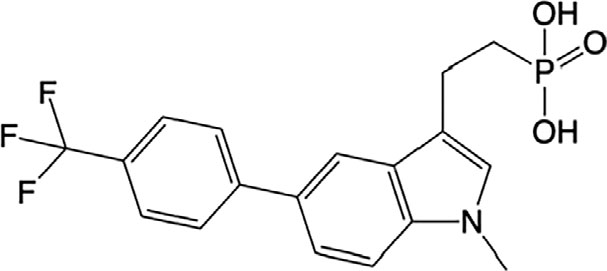

**Table 2:** Chemical structures of the bisphosphonate compounds tested in this study. For previously reported compounds, the former compound code and the reference publication are indicated in brackets.

Compound	Structure	Compound	Structure
**17** (**6i**, ref. [36])	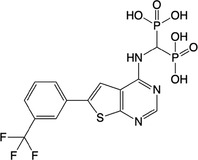	**18** (**6 g**, ref. [36])	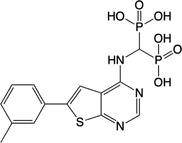
**19** (**11 m**, ref. [33])	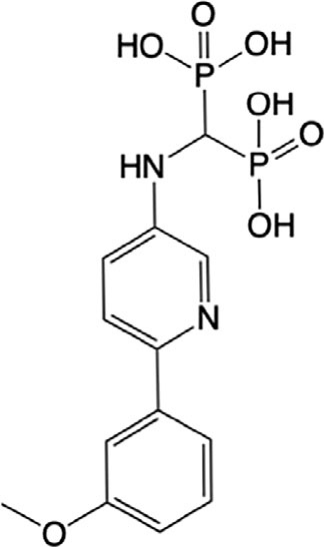	**20** (**9a**, ref. [35])	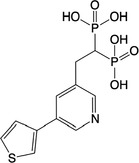
**21** (**33**, ref. [39])	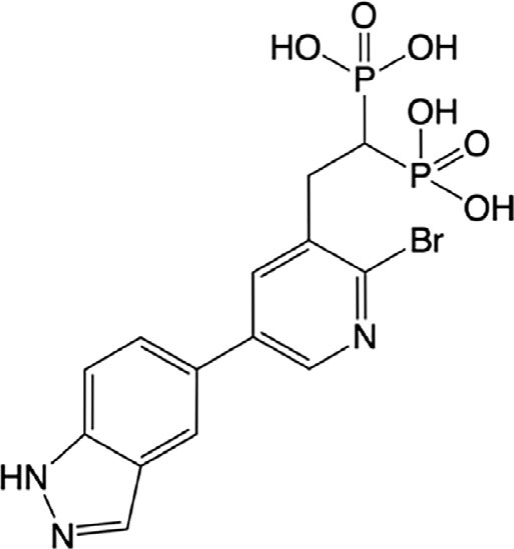	**22** (**9f**, ref. [34])	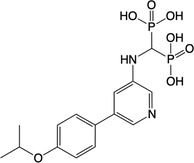
**23** (**9 h**, ref. [34])	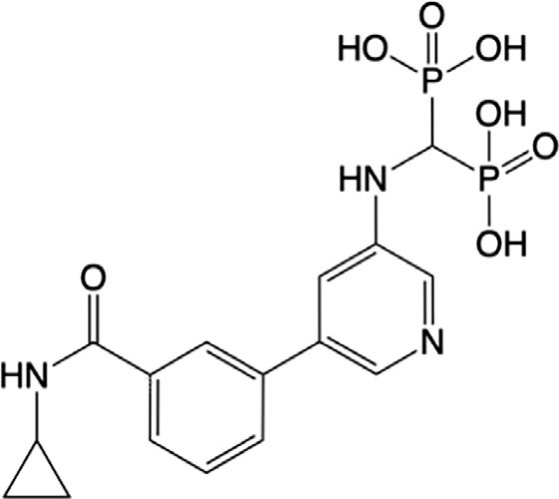	**24**	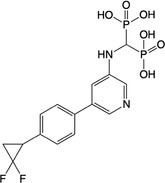

#### General procedure for Suzuki coupling

*Method A*: To a pressure vessel containing the heterocyclic bromide (1 eq), Pd(PPh_3_)_4_ (10 mol%), K_3_PO_4_ (2 eq), and aryl boronic acid pinacol ester (1.3 eq) under Ar, was added 4 : 1 DMF:water (25 mL per mmol of heterocyclic bromide). The reaction mixture was stirred at 80 °C for 3 h. The mixture was diluted with EtOAc, washed with NaHCO_3_ (aq) and brine, then concentrated. The crude product was purified by column chromatography (5% MeOH in DCM), dried, and subjected to the next step (McKenna reaction).

*Method B*: To a round bottom flask containing the heterocyclic bromide (1 eq), Pd(PPh_3_)_4_ (4 mol%), K_2_CO_3_ (2.2 eq), and aryl boronic acid (1.05 eq) under Ar, was added 1 : 1 water:dioxane (25 mL per mmol of heterocyclic bromide). The reaction mixture was stirred at 100 °C for 3–5 h, until reaction was complete as determined by TLC. The mixture was washed with NH_4_Cl and extracted twice with EtOAc. The combined organic layers were washed with water, brine, and dried over Na_2_SO_4_. The crude product was purified by column chromatography (0–50% EtOAc in hexanes), dried, and subjected to the next step (McKenna reaction).

*Method C*: A microwave reaction vessel was charged with Pd(PPh_3_)_4_ (0.1 eq), aryl boronic acid pinacol ester (1.5 eq), and heterocyclic bromide (1 eq) under Ar, and then dioxane was added such that the concentration of heterocyclic bromide was 0.1 m. The mixture was sparged with Ar, and then the vessel was sealed. The mixture was irradiated to 125 °C for 10–30 min (~ 40 W), and reaction progress was followed by TLC and/or HPLC. After completion, the reaction mixture was filtered through a plug of Celite, rinsed with 10 mL 1 : 1 EtOAc:acetone and concentrated under vacuum. The residue was purified by column chromatography (0–25% MeOH in EtOAc with 0.1% Et_3_N), dried, and subjected to the next step (McKenna reaction).

#### General procedure for deprotection of phosphonate ester via McKenna reaction

The phosphonate ester (1 eq) was dissolved in dry DCM (40 mL per mmol of phosphonate ester) under Ar. Then TMSBr (15 eq) was added dropwise. The reaction was tracked by ^31^P NMR and LC–MS. The mixture was stirred for 2–5 days, until complete transesterification had occurred. The mixture was concentrated in vacuo and added with MeOH (100 mL per mmol of phosphonate ester). The solvent was evaporated to dryness, and MeOH was added again. The addition of MeOH and evaporation were repeated a total of 5 times. The residue was suspended in a minimal amount of MeOH (~ 10 mL per mmol of phosphonate ester), and water (~ 100 mL per mmol of phosphonate ester) was added to precipitate the product. The precipitate was collected by filtration and washed with water and Et_2_O, to give the final compounds.

#### Compounds **9**–**14**

Compounds **9**–**14** were constructed using an SNAr and Suzuki coupling sequence as shown below. We have previously reported the synthesis of similar compounds using this route [30].

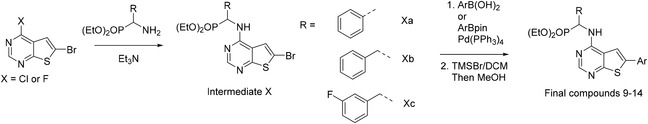

6‐bromo‐4‐chlorothieno[2,3‐d]pyrimidine and intermediate **Xa** have been previously reported in ref. [26] (compounds **14** and **17b** in that reference, respectively). 6‐bromo‐4‐fluorothieno[2,3‐d]pyrimidine has been previously reported in ref. [30] (compound **56a** in that reference).

#### Intermediate **Xb** (diethyl (1‐((6‐bromothieno[2,3‐d]pyrimidin‐4‐yl)amino)‐2‐phenylethyl)phosphonate)

6‐bromo‐4‐chlorothieno[2,3‐d]pyrimidine (200 mg, 0.8 mmol), *rac‐*diethyl (1‐amino‐2‐phenylethyl)phosphonate [30] (617 mg, 2.4 mmol), and triethylamine (0.56 mL, 4 mmol) were dissolved in dry dioxane (7 mL) and stirred in a pressure vessel under Ar at 100 °C for 48 h. The reaction was tracked by TLC. After completion, the reaction was added with water and extracted with EtOAc. The crude was purified by column chromatography (50–80% EtOAc in hexanes) to give the title compound as a white solid (190 mg, 51%). ^1^H NMR (500 MHz, CDCl_3_) δ 8.34 (s, 1H), 7.72 (s, 1H), 7.40–7.35 (m, 1H), 7.31–7.25 (m, 2H), 7.22–7.16 (m, 2H), 7.16–7.09 (m, 1H), 5.50–5.39 (m, 1H), 4.27–4.08 (m, 3H), 4.00 (ddq, *J* = 10.1, 8.1, 7.0 Hz, 1H), 3.33 (ddd, *J* = 14.4, 7.5, 4.9 Hz, 1H), 3.20 (ddd, *J* = 14.2, 12.0, 10.2 Hz, 1H), 1.36 (t, *J* = 7.1 Hz, 3H), 1.12 (t, *J* = 7.0 Hz, 3H). ^31^P NMR (203 MHz, CDCl_3_) δ 24.09. [ESI^−^] *m/z*: 468.1 [M ‐ H]^−^.

#### Intermediate **Xc** (diethyl (1‐((6‐bromothieno[2,3‐d]pyrimidin‐4‐yl)amino)‐2‐(3‐fluorophenyl)ethyl)phosphonate)

In a round bottom flask, 6‐fluoro‐4‐chlorothieno[2,3‐d]pyrimidine (175 mg, 0.75 mmol), diethyl (1‐amino‐2‐(3‐fluorophenyl)ethyl)phosphonate [30] (227 mg, 0.83 mmol), and triethylamine (190 mg, 1.88 mmol) were dissolved in dry DMSO (3 mL) and stirred at 100 °C for 3 h. The reaction was monitored by LC–MS and TLC. The reaction mixture was cooled to room temperature, diluted with water, and extracted with chloroform. The organic extract was dried over sodium sulfate, concentrated under vacuum, and purified by column chromatography (70% EtOAc in hexanes) to give the title compound as a white solid (172.8 mg, 47%). ^1^H NMR (500 MHz, CDCl_3_) δ 8.34 (s, 1H), 7.72 (s, 1H), 7.46 (d, *J* = 9.1 Hz, 1H), 7.14 (td, *J* = 8.1, 6.4 Hz, 1H), 7.10–6.97 (m, 2H), 6.89–6.76 (m, 1H), 5.54–5.35 (m, 1H), 4.30–4.21 (m, 2H), 4.19–4.05 (m, 1H), 4.05–3.92 (m, 1H), 3.34–3.27 (m, 1H), 3.23–3.10 (m, 1H), 1.40 (t, *J* = 7.1 Hz, 3H), 1.14 (t, *J* = 7.1 Hz, 3H). ^13^C NMR (126 MHz, CDCl_3_) δ 167.6, 162.6 (d, *J* = 245.8 Hz), 155.3 (d, *J* = 4.7 Hz), 153.7, 139.4 (dd, *J* = 13.9, 7.5 Hz), 129.7 (d, *J* = 8.3 Hz), 124.8 (d, *J* = 2.8 Hz), 121.3, 117.4, 116.2 (d, *J* = 21.4 Hz), 113.7 (d, *J* = 21.0 Hz), 111.7, 63.7 (d, *J* = 7.1 Hz), 62.5 (d, *J* = 7.5 Hz), 47.2 (d, *J* = 157.7 Hz), 35.5, 30.9, 16.5 (d, *J* = 6.1 Hz), 16.3 (d, *J* = 5.7 Hz). ^31^P NMR (203 MHz, CDCl_3_) δ 23.7 [ESI^−^] *m/z*: 486.1 [M ‐ H]^−^.

#### Compound **9** ((2‐(3‐Fluorophenyl)‐1‐((6‐(3‐isobutoxy‐4‐methylphenyl)thieno[2,3‐d]pyrimidin‐4‐yl)amino)ethyl)phosphonic acid)

Following the general procedure for Suzuki coupling (Method A) and McKenna reaction, intermediate **Xc** (45 mg, 0.092 mmol) was reacted with (3‐isobutoxy‐4‐methylphenyl)boronic acid pinacol ester (35 mg, 0.12 mmol) and subsequently deprotected to give the title compound as a white solid (25.3 mg, 55% over 2 steps). ^1^H NMR (500 MHz, DMSO‐*d*_6_) δ 8.22 (s, 1H), 8.13 (d, *J* = 11.6 Hz, 2H), 7.28–7.17 (m, 2H), 7.16–7.07 (m, 4H), 6.90 (td, *J* = 8.6, 2.7 Hz, 1H), 5.05 (dddd, *J* = 16.3, 13.1, 9.7, 3.2 Hz, 1H), 3.87 (d, *J* = 6.4 Hz, 2H), 3.26 (dt, *J* = 14.6, 3.7 Hz, 1H), 3.07 (ddd, *J* = 14.4, 12.3, 6.6 Hz, 1H), 2.20 (s, 3H), 2.10 (dp, *J* = 13.2, 6.6 Hz, 1H), 1.05 (d, *J* = 6.7 Hz, 6H). ^31^P NMR (203 MHz, DMSO‐*d*_6_) δ 19.34. ^19^F NMR (471 MHz, DMSO‐*d*_6_) δ −113.81. ^13^C NMR (126 MHz, DMSO‐*d*_6_) δ 163.7, 161.9 (d, *J* = 242.9 Hz), 157.3, 156.3, 156.3, 152.8, 141.6 (dd, *J* = 16.2, 7.6 Hz), 138.6, 132.0, 131.2, 126.8, 124.9, 117.8, 117.5, 115.5 (d, *J* = 21.0 Hz), 115.0, 112.9 (d, *J* = 20.9 Hz), 107.8, 73.7, 48.9 (d, *J* = 151.8 Hz), 34.7 (d, *J* = 4.2 Hz), 28.1, 19.2, 15.8. [ESI^−^] *m/z*: 514.3 [M ‐ H]^−^.

#### Compound **10** ((1‐((6‐(3‐Cyclopropoxy‐4‐methylphenyl)thieno[2,3‐d]pyrimidin‐4‐yl)amino)‐2‐phenylethyl)phosphonic acid)

Following the general procedure for Suzuki coupling (Method A) and McKenna reaction, intermediate **Xb** (34 mg, 0.072 mmol) was reacted with (3‐cyclopropoxy‐4‐methylphenyl)boronic acid pinacol ester (22 mg, 0.079 mmol) and subsequently deprotected to give the title compound as a white solid (22.5 mg, 66% over 2 steps). ^1^H NMR (500 MHz, DMSO‐*d*_6_) *δ* 8.26 (d, *J* = 9.7 Hz, 1H), 8.24 (s, 1H), 8.17 (s, 1H), 7.47 (d, *J* = 1.8 Hz, 1H), 7.31–7.22 (m, 3H), 7.20–7.13 (m, 3H), 7.07 (tt, *J* = 8.6, 1.2 Hz, 1H), 5.05 (dddd, *J* = 15.5, 12.5, 9.3, 3.0 Hz, 1H), 3.97 (tt, *J* = 6.0, 2.9 Hz, 1H), 3.25 (ddd, *J* = 14.4, 4.9, 2.8 Hz, 1H), 3.07 (ddd, *J* = 14.4, 12.2, 6.2 Hz, 1H), 2.13 (s, 3H), 0.93–0.81 (m, 2H), 0.78–0.65 (m, 2H). ^31^P NMR (203 MHz, DMSO‐*d*_6_) δ 19.59. ^13^C NMR (126 MHz, DMSO‐*d*_6_) δ 163.0, 157.3, 156.2 (d, *J* = 3.0 Hz), 152.5, 138.7, 138.5, 131.9, 131.3, 128.8, 128.1, 126.6, 126.1, 118.1, 117.5, 115.2, 109.4, 50.8, 49.4 (d, *J* = 151.5 Hz), 34.8 (d, *J* = 3.5 Hz), 15.7, 6.2. [ESI^−^] *m/z*: 480.2 [M ‐ H]^−^.

#### Compound **11** ((phenyl((6‐(4‐(trifluoromethyl)phenyl)thieno[2,3‐d]pyrimidin‐4‐yl)amino)methyl)phosphonic acid)

Following the general procedure for Suzuki coupling (Method B) and McKenna reaction, intermediate **Xa** (36 mg, 0.080 mmol) was reacted with (4‐(trifluoromethyl)phenyl)boronic acid (16 mg, 0.084 mmol) and subsequently deprotected to give the title compound as a beige powder (22.2 mg, 60% over 2 steps). ^1^H NMR (800 MHz, 0.5% ND_4_OD in D_2_O) δ 7.79 (s, 1H), 7.64 (d, *J* = 7.3 Hz, 3H), 7.52 (t, *J* = 7.7 Hz, 2H), 7.39 (t, *J* = 7.3 Hz, 1H), 7.17 (d, *J* = 7.4 Hz, 2H), 6.90 (d, *J* = 7.7 Hz, 2H), 5.11 (d, *J* = 19.4 Hz, 1H). ^31^P NMR (203 MHz, 0.5% ND_4_OD in D_2_O) δ 14.04. ^19^F NMR (470 MHz, 0.5% ND_4_OD in D_2_O) δ −62.32. ^13^C NMR (201 MHz, 0.5% ND_4_OD in D_2_O) δ 163.2, 156.0, 152.7, 141.0, 138.1, 135.2, 128.4 (q, *J* = 32.0 Hz), 128.2, 127.9, 126.7, 125.2, 124.9, 123.7 (q, *J* = 271.7 Hz), 118.2, 115.6, 55.6 (d, *J* = 131.2 Hz). [ESI^−^] *m/z*: 464.1 [M ‐ H]^−^.

#### Compound **12** ((((6‐(4‐fluorophenyl)thieno[2,3‐d]pyrimidin‐4‐yl)amino)(phenyl)methyl)phosphonic acid)

Following the general procedure for Suzuki coupling (Method B) and McKenna reaction, intermediate **Xa** (36 mg, 0.080 mmol) was reacted with (4‐fluorophenyl)boronic acid (12 mg, 0.084 mmol) and subsequently deprotected to give the title compound as a white solid (21.1 mg, 32% over 2 steps). ^1^H NMR (800 MHz, 0.5% ND_4_OD in D_2_O) δ 7.69 (s, 1H), 7.64–7.59 (m, 3H), 7.48 (t, *J* = 7.7 Hz, 2H), 7.36 (t, *J* = 7.4 Hz, 1H), 7.10 (t, *J* = 6.6 Hz, 2H), 6.60 (t, *J* = 8.5 Hz, 2H), 5.09 (d, *J* = 19.5 Hz, 1H). ^31^P NMR (203 MHz, 0.5% ND_4_OD in D_2_O) δ 13.98. ^19^F NMR (470 MHz, 0.5% ND_4_OD in D_2_O) δ −113.89 (tt, *J* = 9.1, 5.3 Hz). ^13^C NMR (201 MHz, 0.5% ND_4_OD in D_2_O) δ 162.8, 162.0 (d, *J* = 246.5 Hz), 155.9 (d, *J* = 9.2 Hz), 152.2, 141.0, 139.0, 128.4 (d, *J* = 3.0 Hz), 128.2, 127.8 (d, *J* = 3.7 Hz), 126.9 (d, *J* = 8.3 Hz), 126.6, 118.4, 115.4 (d, *J* = 22.1 Hz), 113.6, 55.6 (d, *J* = 130.2 Hz). [ESI^−^] *m/z*: 414.1 [M ‐ H]^−^.

#### Compound **13** ((((6‐(2,6‐dimethoxyphenyl)thieno[2,3‐d]pyrimidin‐4‐yl)amino)(phenyl)methyl)phosphonic acid)

Following the general procedure for Suzuki coupling (Method B) and McKenna reaction, intermediate **Xa** (36 mg, 0.080 mmol) was reacted with (2,6‐dimethoxyphenyl)boronic acid (15 mg, 0.084 mmol) and subsequently deprotected to give the title compound as a beige powder (21.3 mg, 39% over 2 steps). ^1^H NMR (800 MHz, 0.5% ND_4_OD in D_2_O) δ 8.14 (s, 1H), 7.87 (s, 1H), 7.52 (d, *J* = 7.7 Hz, 2H), 7.37 (t, *J* = 8.5 Hz, 1H), 7.35 (t, *J* = 7.6 Hz, 2H), 7.26 (t, *J* = 7.4 Hz, 1H), 6.79 (d, *J* = 8.4 Hz, 2H), 5.17 (d, *J* = 20.0 Hz, 1H), 3.84 (s, 6H). ^31^P NMR (203 MHz, 0.5% ND_4_OD in D_2_O) δ 13.70. ^13^C NMR (201 MHz, 0.5% ND_4_OD in D_2_O) δ 164.4, 157.5, 156.4 (d, *J* = 9.4 Hz), 152.9, 140.5, 131.1, 130.8, 128.1, 127.3 (d, *J* = 3.8 Hz), 126.4 (d, *J* = 2.4 Hz), 120.1, 117.1, 110.5, 105.1, 56.1, 55.8 (d, *J* = 128.5 Hz). [ESI^−^] *m/z*: 456.2 [M ‐ H]^−^.

#### Compound **14** ((phenyl((6‐(3‐(trifluoromethyl)phenyl)thieno[2,3‐d]pyrimidin‐4‐yl)amino)methyl)phosphonic acid)

Following the general procedure for Suzuki coupling (Method B) and McKenna reaction, intermediate **Xa** (36 mg, 0.080 mmol) was reacted with (3‐(trifluoromethyl)phenyl)boronic acid (16 mg, 0.084 mmol) and subsequently deprotected to give the title compound as a beige powder (32.6 mg). ^1^H NMR (800 MHz, 0.5% ND_4_OD in D_2_O) δ 7.76 (d, *J* = 11.2 Hz, 2H), 7.62 (s, 1H), 7.59 (d, *J* = 8.2 Hz, 2H), 7.45 (t, *J* = 7.6 Hz, 2H), 7.34 (t, *J* = 7.3 Hz, 1H), 7.25 (d, *J* = 7.8 Hz, 1H), 7.19 (d, *J* = 7.6 Hz, 1H), 6.93 (t, *J* = 8.0 Hz, 1H), 5.09 (d, *J* = 19.6 Hz, 1H). ^31^P NMR (203 MHz, 0.5% ND_4_OD in D_2_O) δ 13.80. ^19^F NMR (470 MHz, 0.5% ND_4_OD in D_2_O) δ −62.66. ^13^C NMR (201 MHz, 0.5% ND_4_OD in D_2_O) δ 163.2, 156.1 (d, *J* = 9.2 Hz), 152.8, 140.8, 138.2, 132.8, 130.0 (q, *J* = 32.4 Hz), 129.5, 128.6, 128.2, 127.7, 126.6, 124.4, 123.8 (q, *J* = 271.4 Hz), 121.3, 118.2, 115.1, 55.5 (d, *J* = 129.7 Hz). [ESI^−^] *m/z*: 464.1 [M ‐ H]^−^.

#### Compound **16** (2‐(1‐Methyl‐5‐(4‐(trifluoromethyl)phenyl)‐1H‐indol‐3‐yl)ethylphosphonic acid)

This compound was constructed from the corresponding heterocyclic aldehyde as shown below, in a sequence similar to that reported in the literature [26].

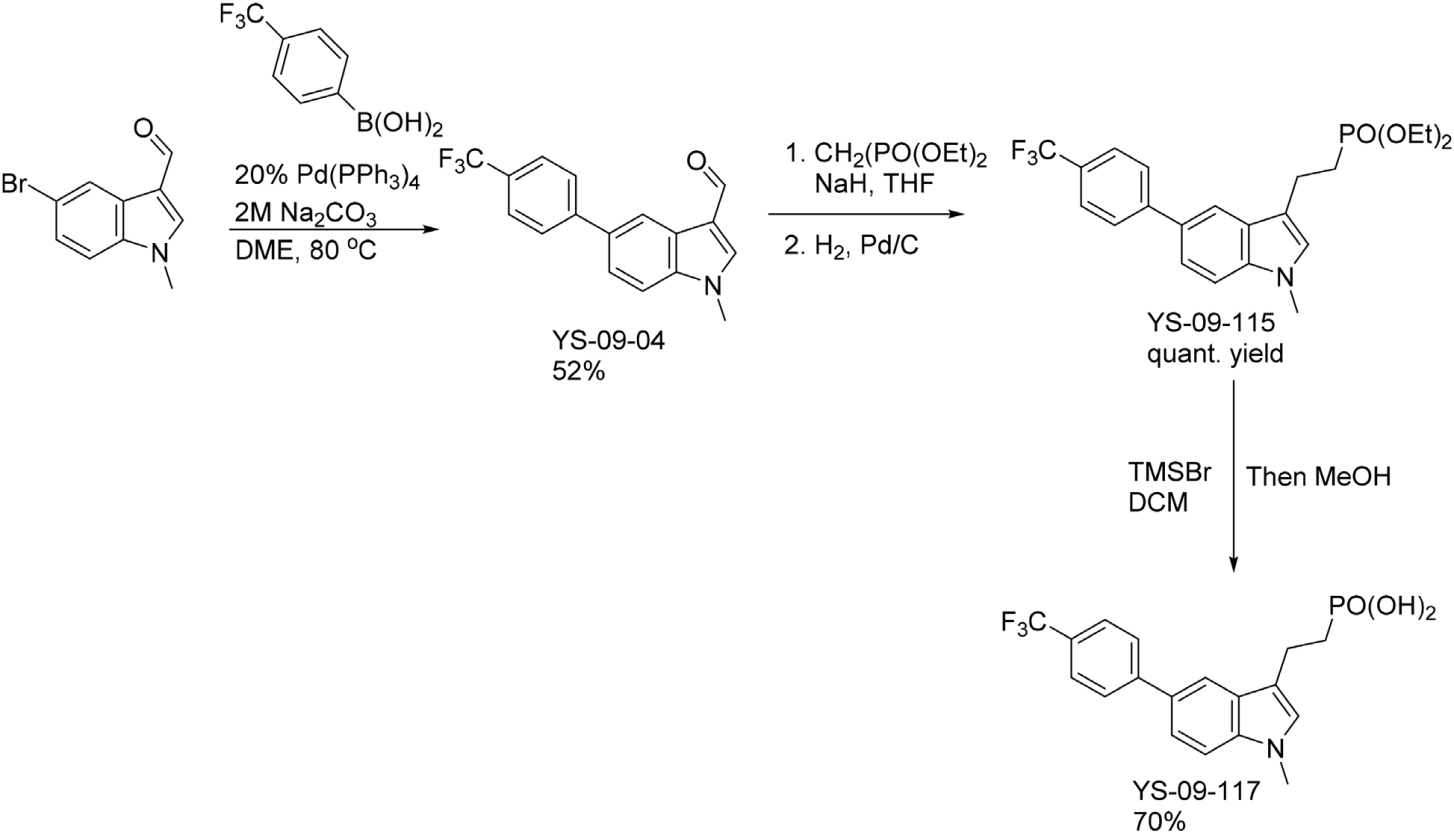

#### Intermediate **YS‐09‐04** (1‐methyl‐5‐(4‐(trifluoromethyl)phenyl)‐1H‐indole‐3‐carbaldehyde)

In a vial under Ar atmosphere, a mixture of 5‐bromo‐1‐methyl‐1H‐indole‐3‐carbaldehyde (synthesis of which is reported in the literature [37]; also commercially available) (60 mg, 0.25 mmol, 1 eq) and Pd(PPh_3_)_4_ (43.7 mg, 0.038 mmol, 0.15 eq) were suspended in DME and stirred for 15 min to give a yellow solution. A solution of 4‐trifluoromethylphenylboronic acid (57 mg, 0.3 mmol, 1.2 eq) in EtOH (0.5 mL) was added. The solution was stirred at RT for another 15 min. The reaction mixture was added with 2 m sodium carbonate solution (0.32 mL, 0.63 mmol, 2.5 eq) and heated at 80 °C for 16 h. The reaction mixture was filtered through Celite and rinsed with EtOAc three times. The filtrate was concentrated and purified by chromatography on silica gel (0–80% EtOAc in hexanes) to give the title compound as a light yellow solid (40 mg, 52%). ^1^H NMR (300 MHz, CDCl_3_) δ 10.00 (s, 1H), 8.56 (s, 1H), 7.77 (d, *J* = 8.0 Hz, 2H), 7.67–7.69 (m, 3H), 7.59 (d, *J* = 8.6 Hz, 1H), 7.43 (d, *J* = 8.6 Hz, 1H), 3.89 (s, 3H); ^13^C NMR (75 MHz, CDCl_3_) δ 184.4, 145.0, 140.0, 137.7, 134.9, 129.5, 128.6, 127.7, 125.6 (q, *J* = 3.8 Hz), 124.2 (q, *J* = 270.0 Hz), 123.6, 120.9, 118.4, 110.4, 33.8. [ESI^+^] *m/z*: 304.1 [M + H]^+^.

#### Intermediate **YS‐09‐115** (diethyl (2‐(1‐methyl‐5‐(4‐(trifluoromethyl)phenyl)‐1H‐indol‐3‐yl)ethyl)phosphonate)

In a flask, a solution of tetraethyl methylenediphosphonate (265 mg, 0.92 mmol, 1 eq) in THF (3 mL) was cooled to 0 °C, added with NaH (44 mg 60% dispersion in mineral oil, 1.1 mmol, 1.2 eq), and stirred at 0 °C for 15 min. A solution of 1‐methyl‐5‐(4‐(trifluoromethyl)phenyl)‐1H‐indole‐3‐carbaldehyde (intermediate **YS‐09‐04**) (307 mg, 1.01 mmol, 1.1 eq) dissolved in THF (1 mL) was added. The resulting solution was stirred at room temperature for 1 h. The reaction was quenched with water, diluted with EtOAc, and washed with brine. The organic layer was collected, dried over MgSO_4_, concentrated, and purified by chromatography on silica gel (0–10% MeOH in EtOAc) to isolate the olefinic intermediate as a white solid (408 mg, quant yield). 250 mg of this intermediate was stirred with 10% Pd/C (30 mg) in EtOH (3 mL) under an atmosphere of H_2_ for 30 min. The solution was passed through Celite and washed with MeOH. The filtrate was concentrated to give the title compound as a white solid (268 mg, quant. yield). ^1^H NMR (500 MHz, CDCl_3_) δ 7.79 (dd, *J* = 1.7, 0.6 Hz, 1H), 7.75 (d, *J* = 8.1 Hz, 2H), 7.68 (d, *J* = 8.1 Hz, 2H), 7.48 (dd, *J* = 8.5, 1.7 Hz, 1H), 7.36 (dd, *J* = 8.5, 0.6 Hz, 1H), 6.93 (s, 1H), 4.01–4.17 (m, 4H), 3.77 (s, 3H), 3.05–3.16 (m, 2H), 2.14–2.21 (m, 2H), 1.33 (t, *J* = 7.1 Hz, 6H); ^13^C NMR (125 MHz, CDCl_3_) δ 146.0, 137.0, 130.9, 127.8, 127.5, 127.1, 125.6 (q, *J* = 3.7 Hz), 124.9 (q, *J* = 227.3 Hz), 121.4, 117.6, 114.8, 114.6, 109.8, 61.6 (d, *J* = 6.4 Hz), 32.8, 26.7 (d, *J* = 138.6 Hz), 18.3 (d, *J* = 4.3 Hz), 16.5 (d, *J* = 6.0 Hz). [ESI^+^] *m/z*: 440.3 [M + H]^+^.

#### Compound **16** (2‐(1‐Methyl‐5‐(4‐(trifluoromethyl)phenyl)‐1H‐indol‐3‐yl)ethylphosphonic acid)

A solution of diethyl (2‐(1‐methyl‐5‐(4‐(trifluoromethyl)phenyl)‐1H‐indol‐3‐yl)ethyl)phosphonate (intermediate **YS‐09‐115**) (48 mg, 0.11 mmol, 1 eq) in CH_2_Cl_2_ (3 mL) was treated with TMSBr (144 μL, 167 mg, 1.1 mmol, 10 eq) and stirred at room temperature for 3 days. The solution was added with MeOH and stirred for 1 h. The volatiles were removed under reduced pressure. The material was suspended in MeOH and triturated with CH_2_Cl_2_ to give the title compound as a white solid (29.5 mg, 70%). ^1^H NMR (500 MHz, D_2_O) δ 7.91 (s, 1H), 7.58 (d, *J* = 8.2 Hz, 2H), 7.50 (d, *J* = 8.2 Hz, 2H), 7.13–7.03 (m, 2H), 7.02 (s, 1H), 3.53 (s, 3H), 2.86–2.81 (m, 2H), 1.75–1.61 (m, 2H); ^13^C NMR (125 MHz, D_2_O) δ 145.2, 136.8, 129.6, 127.5, 127.0, 125.5, 125.0 (q, *J* = 233.8 Hz), 123.4, 120.7, 117.5, 117.1, 116.9, 110.0 (m), 31.8, 30.6 (d, *J* = 128.8 Hz), 19.8; ^31^P NMR (81 MHz, D_2_O) δ 21.52. [ESI^−^] *m/z*: 382.1 [M ‐ H]^−^.

#### Compound **19** ((((6‐(3‐methoxyphenyl)pyridin‐3‐yl)amino)methylene)bis(phosphonic acid))

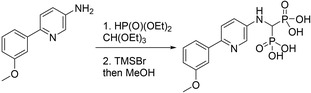

The synthetic route to compound **19** was previously outlined by De Schutter *et al*. [33]. The experimental procedure is as follows:

##### Step 1

To a 15 mL pressure vessel was added, in sequence, 6‐(3‐methoxyphenyl)pyridin‐3‐amine [38] (60 mg, 0.3 mmol, 1 eq), diethyl phosphite (83 mg, 0.6 mmol, 2 eq) and triethyl orthoformate (44 mg, 0.3 mmol, 1 eq). The vessel was tightly sealed, and the reaction mixture was stirred overnight at 110 °C. The reaction mixture was allowed to cool to room temperature and concentrated in vacuo. The residue was purified by normal phase column chromatography on silica gel (silica gel was first deactivated by eluting with 66 : 33 : 1 hexanes : EtOAc : Et_3_N) in a gradient from 100% hexanes to 100% EtOAc and then to 25% MeOH, to give the tetraethyl ester precursor (146 mg, 64%). ^1^H NMR (300 MHz, CDCl_3_) δ 8.20 (d, *J =* 2.8, 1H), 7.58 (d, *J =* 8.7, 1H), 7.53–7.43 (m, 2H), 7.33 (t, *J =* 7.9, 1H), 7.07 (dd, *J =* 2.9, 8.6, 1H), 6.89 (dd, *J =* 2.6, 8.1, 1H), 4.32–4.11 (m, 9H), 3.88 (s, 3H), 1.29 (dt, *J =* 7.1, 12.5, 12H). ^31^P NMR (81 MHz, CDCl_3_) δ 14.76. [ESI^+^] *m/z*: 468.1 [M + Na]^+^.

##### Step 2

The tetraethyl ester precursor (90 mg, 0.19 mmol) was deprotected following the general procedure for McKenna reaction. After filtration, the solid was washed with water, MeCN and Et_2_O, then dried, to give compound **19** as a pale yellow powder (44 mg, 63%). ^1^H NMR (800 MHz, 0.5% ND_4_OD in D_2_O) δ 8.09 (d, *J* = 2.9 Hz, 1H), 7.67 (d, *J* = 8.7 Hz, 1H), 7.48–7.41 (m, 3H), 7.26 (dd, *J* = 8.8, 2.9 Hz, 1H), 7.01 (dt, *J* = 6.7, 2.6 Hz, 1H), 3.91 (s, 3H), 3.72 (t, *J* = 19.0 Hz, 1H). ^31^P NMR (203 MHz, 0.5% ND_4_OD in D_2_O) δ 15.13. ^13^C NMR (201 MHz, 0.5% ND_4_OD in D_2_O) δ 159.3, 144.9 (t, *J* = 4.0 Hz), 143.6, 140.9, 134.8, 130.2, 122.2, 120.1, 118.8, 113.5, 111.1, 55.4, 52.6 (t, *J* = 125.2 Hz). HRMS (ESI^−^): calcd 373.03545 (C_13_H_15_N_2_O_7_P_2_), found *m/z* = 373.03593 [M ‐ H]^−^.

#### Compound **21** ((2‐(2‐bromo‐5‐(1H‐indazol‐5‐yl)pyridin‐3‐yl)ethane‐1,1‐diyl)bis(phosphonic acid))

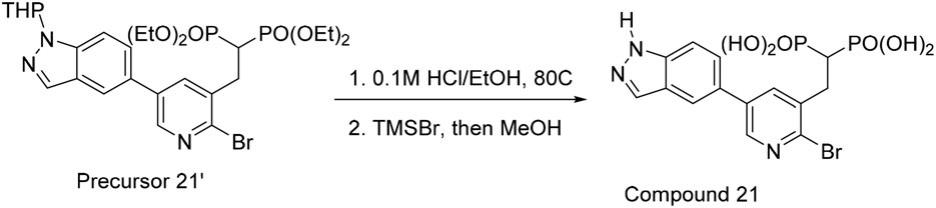

The synthesis of precursor **21′** was previously reported by Tsantrizos *et al*. [39].

##### Step 1

A 15 mL pressure vessel was charged with precursor **21′** (100 mg, 0.15 mmol) and dry EtOH (3.4 mL). Ethanolic HCl (0.38 mL, 0.38 mmol, 2.5 eq) was added by syringe such that the final concentration of HCl was 0.1 m. The reaction mixture was stirred at 80 °C for 12 h. The mixture was concentrated under vacuum, re‐dissolved in 100 mL EtOAc, and washed with 10 mL saturated NaHCO_3_ and 10 mL brine, dried over anhydrous Na_2_SO_4_, filtered, concentrated, and purified by column chromatography on silica gel (pre‐washed with a dilute solution of NEt_3_ in EtOAc; compound eluted using 10% MeOH in EtOAc) to obtain the tetraester intermediate as a colorless oil (81 mg, 92%). ^1^H NMR (400 MHz, CDCl_3_) δ 8.51 (s, 1H), 8.14 (s, 1H), 8.00 (s, 1H), 7.94 (s, 1H), 7.57 (s, 2H), 4.26–4.00 (m, 8H), 3.50–3.35 (m, 2H), 3.17 (m, 1H), 1.26 (dt, *J =* 25.5, 7.0 Hz, 12H). ^13^C NMR (75 MHz, CD_3_OD) δ 146.0, 141.2, 139.8, 139.4, 136.6, 134.9 (t, *J =* 9.2 Hz), 134.2, 128.7, 125.7, 123.6, 119.1, 110.8, 63.0 (dd, *J =* 29.9, 6.9 Hz), 35.3 (t, *J =* 134.3 Hz), 31.2 (t, *J =* 4.5 Hz), 15.2 (d, *J =* 6.3 Hz). ^31^P NMR (81 MHz, CDCl_3_) δ 21.74. [ESI^+^] *m/z*: 596.0 [M + Na]^+^.

##### Step 2

The tetraester precursor (80 mg, 0.14 mmol) was deprotected following the general procedure for McKenna reaction. After repeated addition and evaporation of MeOH (according to the general procedure), the residue was suspended in EtOH, precipitated with water, triturated with EtOH:water (1 : 1) twice and then EtOH/Et_2_O (1 : 1) twice, and finally dried to give compound **21** as a white powder (22 mg, 34%). ^1^H NMR (800 MHz, 0.5% ND_4_OD in D_2_O) δ 8.37–8.31 (m, 1H), 8.20–8.14 (m, 2H), 8.07 (s, 1H), 7.74 (dt, *J* = 8.7, 2.5 Hz, 1H), 7.70 (dd, *J* = 8.7, 3.0 Hz, 1H), 3.26 (td, *J* = 14.5, 7.6 Hz, 2H), 2.49 (tt, *J* = 20.3, 7.3 Hz, 1H). ^31^P NMR (203 MHz, 0.5% ND_4_OD in D_2_O) δ 18.22. ^13^C NMR (201 MHz, 0.5% ND_4_OD in D_2_O) δ 144.8, 141.0, 139.7, 139.1, 138.3 (t, *J* = 8.8 Hz), 135.9, 134.7, 129.1, 126.7, 123.0, 119.5, 111.1, 39.8–37.7 (m), 31.7, 29.6. MS (ESI): calcd 459.95 and 461.94, found 460.1 and 462.0 [M ‐ H]^−^.

#### Compound **24** ((((5‐(4‐(2,2‐difluorocyclopropyl)phenyl)pyridin‐3‐yl)amino)methylene)bis(phosphonic acid))

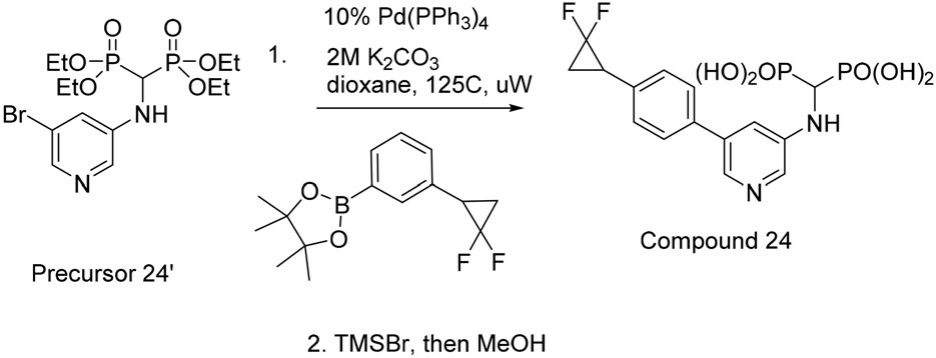

The synthesis of precursor **24′** was previously reported by De Schutter *et al*. [34]. Following the general procedure for Suzuki coupling (Method C) and McKenna reaction, precursor **24′** (50 mg, 0.11 mmol) was reacted with (4‐(2,2‐difluorocyclopropyl)phenyl)boronic acid pinacol ester [40] (46 mg, 0.16 mmol) and subsequently deprotected to give the title compound as a white powder (24.4 mg, 60% over 2 steps). ^1^H NMR (800 MHz, 0.5% ND_4_OD in D_2_O) δ 8.04 (dd, *J* = 6.2, 2.2 Hz, 2H), 7.72 (d, *J* = 8.2 Hz, 2H), 7.47–7.43 (m, 3H), 3.81 (t, *J* = 18.7 Hz, 1H), 2.99 (td, *J* = 12.6, 8.2 Hz, 1H), 1.97 (tdd, *J* = 12.1, 8.3, 5.3 Hz, 1H), 1.85 (dtd, *J* = 12.4, 8.2, 3.8 Hz, 1H). ^31^P NMR (203 MHz, 0.5% ND_4_OD in D_2_O) δ 14.96. ^19^F NMR (470 MHz, 0.5% ND_4_OD in D_2_O) δ −126.44 (dtd, *J* = 151.9, 13.2, 3.9 Hz), −142.55 (ddd, *J* = 151.8, 13.5, 5.6 Hz). ^13^C NMR (201 MHz, 0.5% ND_4_OD in D_2_O) δ 145.3 (t, *J* = 4.3 Hz), 136.7, 136.4, 134.6, 134.0, 133.9, 128.6, 127.2, 117.9, 113.5 (dd, *J* = 287.2, 281.1 Hz), 52.1 (t, *J* = 122.9 Hz), 26.4 (t, *J* = 11.1 Hz), 16.1 (t, *J* = 10.2 Hz). HRMS: calcd 419.03734 (C_15_H_15_F_2_N_2_O_6_P_2_), found *m*/*z* 419.03908 [M ‐ H]^−^.

### Inhibitor screening

To prepare master stock solutions, the phosphonate compounds were dissolved in Milli‐Q water to a concentration of 20 mm, either as monosodium salts (monophosphonate compounds) or as trisodium salts (bisphosphonate compounds). Their effects on MK activity were evaluated by using the coupled enzyme assay described above. Each compound was tested at two concentrations: 1 and 10 μm. MVA and ATP were both added at a concentration of 0.5 μm. Percent inhibition was calculated by comparing the initial rate of the test reaction to that of a control reaction that did not include any test compound.

The control assay to examine potential inhibition of the reporter enzymes consisted of the following components: 50 mm HEPES (pH 7.5), 100 mm KCl, 10 mm MgCl_2_, 0.15 mm phosphoenolpyruvate, 0.15 mm NADH, 0.15 mm ADP, ~ 8 units of pyruvate kinase, ~ 12 units of lactate dehydrogenase, and 10 μm test compound when added. All other reaction conditions remained identical to the standard MK‐coupled assay; however, each reaction was carried out in duplicate instead of triplicate.

To determine the *IC*_50_, a more refined inhibition response across a broader range of inhibitor concentrations was measured. Typically, nine different inhibitor concentrations spanning a 10 000‐fold concentration range were used. Dose–response curves were generated by fitting the inhibition data to the following three‐parameter dose–response equation (Eqn 2), where *Max* and *Min* denote the maximum and minimum response, respectively:

$$\text{Response}=\mathrm{Min}+\frac{\mathrm{Max}-\mathrm{Min}}{1+\left(\left[I\right]/{\mathrm{IC}}_{50}\right)}.$$

Data analysis was performed with graphpad prism software.

### Mode of inhibition and *K*_i_ determination

Initial rate experiments were conducted to determine the *K*_i_ values of the most potent MK inhibitors. In all experiments, one substrate was added at a constant concentration of 5 mm. When ATP was held constant, MVA was varied from 0.01 to 0.2 mm. When MVA was held constant, ATP was varied from 0.05 to 1 mm. Each inhibitor was tested at four different concentrations ranging from 0 to 200 nm. Data analysis was performed with graphpad prism software. The initial rates were fitted to steady‐state equations describing competitive inhibition model (Eqn 3),

$$v_{i}=\frac{V_{\max}\left[S\right]}{K_{m}\left(1+\left[I\right]/K_{i}\right)+\left[S\right]},$$

noncompetitive inhibition model (Eqn 4),

$$v_{i}=\frac{\frac{V_{\max}\left[S\right]}{1+\left[I\right]/K_{i}}}{K_{m}+\left[S\right]},$$

uncompetitive inhibition model (Eqn 5),

$$v_{i}=\frac{\frac{V_{\max}\left[S\right]}{1+\left[I\right]/{K_{i}}^{\prime }}}{\frac{K_{m}}{1+\left[I\right]/{K_{i}}^{\prime }}+\left[S\right]},$$

and mixed inhibition model (Eqn 6),

$$v_{i}=\frac{\frac{V_{\max}\left[S\right]}{1+\left[I\right]/{K_{i}}^{\prime }}}{\frac{K_{m}\left(1+\left[I\right]/K_{i}\right)}{1+\left[I\right]/{K_{i}}^{\prime }}+\left[S\right]},$$

where [*S*] represents the concentration of the varied substrate. The selection of the most appropriate models was determined through the extra sum‐of‐squares *F* test as implemented in the software.

## Results

### Kinetic characterization of human MK

**Fig. 3:**
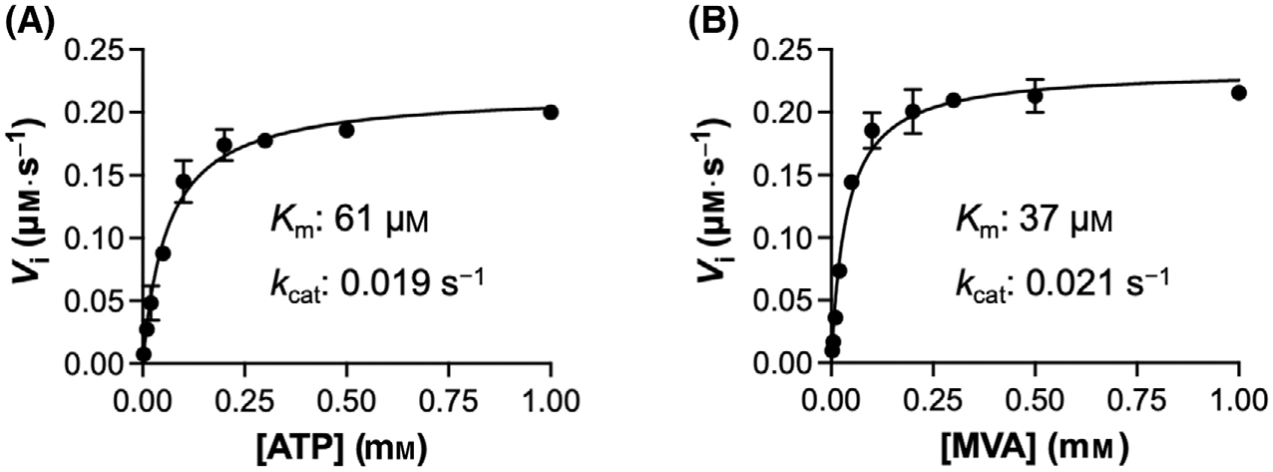
Steady‐state kinetics of human MK. (A) Initial rates plotted against ATP concentrations, measured at a fixed concentration of 5 mm MVA. (B) Initial rates plotted against MVA concentrations, measured at a fixed concentration of 5 mm ATP. The presented kinetic parameters were determined by nonlinear regression to the Michaelis–Menten model (Eqn 1). All replicate data points were used for curve fitting. The goodness‐of‐fit values (*R* ^2^) were 0.9805 and 0.9806 for (A) and (B), respectively. Displayed data points represent mean ± standard deviation (*n* = 3).

To determine the substrate concentrations to use in the screening study, we first established the *K*_m_ of the recombinantly produced MK. In general, using concentrations near the *K*_m_ is considered optimal in enzyme assays for identifying inhibitors of unknown modalities [41]. The *K*_m_ values were determined to be 61 μm for ATP and 37 μm for MVA (Fig. 3), falling within a similar range to those previously reported (*K*_m_^ATP^ of 74 and 178.4 μm; *K*_m_^MVA^ of 24 and 40.8 μm) [19, 20]. A concentration of 50 μm was used for both ATP and MVA in the subsequent screening assays.

### Identification of MK inhibitors

Using the recombinantly produced enzyme, we conducted a screening study to identify new inhibitors of MK. We tested 24 compounds, comprising 16 monophosphonates (Table 1) and eight bisphosphonates (Table 2). Notably, the monophosphonates were synthesized as FPP mimetics in our effort to discover allosteric inhibitors of FPPS [26, 30, 31]. Conversely, the bisphosphonates were synthesized as active site inhibitors of FPPS that can mimic its substrate carbocation intermediates [35, 36, 42].

**Fig. 4:**
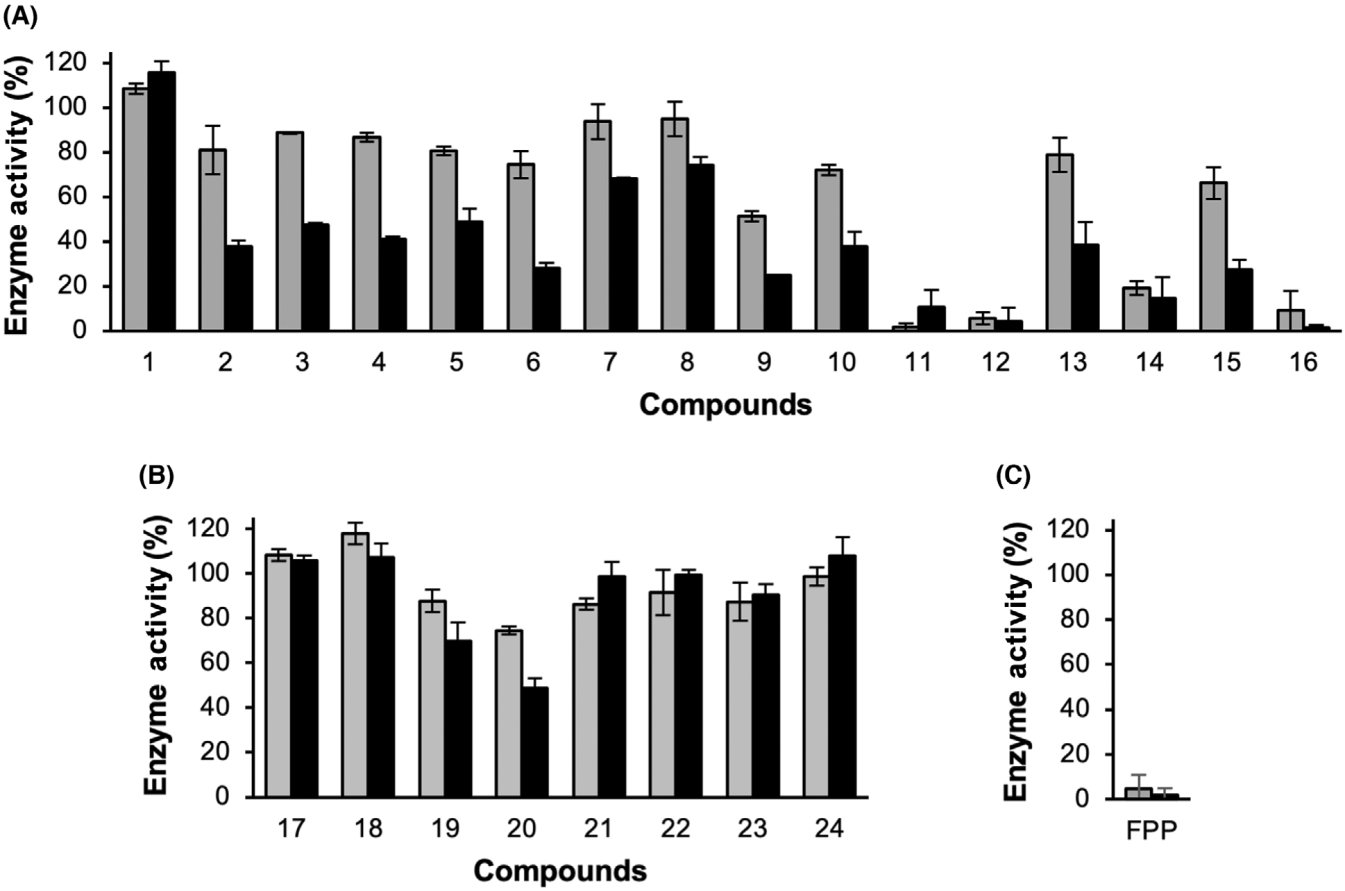
Screening for MK inhibitors. Inhibition of the MK reaction by monophosphonate compounds (A), bisphosphonate compounds (B), and FPP (C), tested at concentrations of 1 μm (gray columns) and 10 μm (black columns). Percent activity was calculated by comparison to the control reaction without any test compounds. Error bars represent standard deviation (*n* = 3).

Several compounds potently inhibited the MK catalytic reaction. Most monophosphonates showed inhibition, with three resulting in > 90% inhibition at 1 μm concentration (Fig. 4A). In contrast, bisphosphonates were mostly inactive, with only one resulting in > 50% inhibition at 10 μm concentration (Fig. 4B). FPP, included as a positive control, exhibited a similar level of inhibitory activity (96% inhibition at 1 μm; Fig. 4C) to the most potent test compounds.

**Fig. 5:**
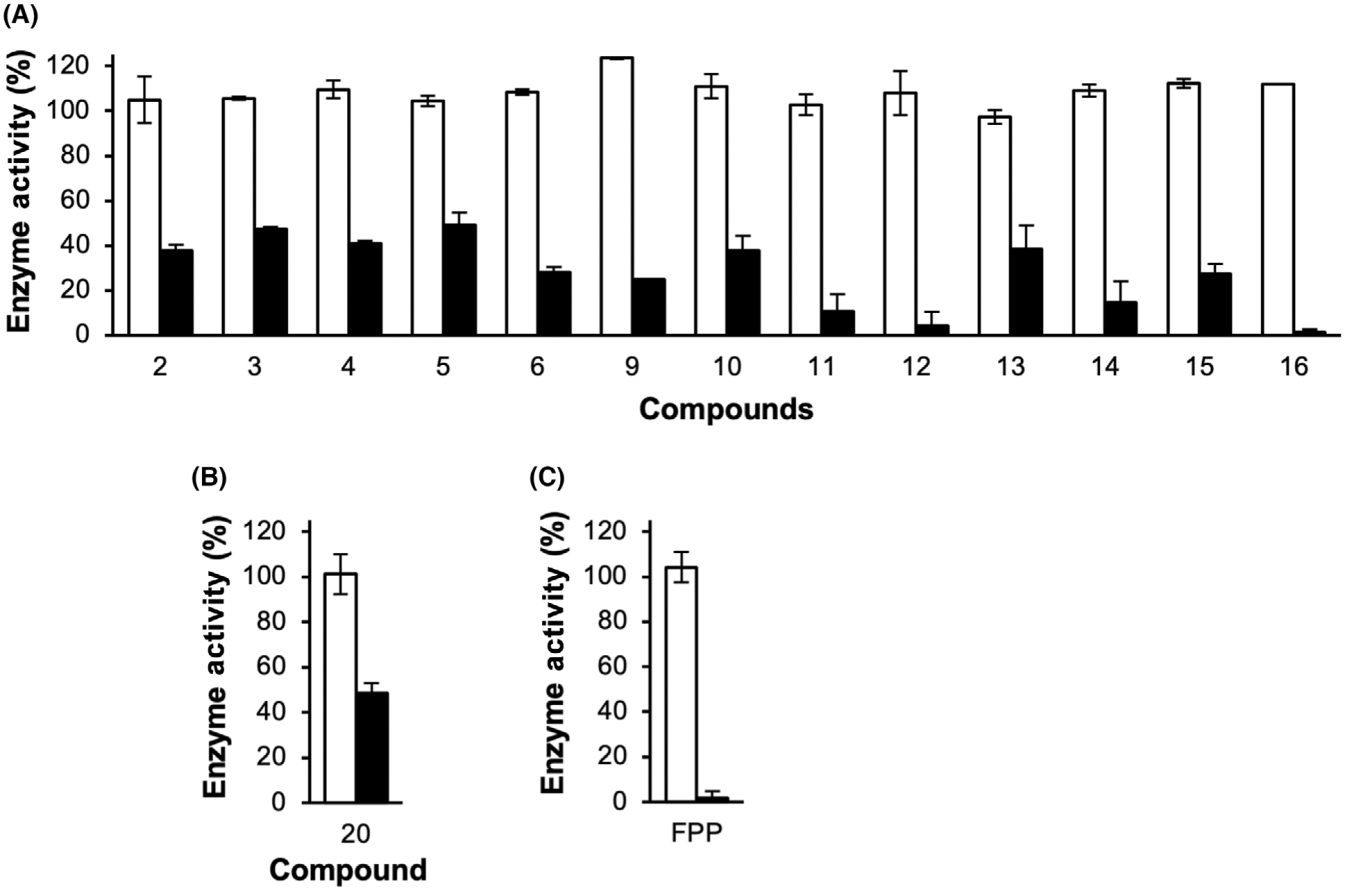
Effects of MK inhibitors on the MK‐omitted control reaction. White columns represent the % catalytic activity in the presence of 10 μm monophosphonates (A), bisphosphonates (B), and FPP (C). Error bars represent standard deviation (*n* = 2). For comparison, black columns show the inhibition of the MK‐coupled reaction at the same concentration of the test compounds (from Fig. 1; *n* = 3).

To confirm that the observed inhibition was specific to MK and not the reporter enzymes used in the assay, a control experiment was conducted. MK and its substrates were omitted from the assay, and instead, ADP was included to drive the MK‐decoupled enzyme reaction. Compounds that produced > 50% inhibition at 10 μm concentration in the previous screening experiment were tested, 14 in total. The results confirmed that these compounds specifically target MK, as they had no effect on the MK‐omitted control reaction (Fig. 5).

**Fig. 6:**
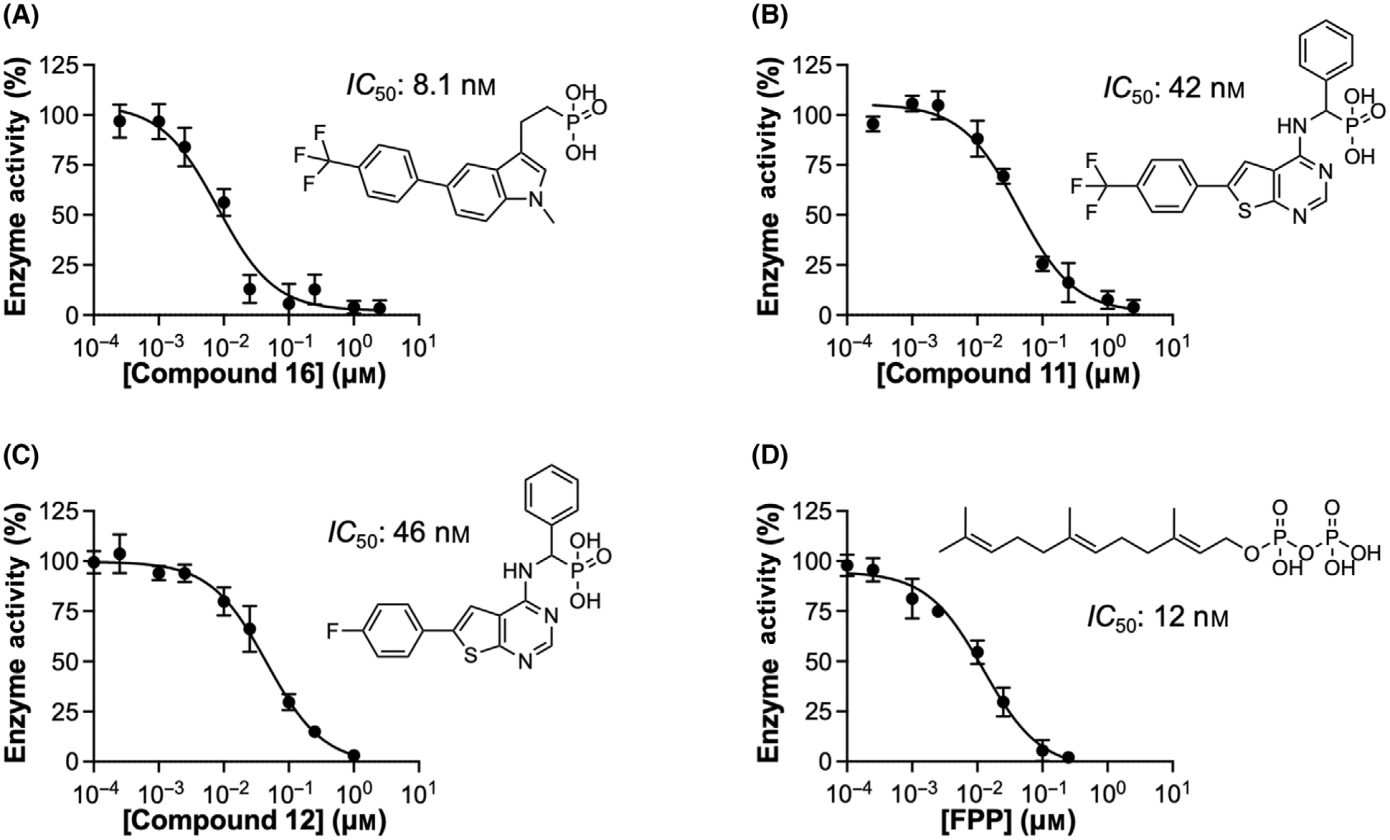
Potent inhibitors of human MK. Chemical structures, dose–response curves, and determined *IC*_50_ values are presented for compounds **16** (A), **11** (B), **12** (C), and FPP (D). *IC*_50_ values were determined by nonlinear regression to a three‐parameter dose–response equation (Eqn 2). All replicate data points were used for curve fitting. The goodness‐of‐fit values (*R* ^2^) were 0.9845, 0.9744, 0.9773, and 0.9737 for (A), (B), (C), and (D), respectively. Displayed data points represent mean ± standard deviation (*n* = 3). The *x*‐axes are in logarithmic scale.

**Fig. 7:**
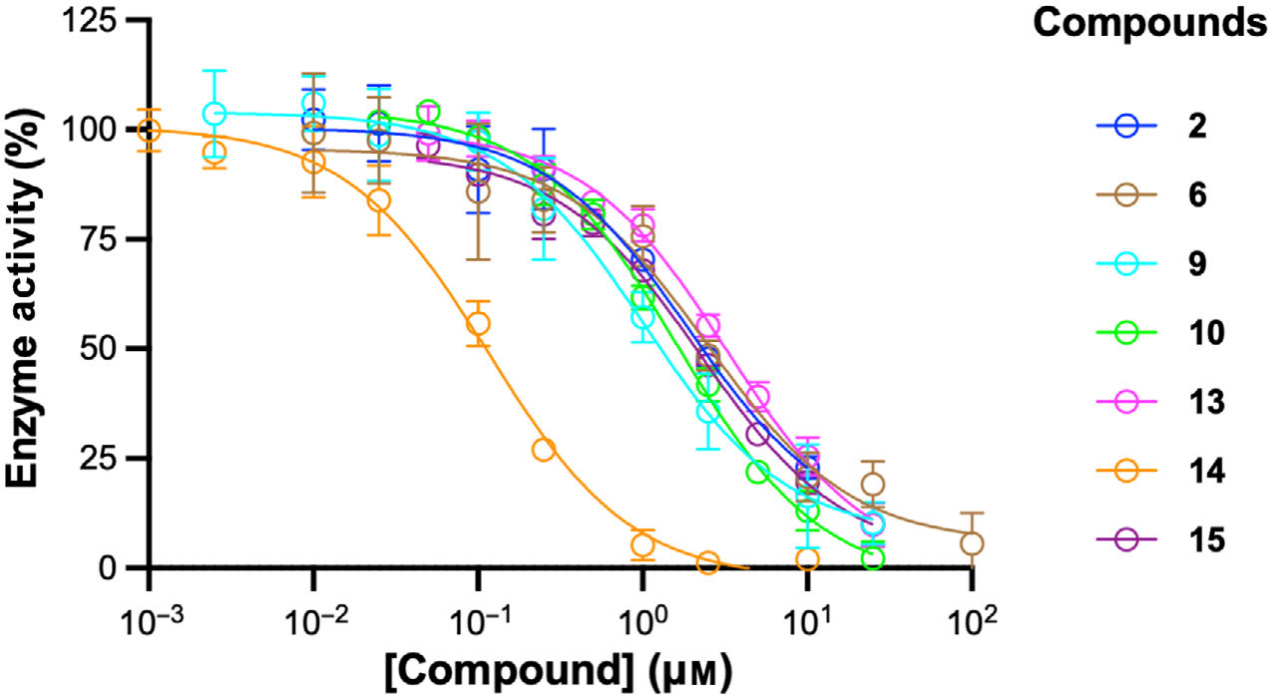
Dose–response curves for other MK inhibitors. All replicate data points were used for curve fitting. The goodness‐of‐fit values (*R* ^2^) were 0.9586, 0.9422, 0.9565, 0.9928, 0.9875, 0.9871, and 0.9880 for compounds **2**, **6**, **9**, **10**, **13**, **14**, and **15**, respectively. Displayed data points represent mean ± standard deviation (*n* = 3). The *x*‐axis is in logarithmic scale.

To accurately assess the potency of the inhibitors, we determined their *IC*_50_ values. Ten compounds, which exhibited > 60% inhibition at 10 μm concentration in the previous screening study, were evaluated. The most potent inhibitor was **16**, with an *IC*_50_ of 8.1 nm (Fig. 6A). Following closely were **11** and **12**, with an *IC*_50_ of 42 and 46 nm, respectively (Fig. 6B, C). In comparison, FPP showed an *IC*_50_ of 12 nm (Fig. 6D). The *IC*_50_ values for the remaining compounds ranged from 120 nm to 3.3 μm (dose–response curves are presented in Fig. 7). The determined *IC*_50_ values for all compounds are listed in Table 3.

**Table 3:** *IC*_50_ of phosphonate inhibitors and FPP for MK inhibition.

Compound	*IC*_50_ (μm)
**2**	1.9
**6**	2.5
**9**	1.0
**10**	1.6
**11**	0.042
**12**	0.046
**13**	3.3
**14**	0.12
**15**	2.3
**16**	0.0081
FPP	0.012

### Mode of inhibition by MK inhibitors

Subsequently, we conducted kinetic analysis to elucidate the mode of inhibition by the MK inhibitors. Given that FPP inhibits MK by competing against ATP [18, 19, 20], we expected these inhibitors to have a similar inhibition mechanism. We examined the two most potent inhibitors, **16** and **11**. As a close analogue, **12** was presumed to behave similarly to **11**.

**Fig. 8:**
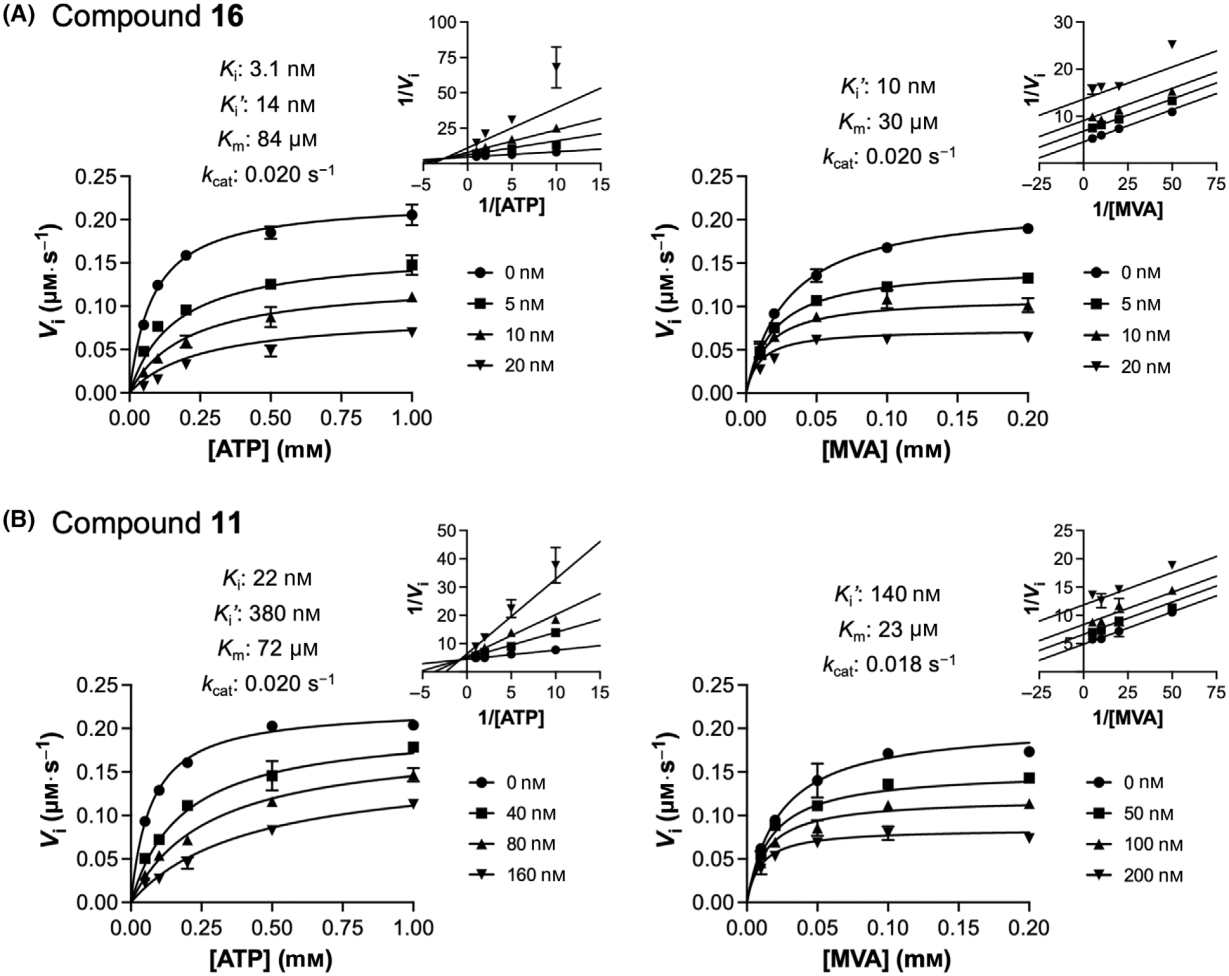
Kinetic analysis of MK inhibition. Effects of compounds **16** (A) and **11** (B) on the MK reaction. Initial rates were measured under saturating conditions of MVA (left panels) and ATP (right panels). Kinetic parameters were determined by global nonlinear regression to the mixed inhibition model (Eqn 6) with respect to ATP (left panels) and the uncompetitive inhibition model (Eqn 5) with respect to MVA (right panels). All replicate data points were used for curve fitting. The goodness‐of‐fit values (*R* ^2^) were 0.9784 (left panel) and 0.9789 (right panel) for compound **16** (A), and 0.9895 (left panel) and 0.9718 (right panel) for compound **11** (B). Displayed data points represent mean ± standard deviation (*n* = 2–4). Small plots display the double‐reciprocal representations of the rate data and fitted curves.

The kinetic data obtained with ATP as a varied substrate were best fitted to the mixed inhibition model (left panels, Fig. 8A,B), a general model encompassing all three types of reversible inhibition as special cases. Here, the ratio of the binding constants *K*_i_′/*K*_i_ informs about the mode of inhibition. Importantly, for both inhibitors, the binding constant was substantially smaller for the free enzyme than for the ATP‐bound form (i.e., *K*_i_ < *K*_i_′). This observation indicates that the inhibitors indeed have a competitive relationship with ATP. The difference between *K*_i_ and *K*_i_′ is more pronounced for **11**, with a *K*_i_′/*K*_i_ ratio of ~ 17. At this value, the mode of inhibition effectively approaches pure competitive inhibition, as evidenced by the linearized rate curves intersecting near the Y‐axis (double‐reciprocal plot, Fig. 8B left panel).

In contrast, under ATP‐saturating conditions, both inhibitors produced parallel lines in the double‐reciprocal plot (right panels, Fig. 8A,B), demonstrating an uncompetitive relationship with respect to MVA. This result suggests that the inhibitors bind more tightly to MK in the presence of MVA, consistent with the notion that they bind to the ATP binding site of the enzyme. Co‐binding of MVA to an adjacent site likely stabilizes the enzyme‐inhibitor‐substrate ternary complex. While structural information on human MK‐ligand interactions is limited, FPP binding to the ATP site was previously confirmed in rat MK structures [20].

## Discussion

In this study, through a systematic exploration of compounds initially designed as allosteric inhibitors of FPPS, we identified MK inhibitors with nanomolar activity. The *K*_i_ values of the most potent inhibitors were determined to be 3.1 and 22 nm. To the best of our knowledge, this is the first report of synthetic compounds that can inhibit human MK with nanomolar potency.

**Fig. 9:**
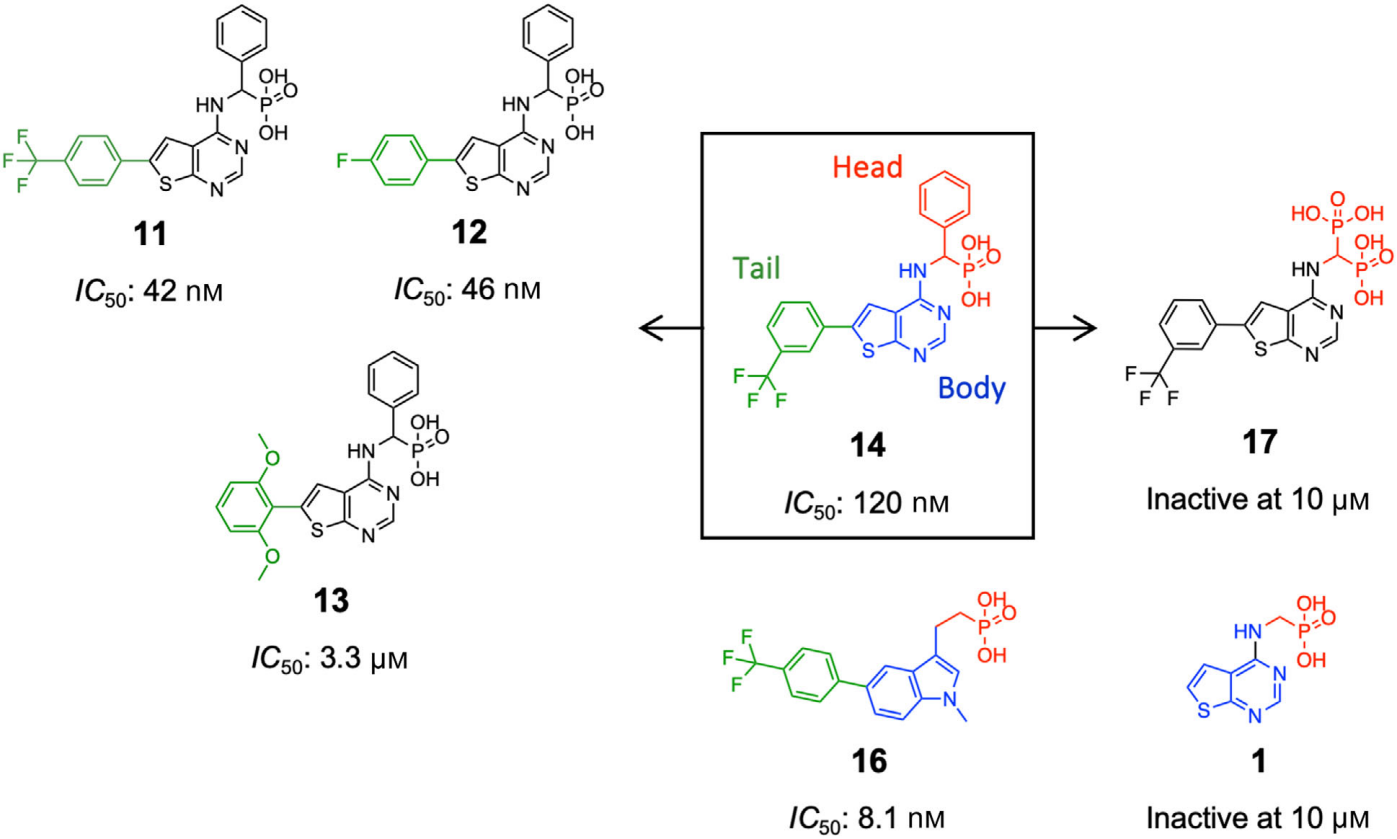
Structural determinants of MK inhibitor potency. Chemical structures of representative compounds are displayed, with relevant functional groups indicated in different colors.

The comparison of MK inhibitors suggests that the key determinants of their potency primarily reside in two structural components: the head and tail groups (inset, Fig. 9). As a head group, benzylphosphonate (e.g., **14**) and plain monophosphonate (e.g., **16**) contribute to the greatest inhibitory potency (Fig. 9). Under physiological pH, these groups become deprotonated and negatively charged, thus able to mimic the pyrophosphate of FPP. This mimicry likely facilitates the binding of the inhibitors to the ATP site of MK. The rat MK structures mentioned earlier indicate that a coordinated Mg^2+^ ion mediates the binding of negatively charged ligands at this site, as observed with both ATP and FPP [20]. In contrast, the bisphosphonate moiety in our compounds is much less effective as a head group (e.g., **14** vs. **17**, Fig. 9). The main scaffold, or the ‘body’, of our inhibitor compounds comprises a two‐heterocyclic bioisostere: the thienopyrimidine core (e.g., **14**) or methylindole core (e.g., **16**) (Fig. 9). Assessing the specific contributions of these scaffolds to inhibitory potency would necessitate the evaluation of many more compounds from both subclasses. Nevertheless, the tail group attached to the scaffold evidently impacts potency. Based on this preliminary study, the presence of a 6‐aryl‐substitution is required for potent inhibitors, whereas the absence of a tail group results in an inactive compound (e.g., **1**, Fig. 9). Functional groups on the aryl ring also lead to significant differences in potency, with variations of up to ~ 80‐fold (e.g., **11**/**12** vs. **13** vs. **14**, Fig. 9).

Although not formally part of this report, we also conducted virtual docking trials to gain further insight into the structure–function relationship of our inhibitors. A particular challenge was the lack of experimental structures with bound ligands for human MK. The only available structure is that of the apo‐enzyme form, unliganded to any small molecules or metal ions (PDB ID: 2R3V). Since a metal ion is presumably required for the binding of our inhibitors, we modeled a magnesium ion based on a rat MK structure (PDB ID: 1KVK). However, despite our best efforts using different docking engines, we did not observe any significant correlation between the predicted binding energy and the experimentally determined potency of the test compounds. Importantly, in the absence of experimental structures with bound ligands, control experiments through retrospective docking were not feasible. Such experiments would have been useful for increasing confidence in the accuracy of predicted binding poses and energies.

It is noteworthy that the MK inhibitors identified in this study are relatively weak inhibitors of FPPS, the original target for which these compounds were synthesized. For example, the two most potent MK inhibitors, **16** and **11**, exhibited < 50% inhibition of FPPS at 10 μm concentration (unpublished data). The most potent inhibitor of FPPS among the tested compounds, **5**, showed an *IC*_50_ of 1.1 μm [30]. The micromolar potency of these compounds for FPPS aligns well with the micromolar binding affinity of FPP for FPPS (*K*_d_ = 5–6 μm) [21]. In comparison, the binding affinity of FPP for MK is in the low nanomolar range (*K*_i_ = 10 and 34 nm, two independent studies) [19, 20]. Therefore, it makes sense that, acting as FPP mimetics, monophosphonate compounds can inhibit MK in the same nanomolar range. While a direct comparison of potency against different enzymes is difficult, based on the available inhibition data, the most potent MK inhibitors inhibit MK 2–3 orders of magnitude more potently than FPPS.

As a result of the potent pharmacological effects produced by its modulation, the mevalonate pathway continues to attract significant interest in drug discovery. Beyond FPPS, other enzymes in the pathway, such as geranylgeranyl pyrophosphate synthase [43, 44] and phosphomevalonate kinase [45, 46], have been proposed as potential anticancer targets. Our discovery of potent and specific human MK inhibitors is exciting in this context, creating opportunities for future investigations. These inhibitors may prove useful as molecular probes to study mevalonate pathway regulation and explore the therapeutic potential of MK inhibition in model systems. They may even serve as new lead compounds in the development of drug candidates that can modulate the mevalonate pathway. In summary, the MK inhibitors identified in this study hold promise as valuable research tools and may contribute to furthering our understanding of the mevalonate pathway.

## Conflict of interest

The authors declare no conflict of interest.

### Peer review

The peer review history for this article is available at https://www.webofscience.com/api/gateway/wos/peer‐review/10.1002/2211‐5463.13853.

## Author contributions

JP conceived and oversaw the project; YST conceived and designed the synthesis of the library of potential prenylation inhibitors. JP designed the compound screening and enzyme kinetics experiments; SS produced the enzyme and acquired the inhibition and kinetics data; SS and JP analyzed and interpreted the data; HFL wrote the synthesis protocols for the test compounds and reanalyzed the compound characterization data; SS and JP wrote all other sections of the manuscript. We also wish to thank previous members of the Tsantrizos group, Chun Yuen Leung, Joris Wim De Schutter, Michail Tsakos, Wei Ling Chiu, and Yih‐Shyan Lin, for their initial contribution to the preparation of the compound library.

## Supporting information


**Data S1.** Compound characterization data.

## Data Availability

The data that support the findings of this study are available within the article and the supplementary material.
