# Supplementary material for: Inhibition of human mevalonate kinase by allosteric inhibitors of farnesyl pyrophosphate synthase
Source: FEBS Open Bio. 2024 Jun 23;14(8):1320–39. doi: 10.1002/2211-5463.13853 (PMC11301271; doi:10.1002/2211-5463.13853)

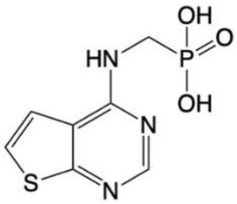

Compound 1

This compound has been previously reported (compound **7** in reference 30).

HPLC Chromatogram (**Compound 1**)

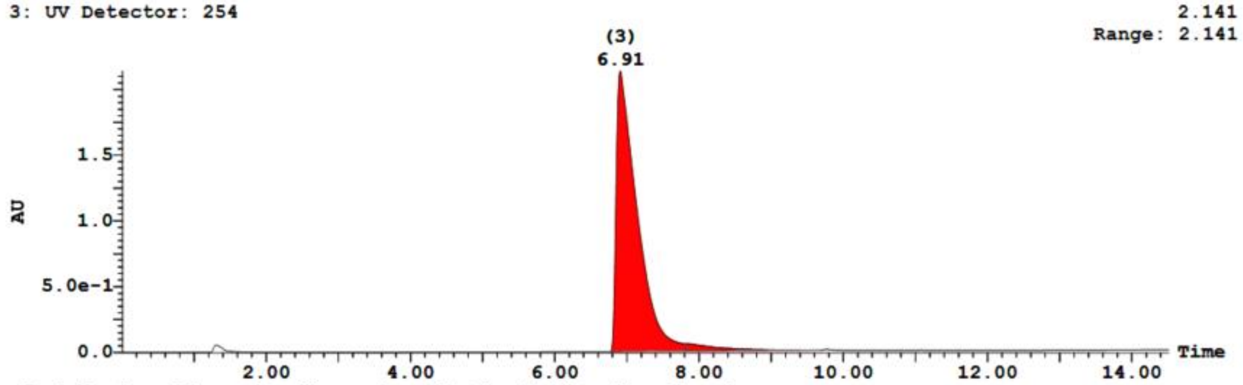

Peak Number	Time	AreaAbs	Area %Total	Height	Mass Found
3	6.91	715240	95.74	2136309	
4	7.85	31789	4.26	57357	

LRMS (**Compound 1**)

Peak ID	Time	Target
3	6.91	No Target

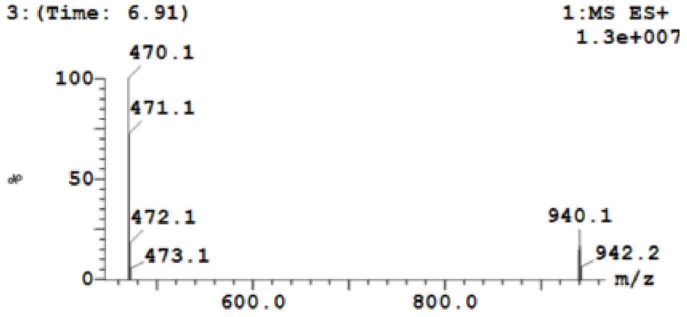

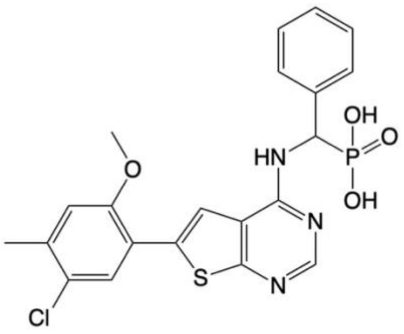

Compound 2

This compound has been previously reported (compound **24** in reference 30).

HPLC Chromatogram (**Compound 2**)

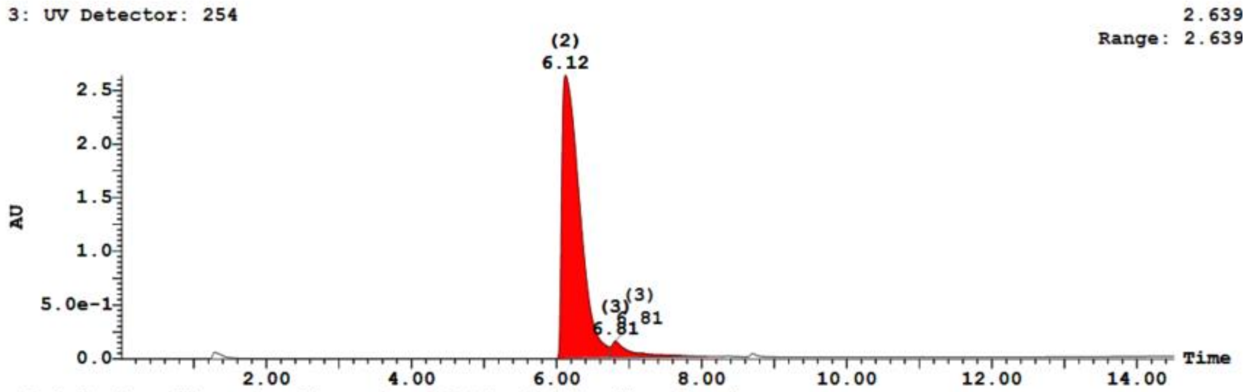

Peak Number	Time	AreaAbs	Area %Total	Height	Mass Found
2	6.12	757298	93.53	2635060	
3	6.81	35156	4.34	153402	
4	7.18	17203	2.12	39170	

LRMS (**Compound 2**)

Peak ID	Time	Target
2	6.13	No Target

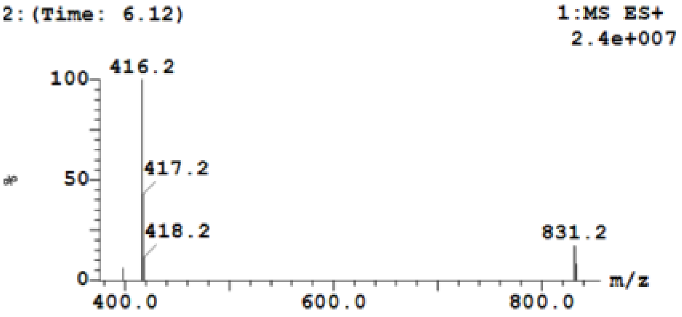

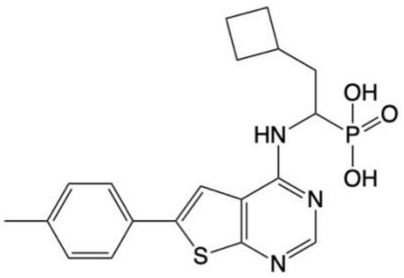

Compound 3

This compound has been previously reported (compound **36** in reference 30).

HPLC Chromatogram (**Compound 3**)

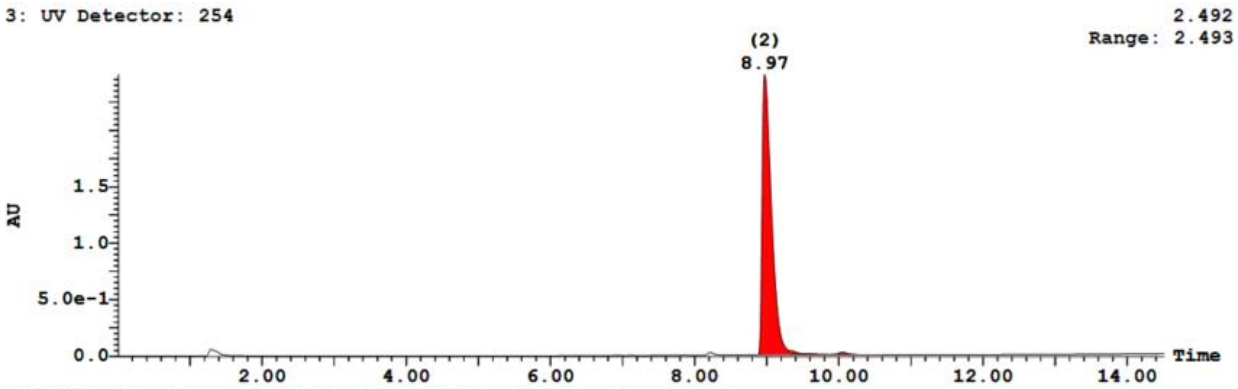

Peak Number	Time	AreaAbs	Area %Total	Height	Mass Found
2	8.97	371194	97.48	2483255	
3	9.35	5836	1.53	35559	
4	10.05	3776	0.99	21900	

LRMS (**Compound 3**)

Peak ID	Time	Target
2	8.98	No Target

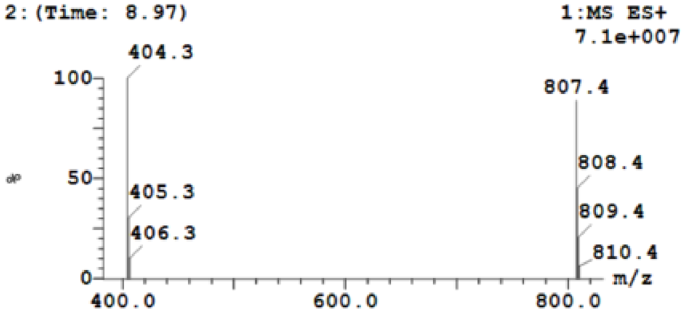

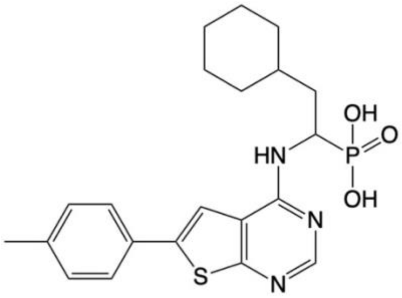

Compound 4

This compound has been previously reported (compound **37** in reference 30).

HPLC Chromatogram (**Compound 4**)

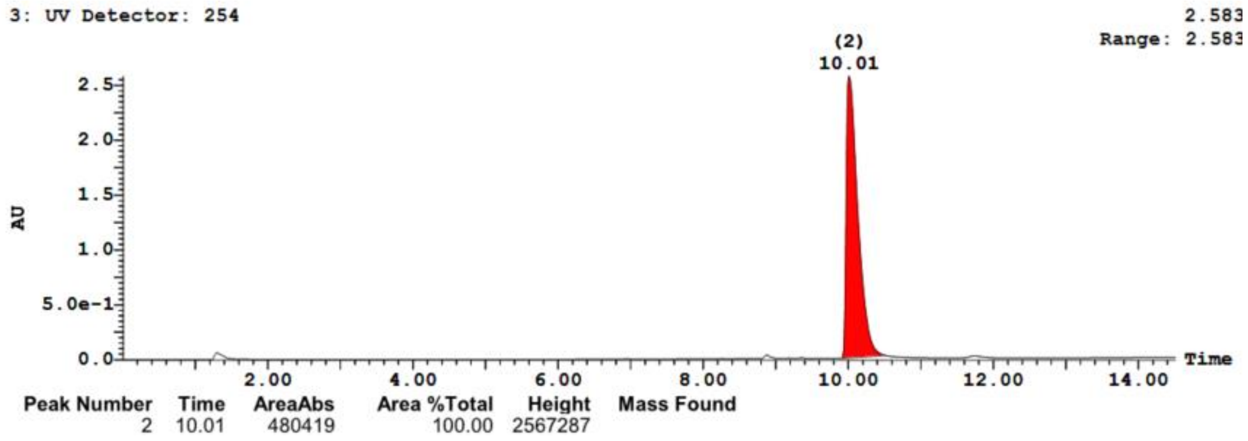

LRMS (**Compound 4**)

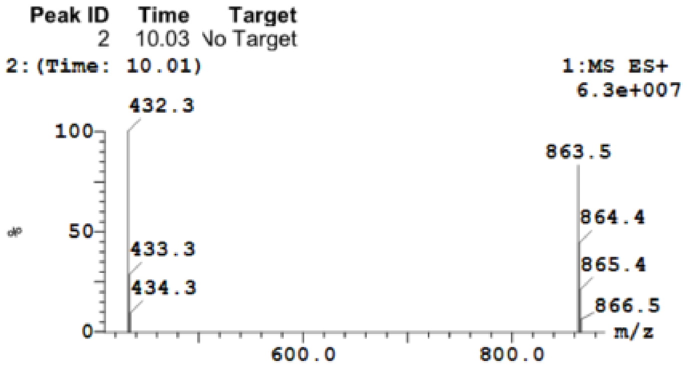

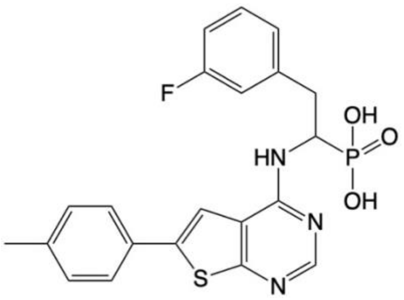

Compound 5

This compound has been previously reported (compound **38** in reference 30).

HPLC Chromatogram (**Compound 5**)

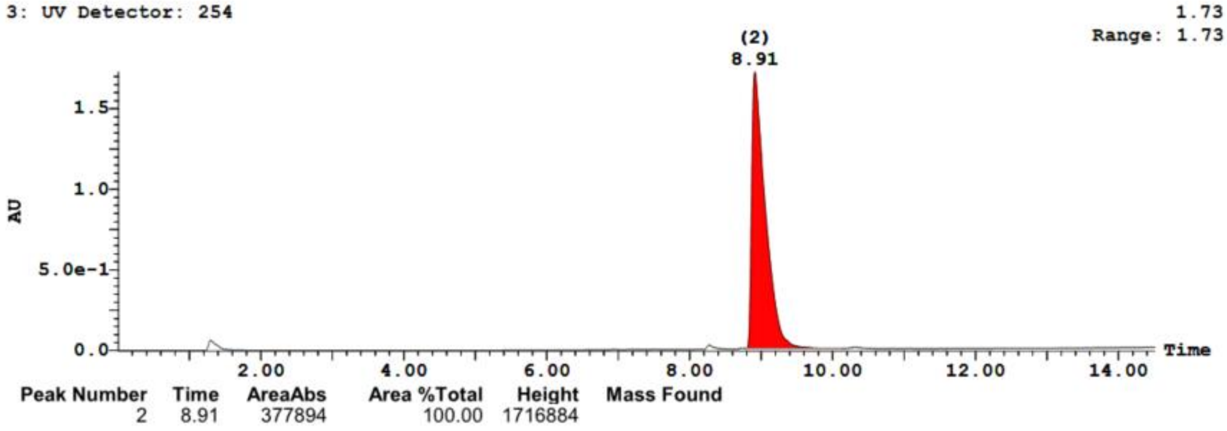

LRMS (**Compound 5**)

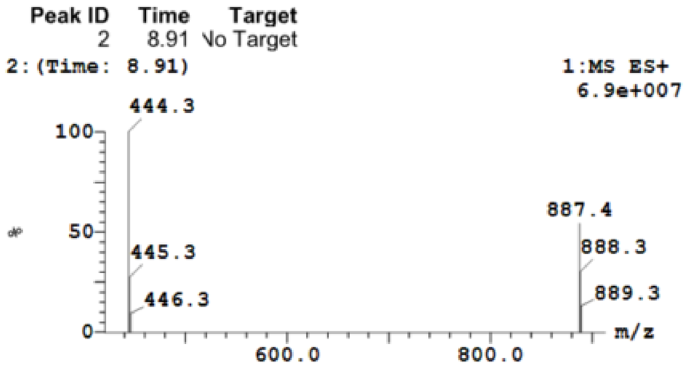

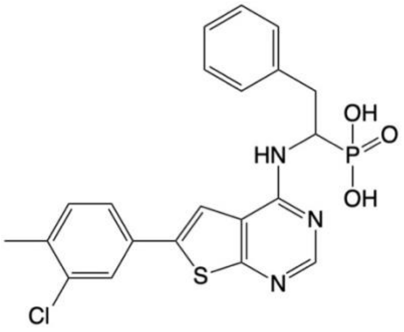

Compound 6

This compound has been previously reported (compound **20** in reference 30).

HPLC Chromatogram (**Compound 6**)

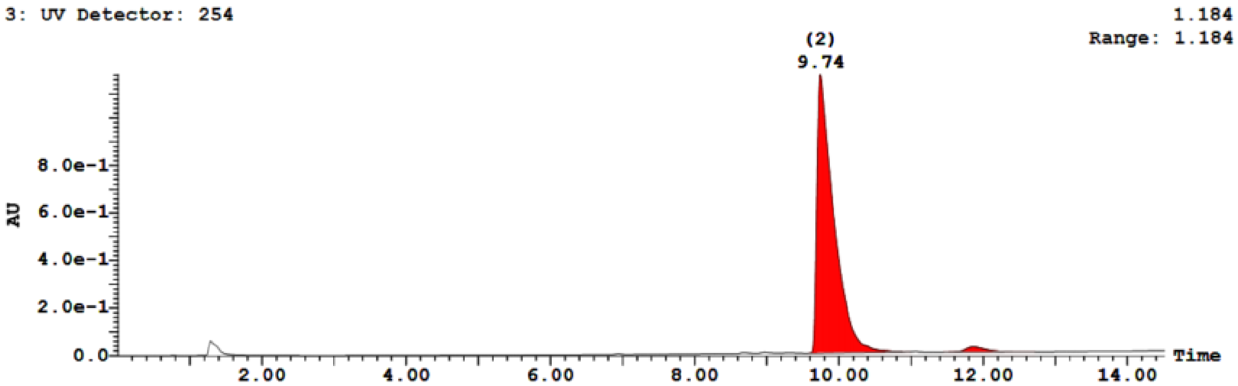

Peak Number	Time	AreaAbs	Area %Total	Height	Mass Found
2	9.74	320112	97.67	1173662	
4	11.86	7634	2.33	22145	

LRMS (**Compound 6**)

Peak ID	Time	Target
2	9.74	No Target

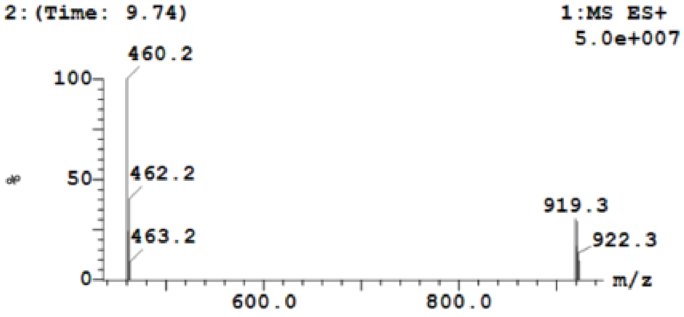

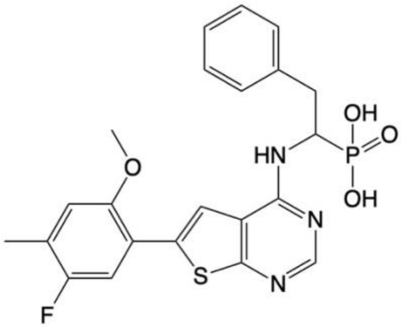

Compound 7

This compound has been previously reported (compound **22** in reference 30).

HPLC Chromatogram (**Compound 7**)

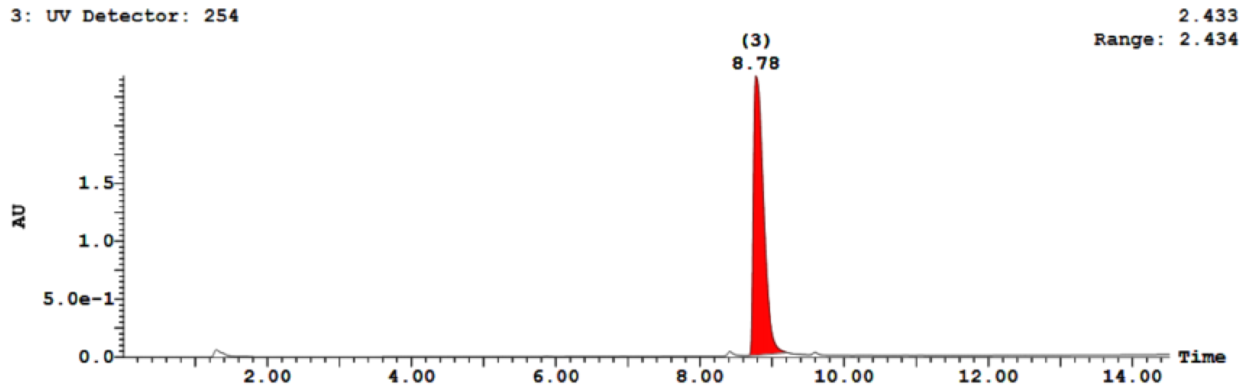

Peak Number	Time	AreaAbs	Area %Total	Height	Mass Found
3	8.78	400815	100.00	2418370	

LRMS (**Compound 7**)

Peak ID	Time	Target
3	8.81	No Target

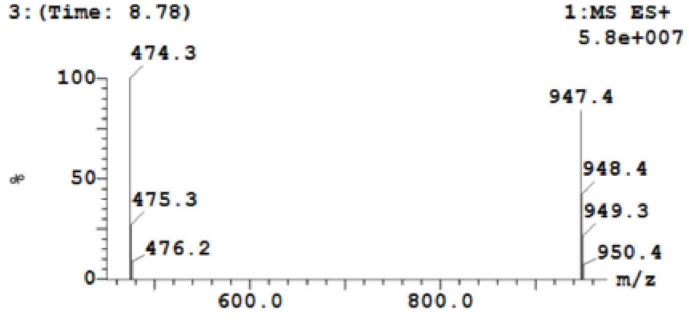

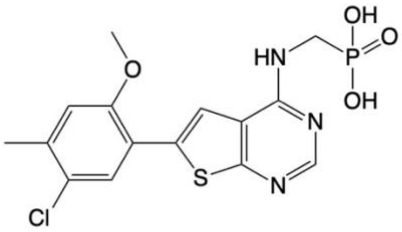

Compound 8

This compound has been previously reported (compound **21** in reference 30).

HPLC Chromatogram (**Compound 8**)

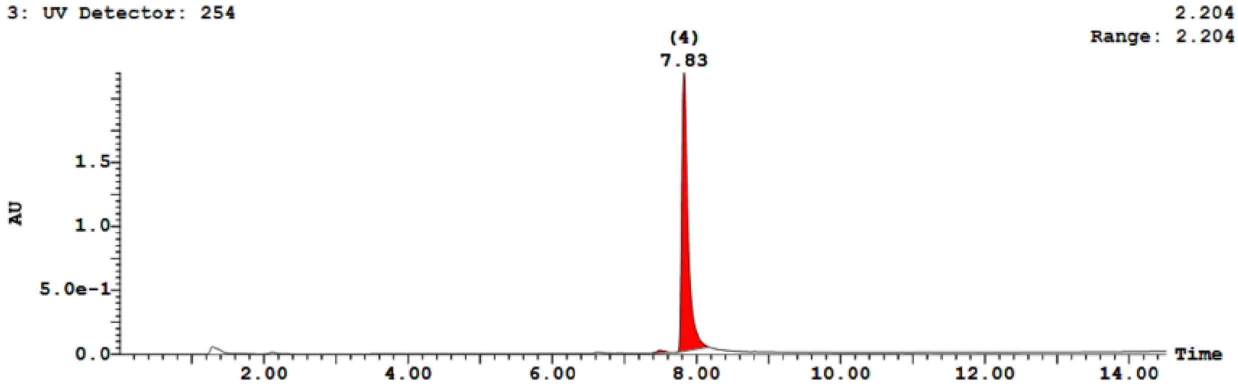

Peak Number	Time	AreaAbs	Area %Total	Height	Mass Found
3	7.49	2752	1.22	19159	
4	7.83	222416	98.78	2181558	

LRMS (**Compound 8**)

Peak ID	Time	Target
4	7.83	No Target

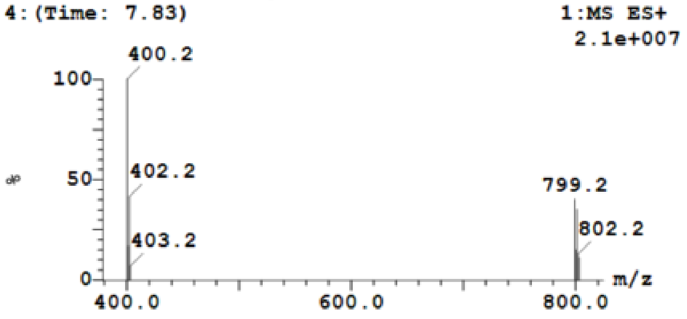

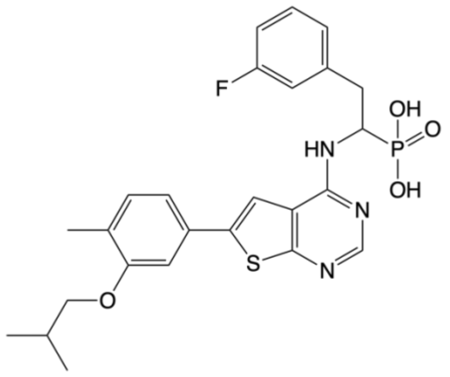

Compound 9

This compound has not been previously reported in the literature.

^{1}H NMR (500 MHz, DMSO-d$_6$) (**Compound 9**)

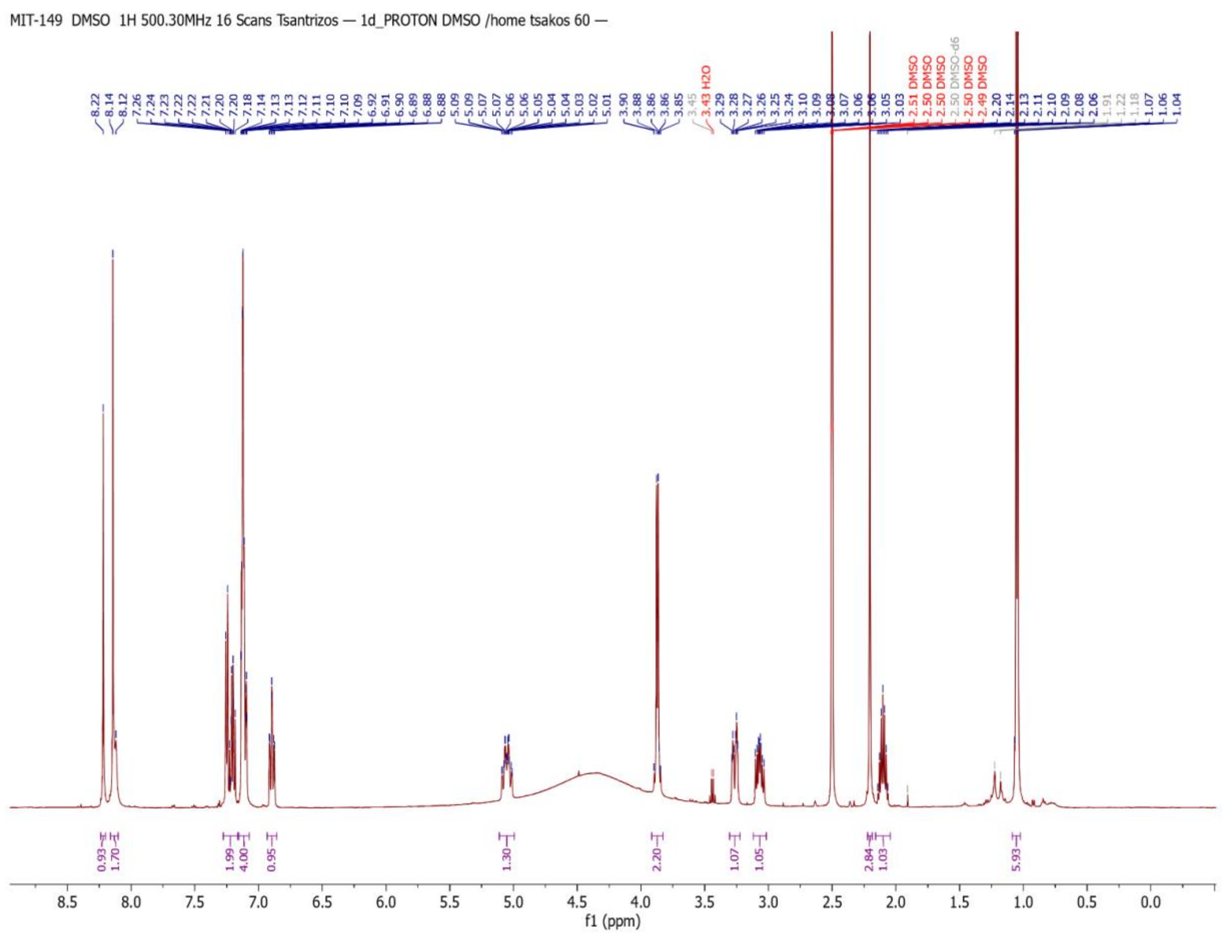

Expansion 1:

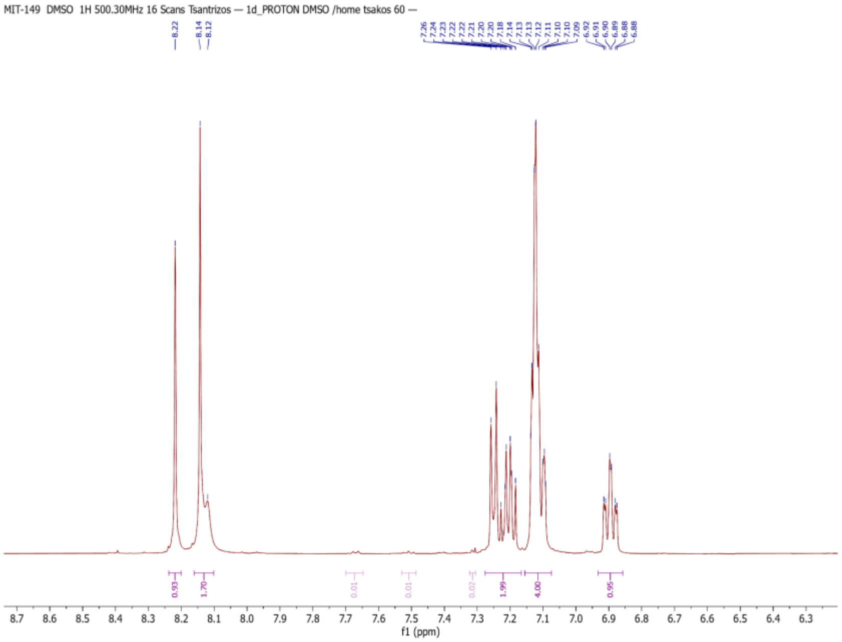

Expansion 2:

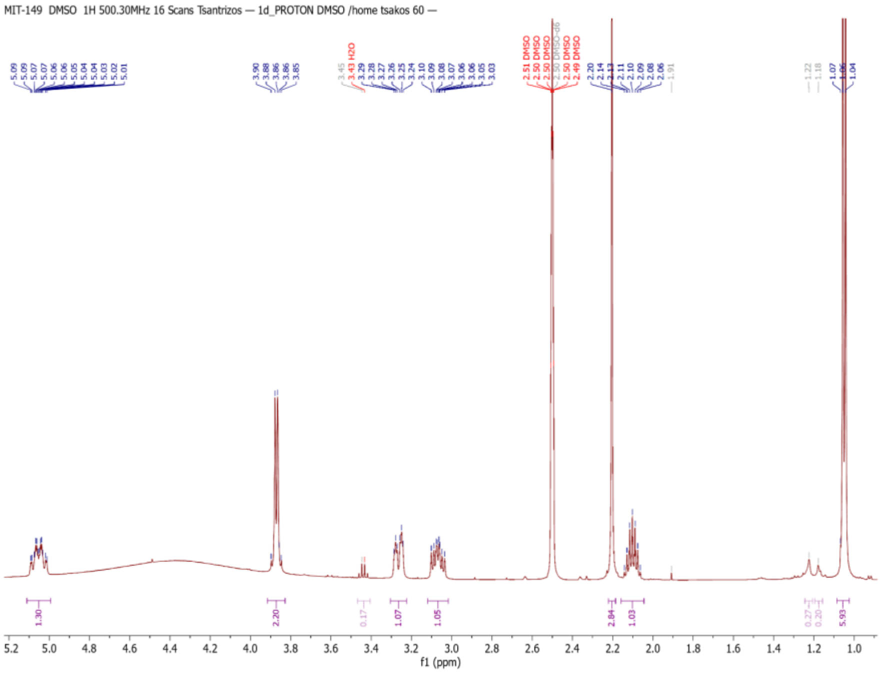

^{31}P NMR (203 MHz, DMSO-d$_6$) (**Compound 9**)

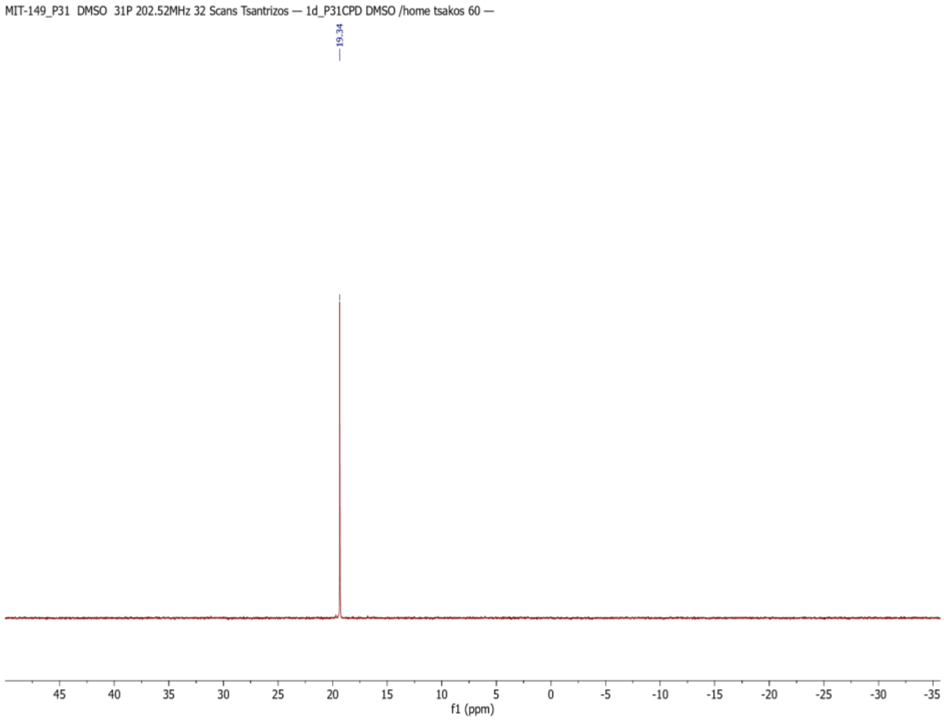

^{19}F NMR (471 MHz, DMSO-d$_6$) (**Compound 9**)

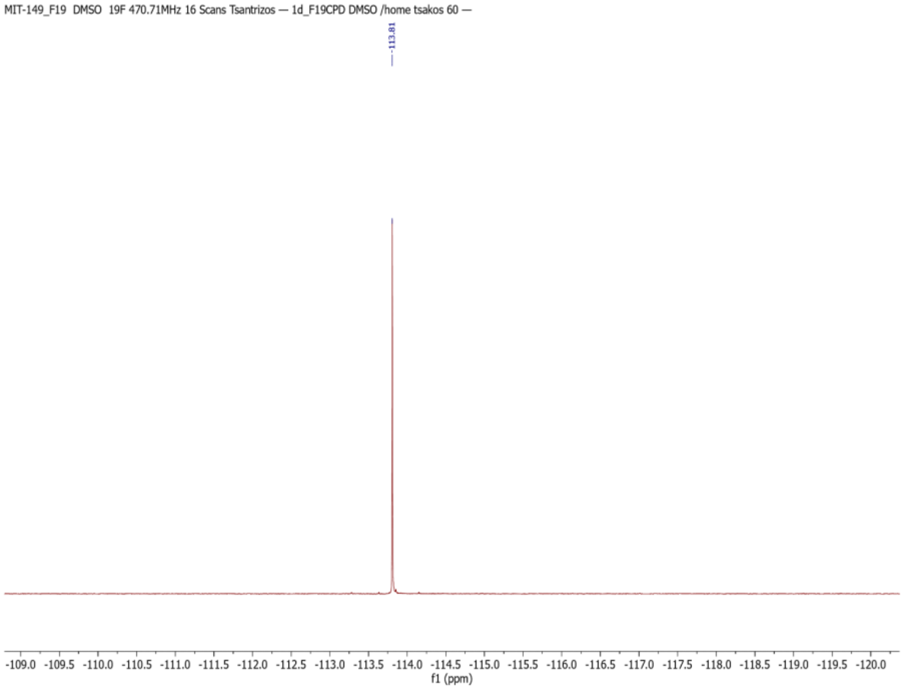

^{13}C NMR (126 MHz, DMSO-d_6) (**Compound 9**)

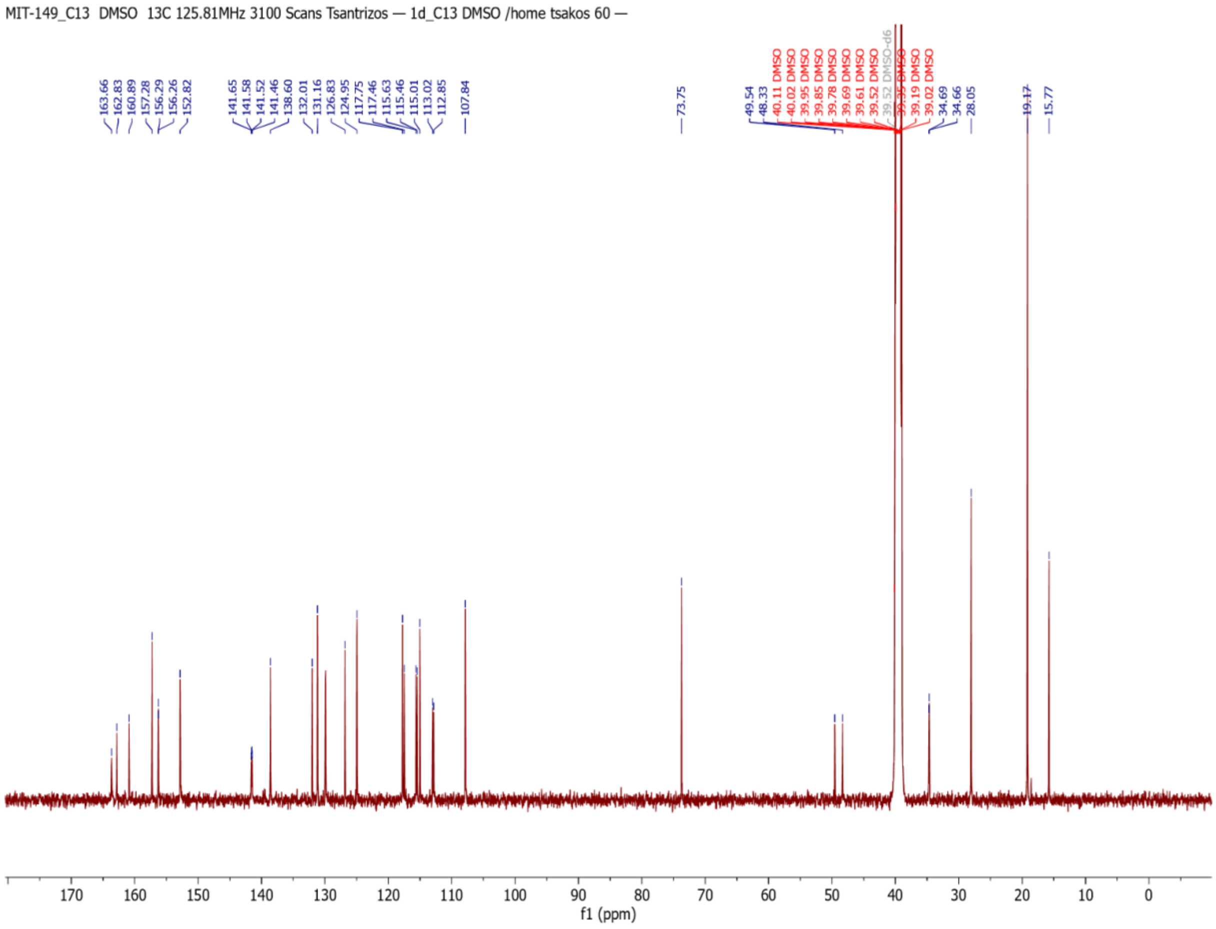

HPLC Chromatogram (**Compound 9**)

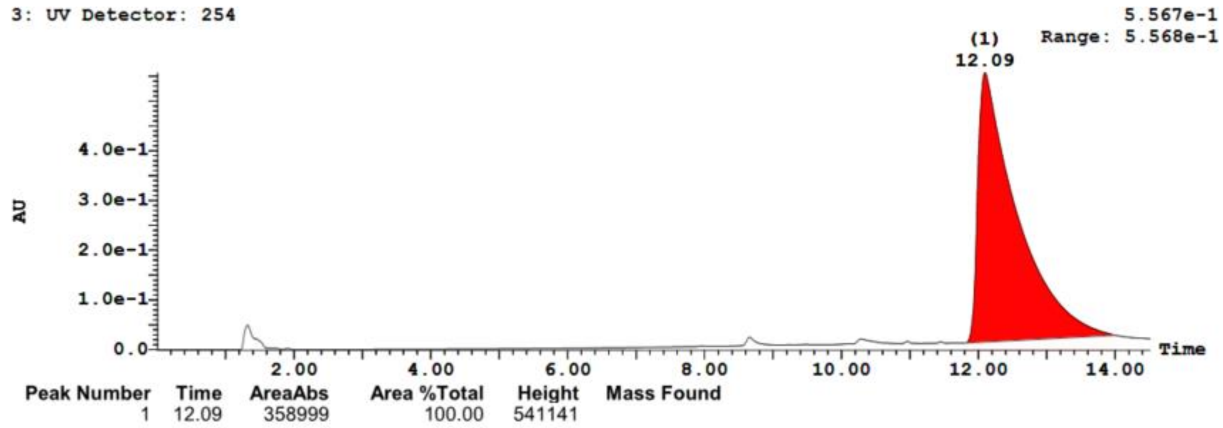

LRMS (**Compound 9**)

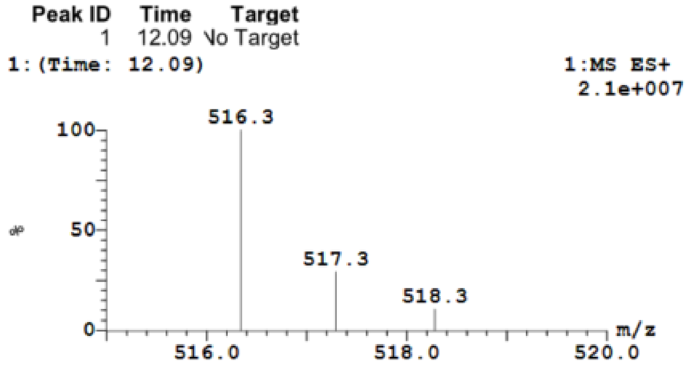

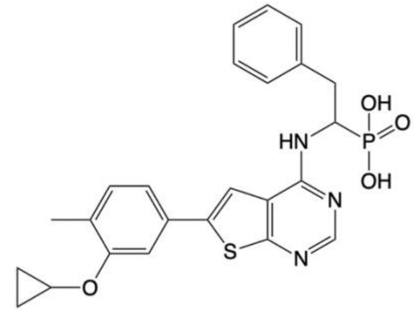

Compound 10

This compound has not been previously reported in the literature.

^{1}H NMR (500 MHz, DMSO-d_6) (**Compound 10**)

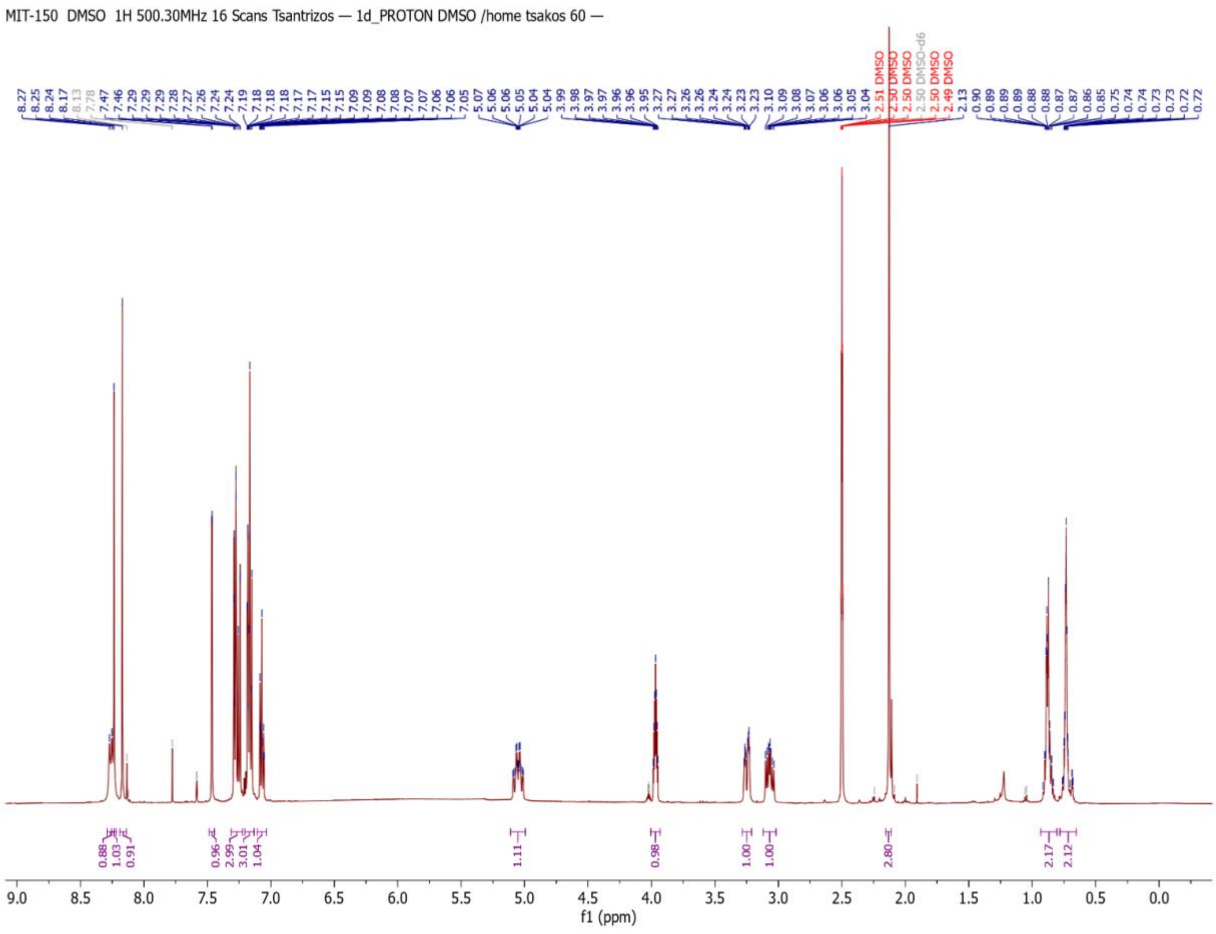

Expansion 1:

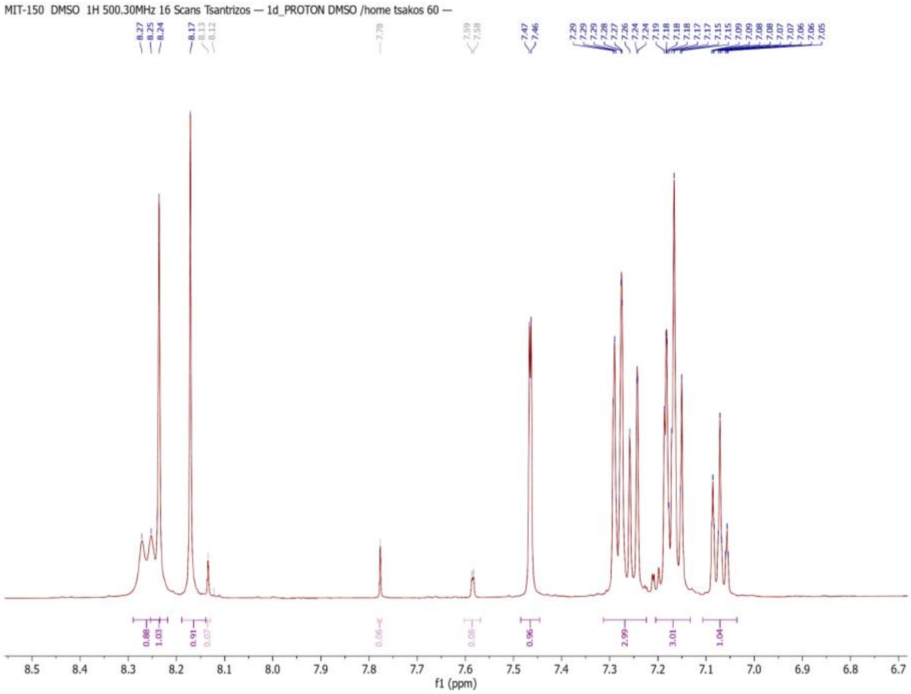

Expansion 2:

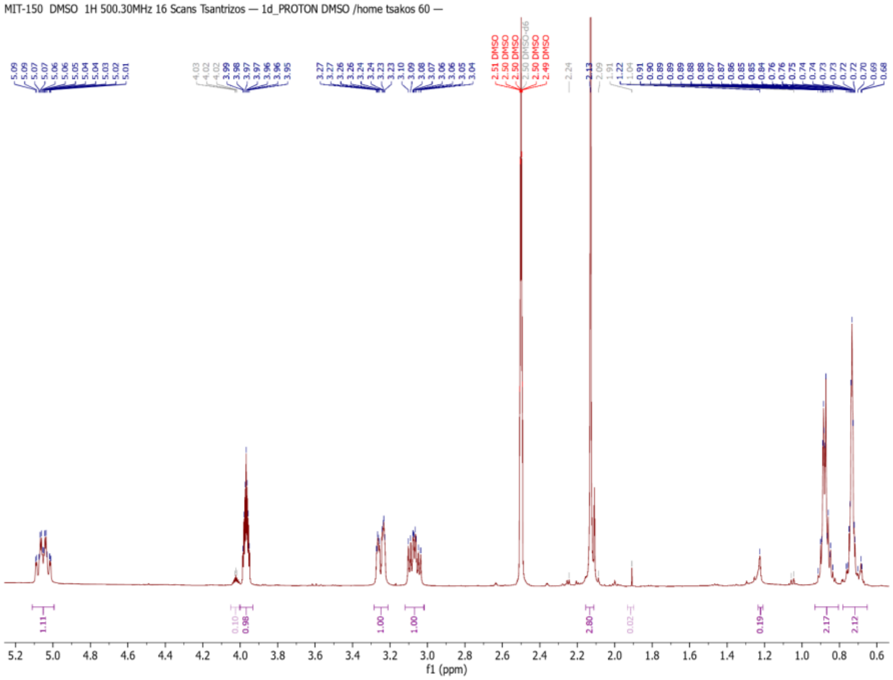

^{31}P NMR (203 MHz, DMSO-d_6) (**Compound 10**)

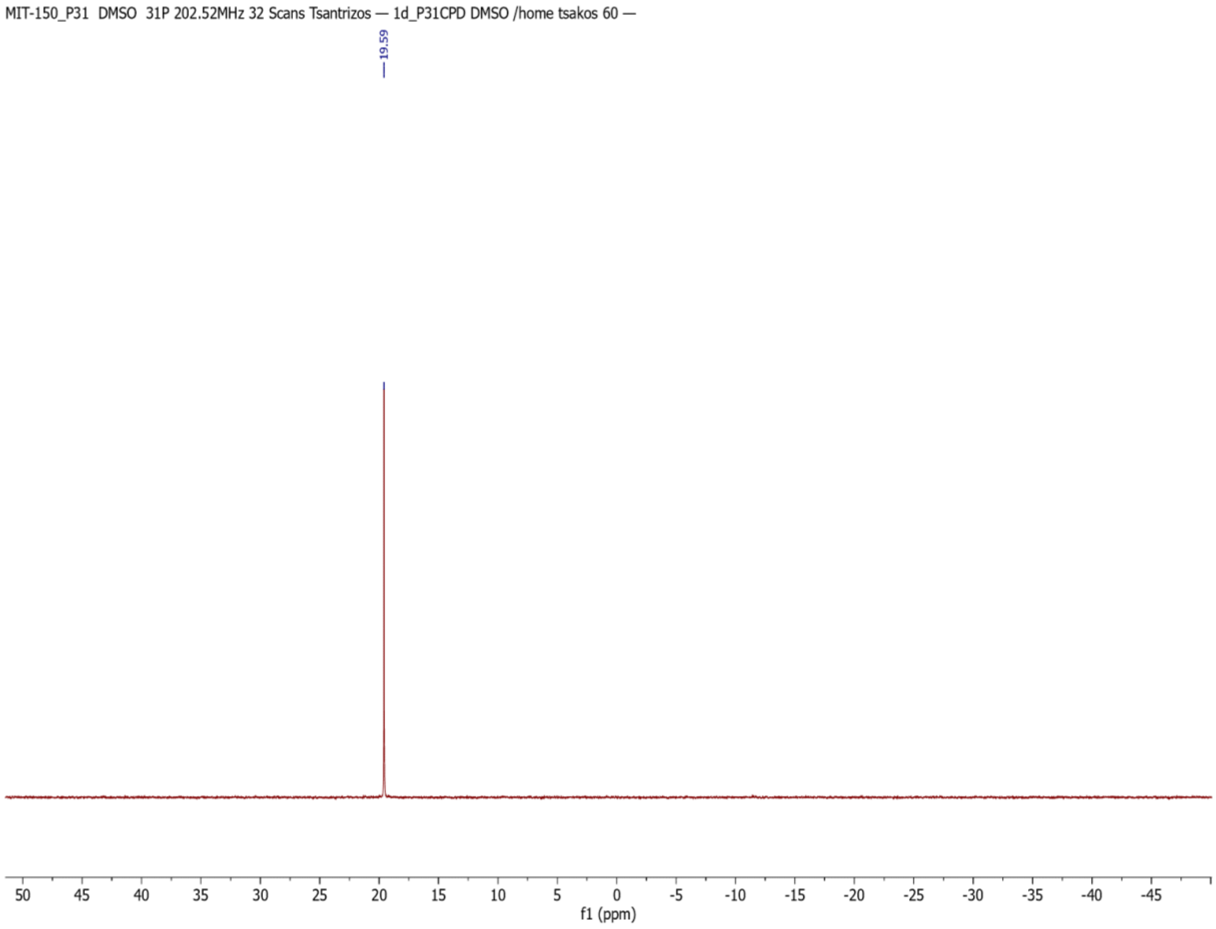

^{13}C NMR (126 MHz, DMSO-d6) (**Compound 10**)

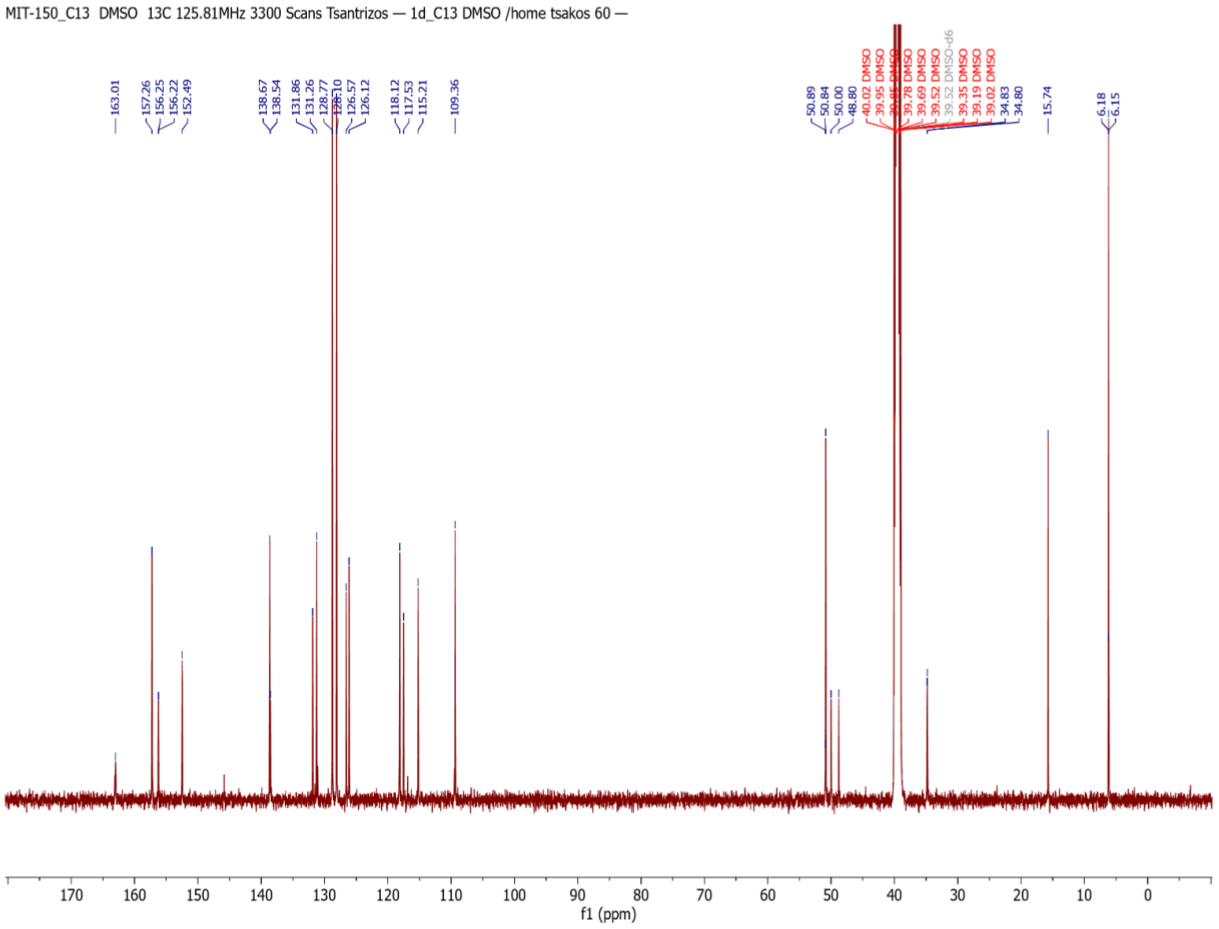

HPLC Chromatogram (**Compound 10**)

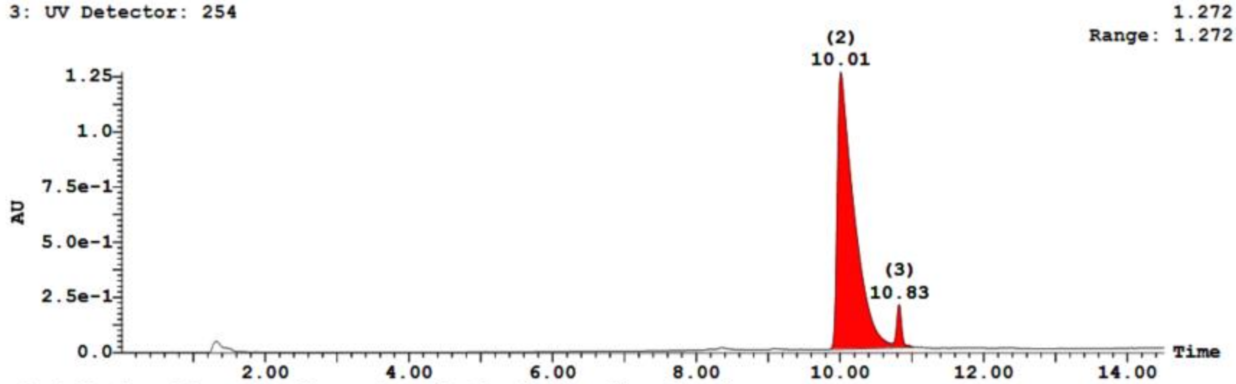

Peak Number	Time	AreaAbs	Area %Total	Height	Mass Found
2	10.01	342255	95.33	1257336	
3	10.83	16752	4.67	197008	

LRMS (**Compound 10**)

Peak ID	Time	Target
2	10.01	No Target

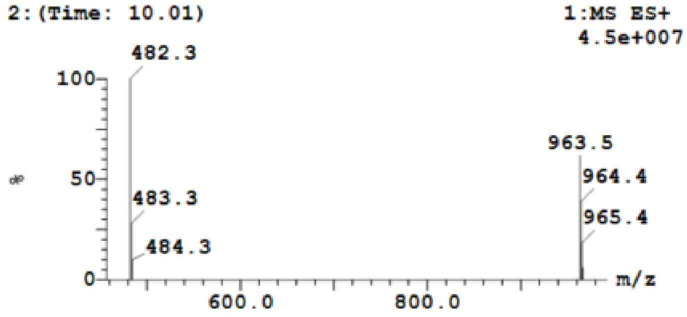

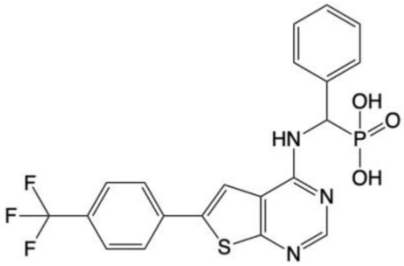

Compound 11

This compound has not been previously reported in the literature.

^{1}H NMR (800 MHz, 0.5% ND_4OD in D_2O) (**Compound 11**)

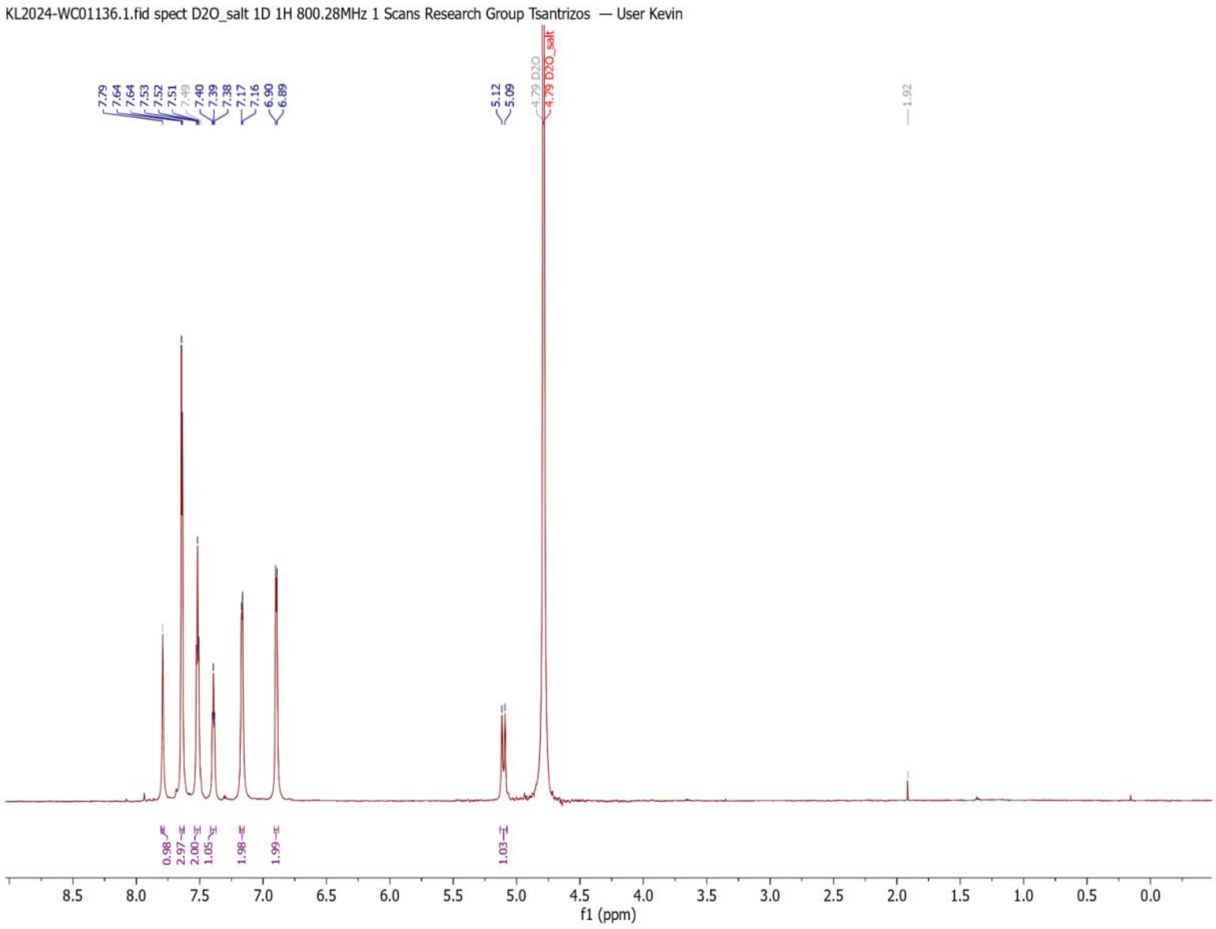

Expansion 1:

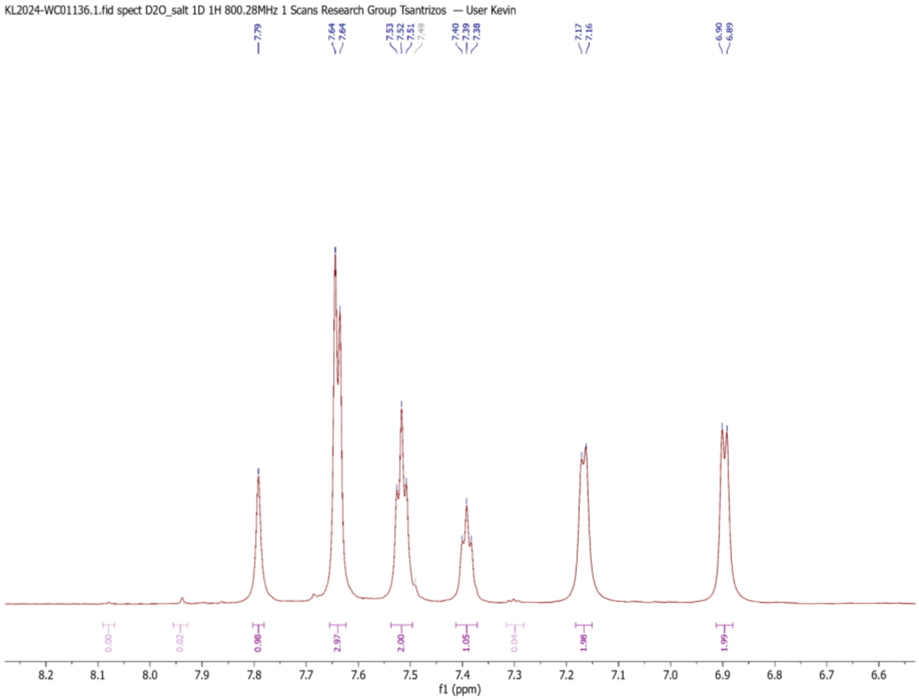

Expansion 2:

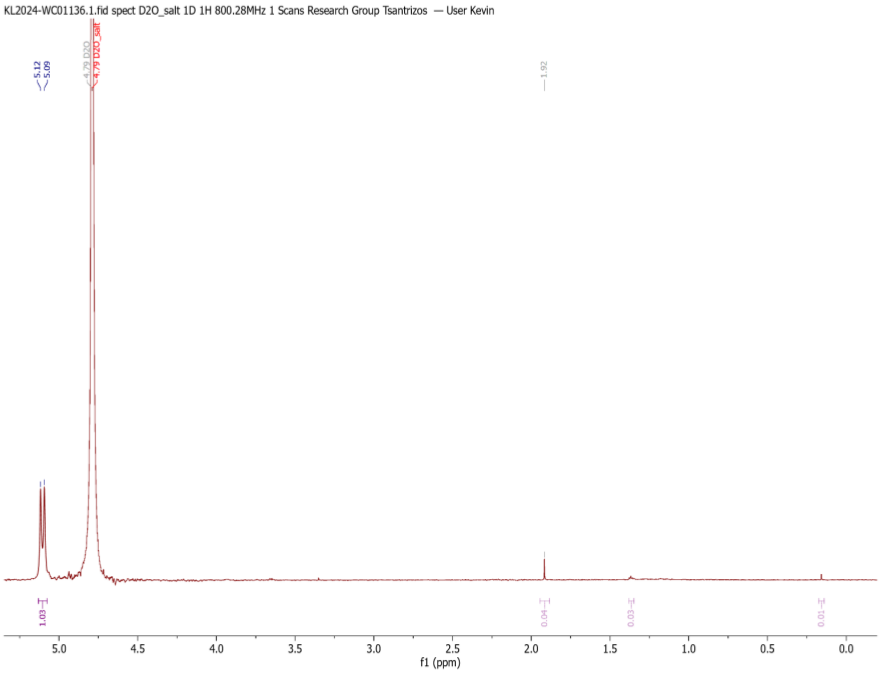

^{31}P NMR (203 MHz, 0.5% ND_4OD in D_2O) (**Compound 11**)

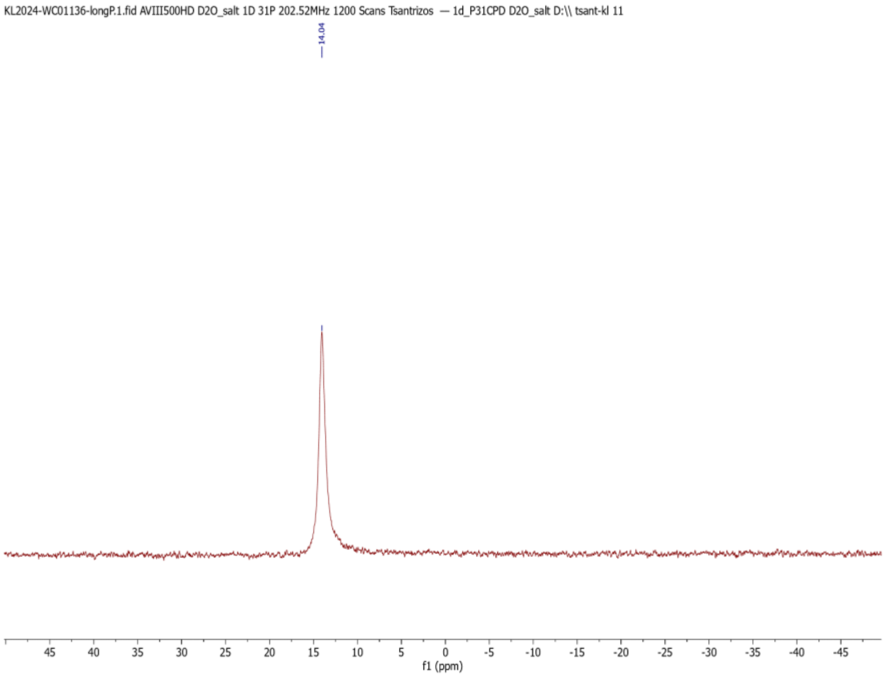

^{19}F NMR (470 MHz, 0.5% ND_4OD in D_2O) (**Compound 11**)

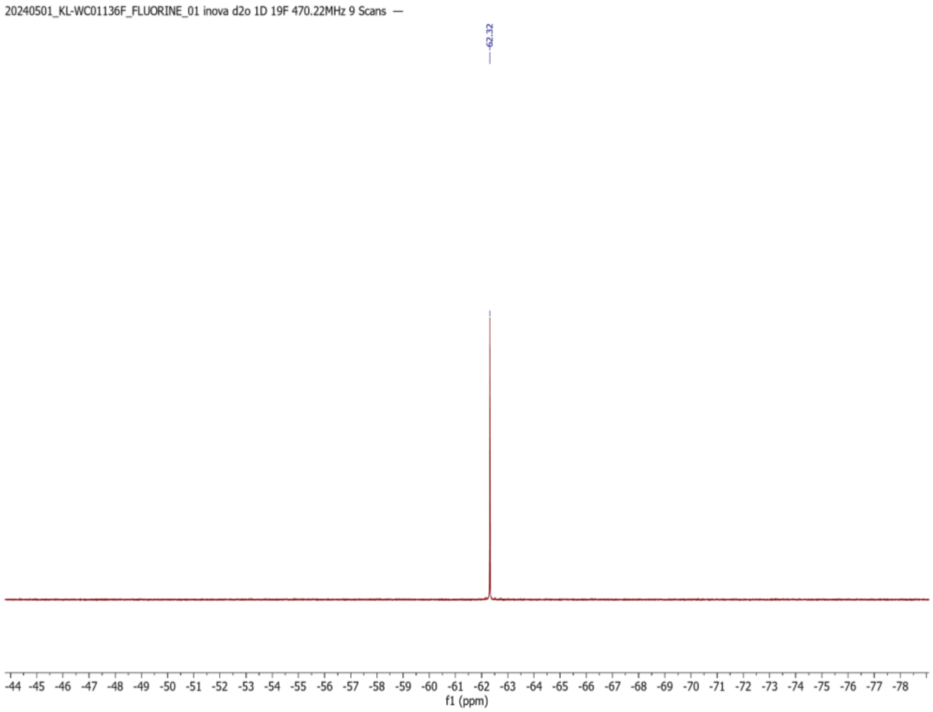

^{13}C NMR (201 MHz, 0.5% ND_4OD in D_2O) (**Compound 11**)

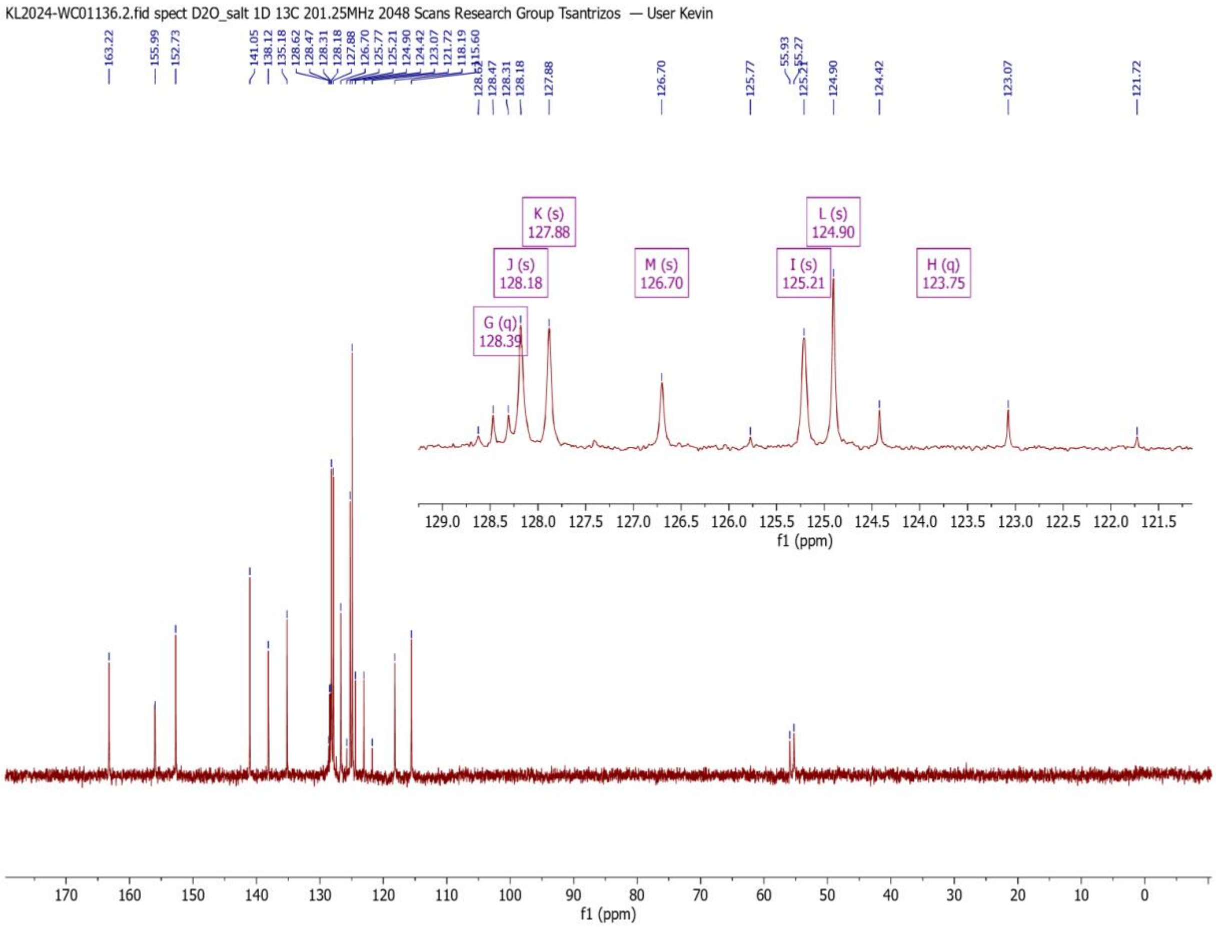

HPLC Chromatogram (**Compound 11**)

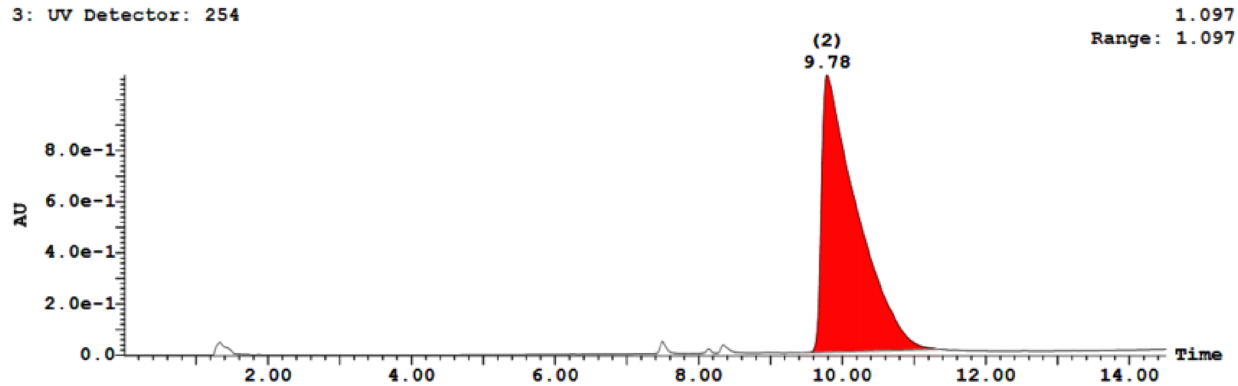

Peak Number	Time	AreaAbs	Area %Total	Height	Mass Found
2	9.78	624170	100.00	1084852	

LRMS (**Compound 11**)

Peak ID	Time	Target
2	9.78	No Target

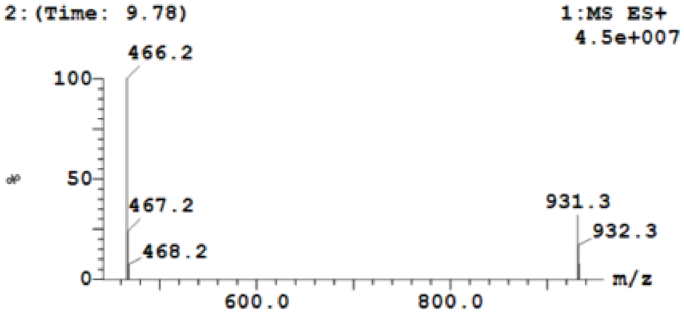

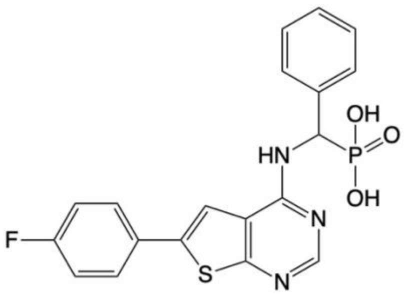

Compound 12

This compound has not been previously reported in the literature.

^{1}H NMR (800 MHz, 0.5% ND_4OD in D_2O) (**Compound 12**)

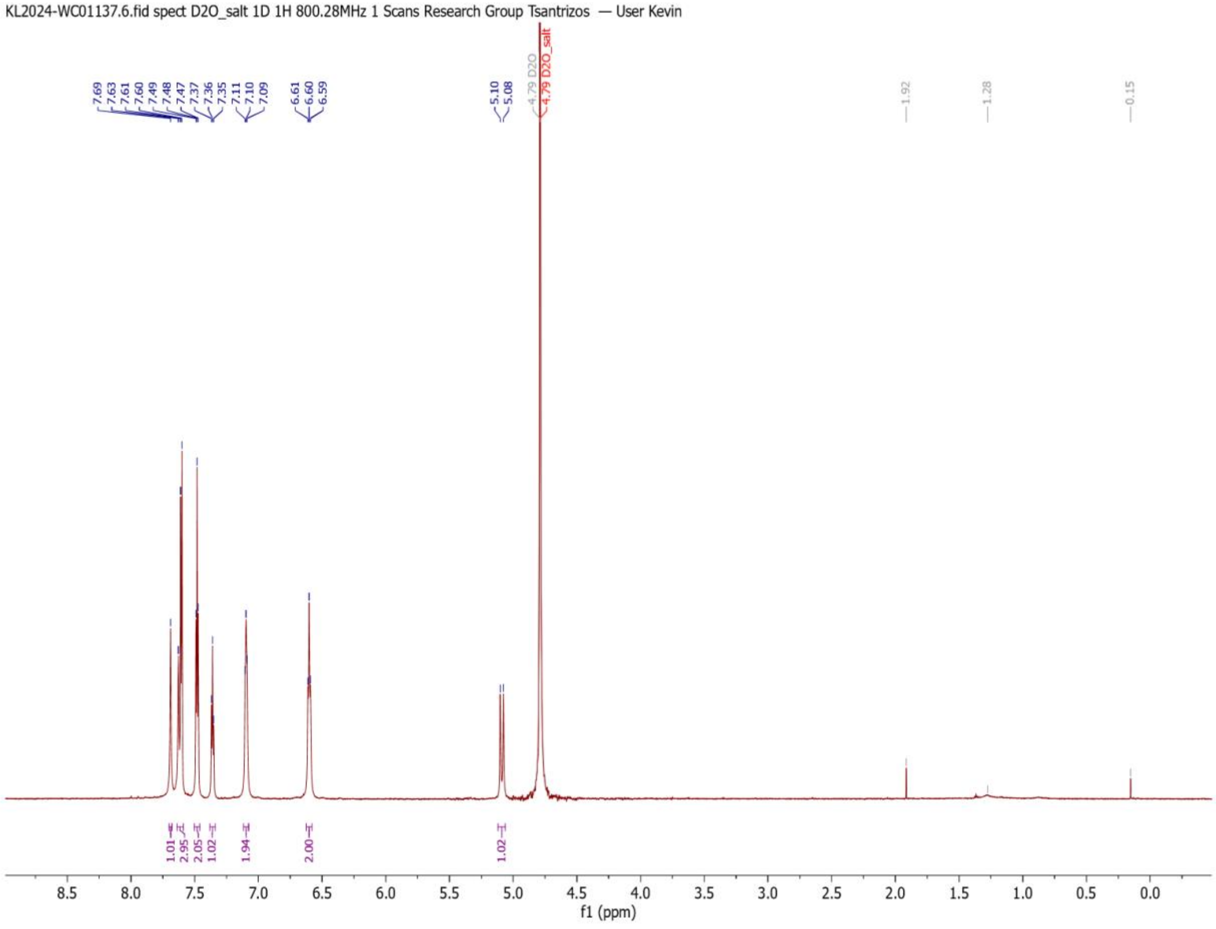

Expansion 1:

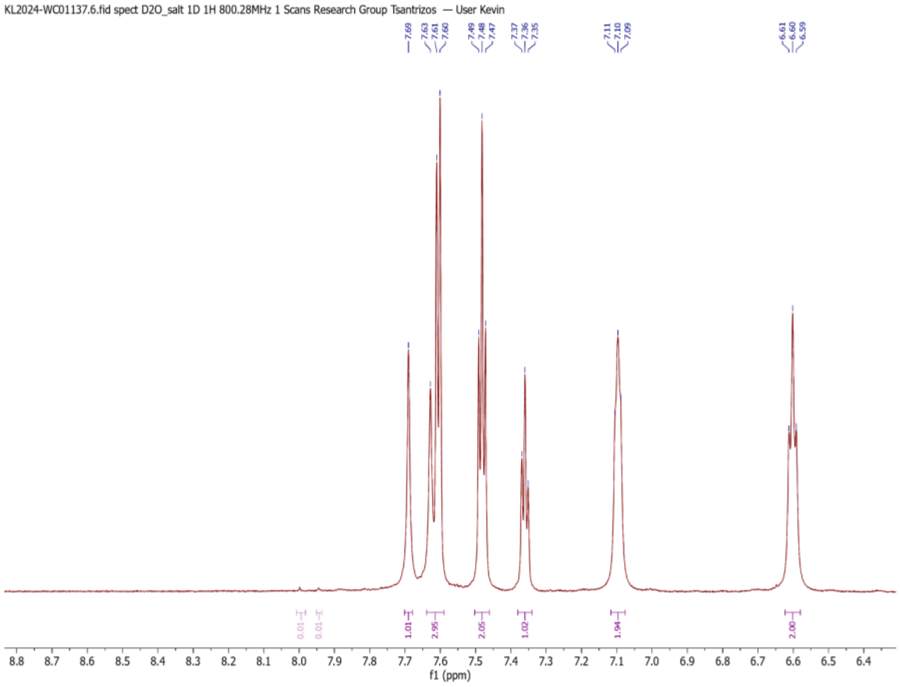

Expansion 2:

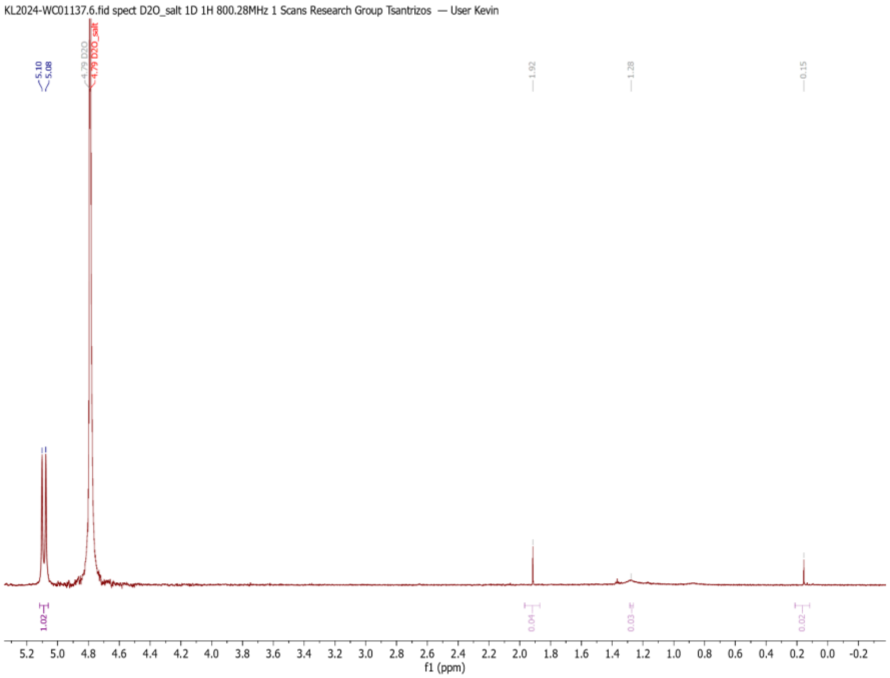

^{31}P NMR (203 MHz, 0.5% ND_4OD in D_2O) (**Compound 12**)

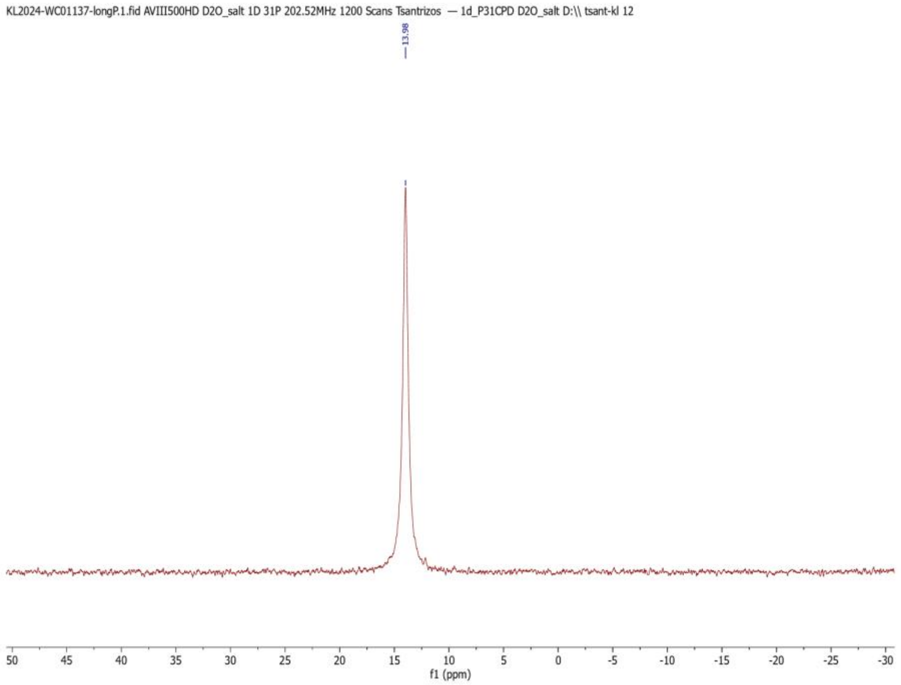

^{19}F NMR (470 MHz, 0.5% ND_4OD in D_2O) (**Compound 12**)

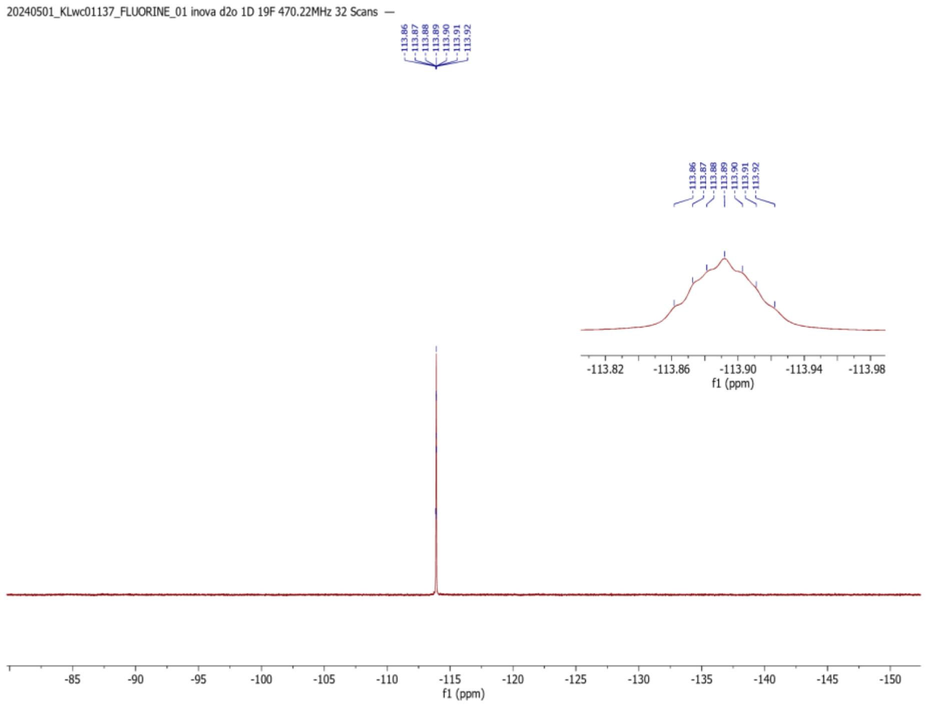

^{13}C NMR (201 MHz, 0.5% ND_4OD in D_2O) (**Compound 12**)

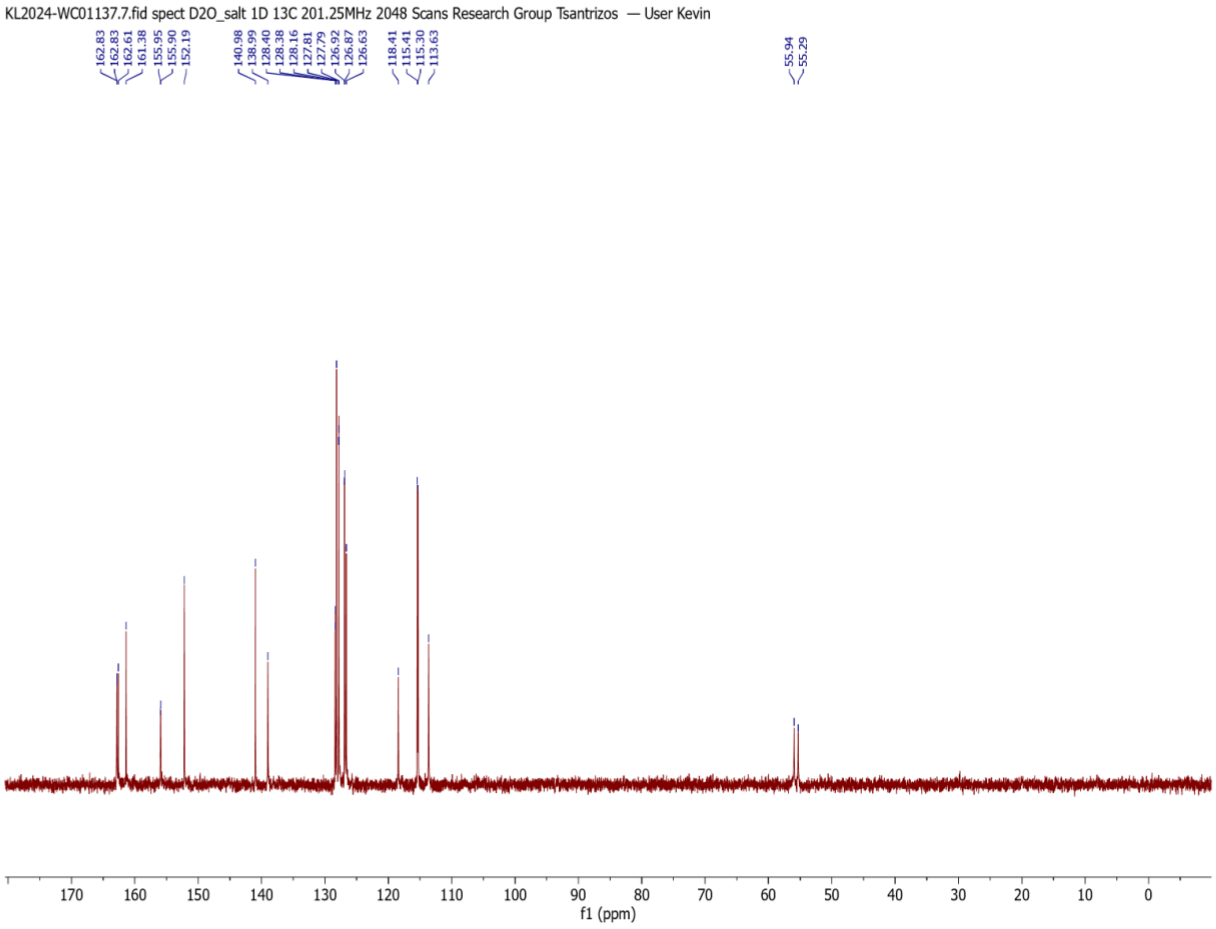

HPLC Chromatogram (**Compound 12**)

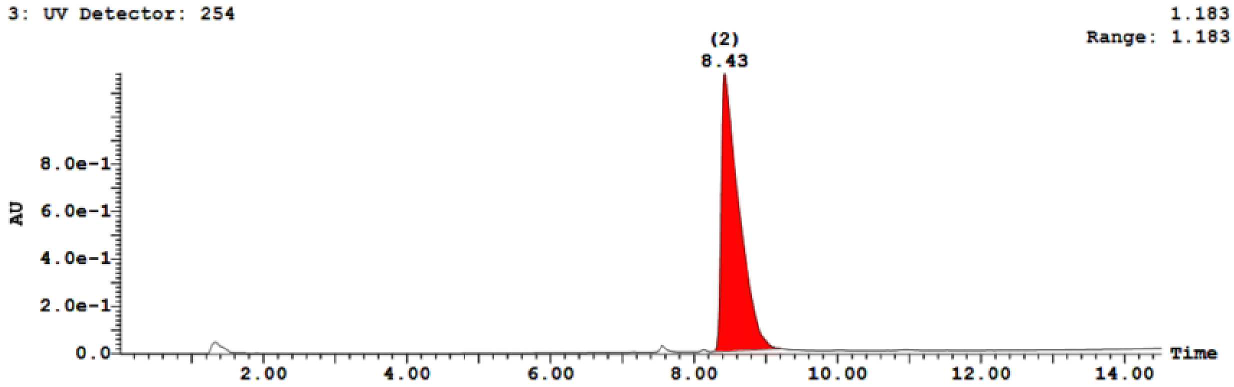

Peak Number	Time	AreaAbs	Area %Total	Height	Mass Found
2	8.43	337173	100.00	1173350	

LRMS (**Compound 12**)

Peak ID	Time	Target
2	8.43	No Target

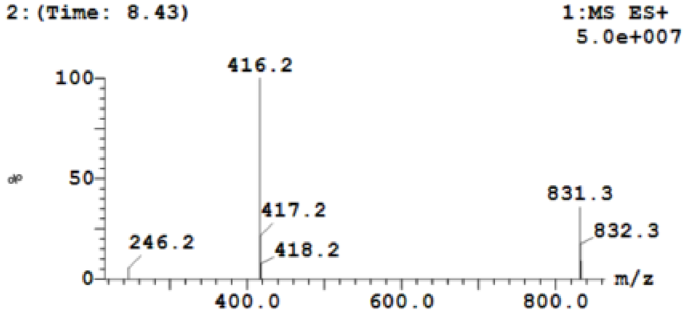

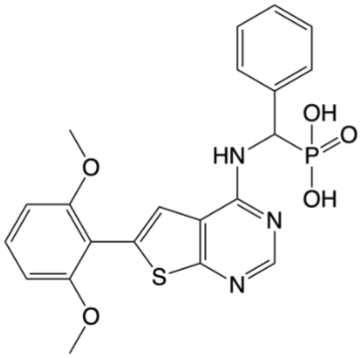

Compound 13

This compound has not been previously reported in the literature.

^{1}H NMR (800 MHz, 0.5% ND_4OD in D_2O) (**Compound 13**)

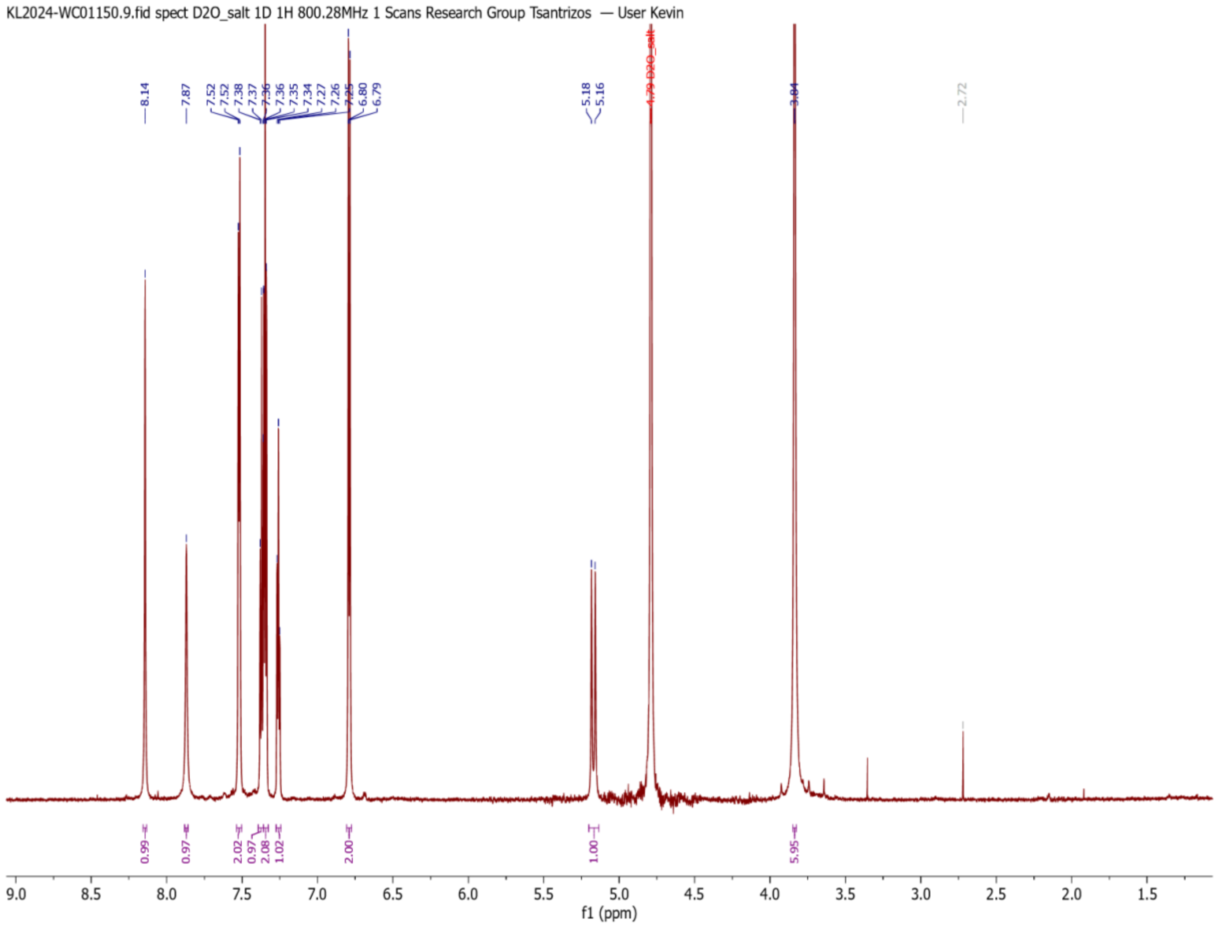

Expansion 1:

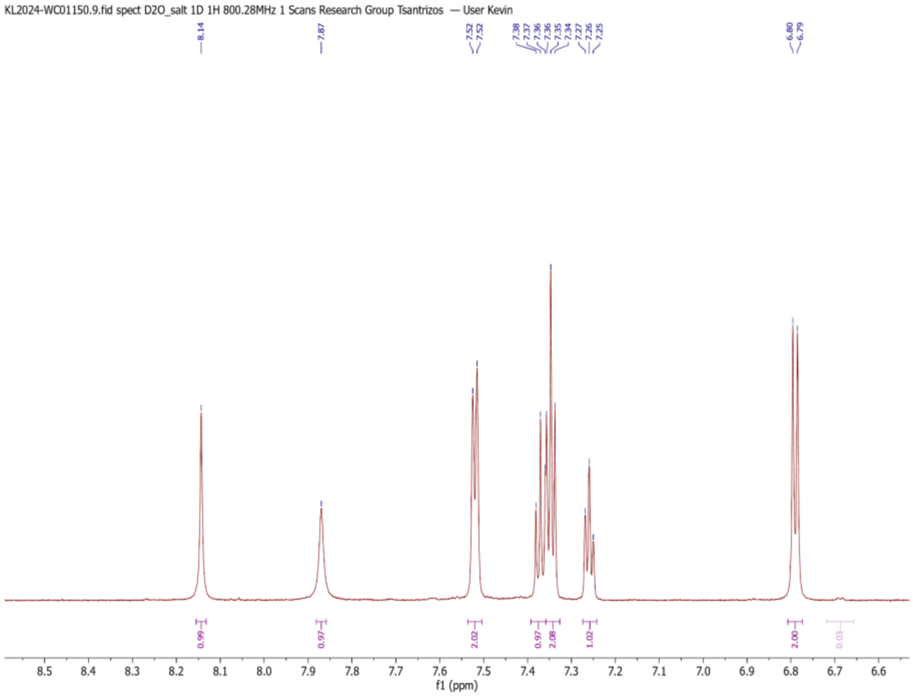

Expansion 2:

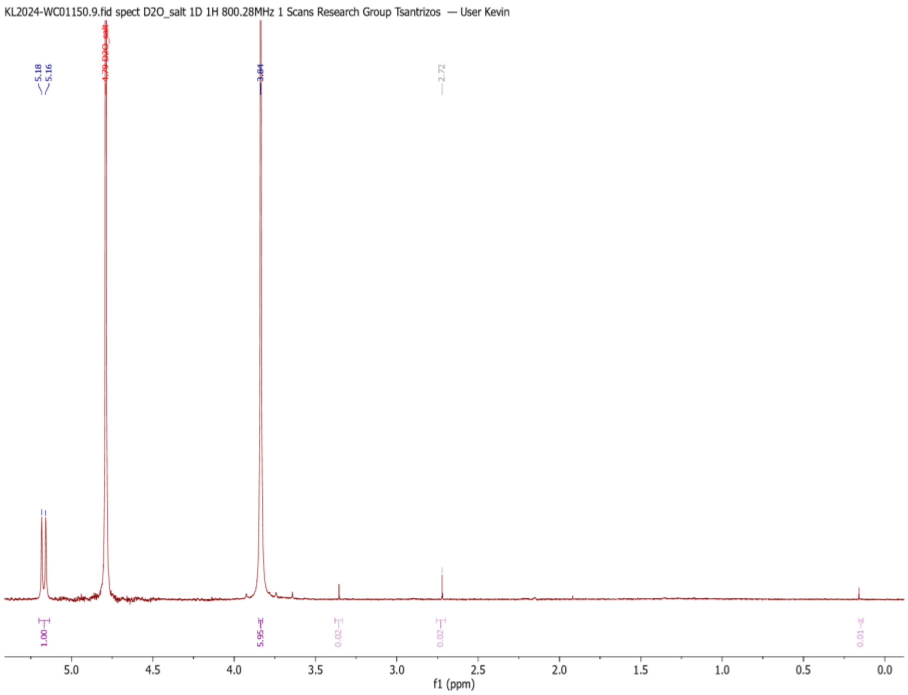

^{31}P NMR (203 MHz, 0.5% ND_4OD in D_2O) (**Compound 13**)

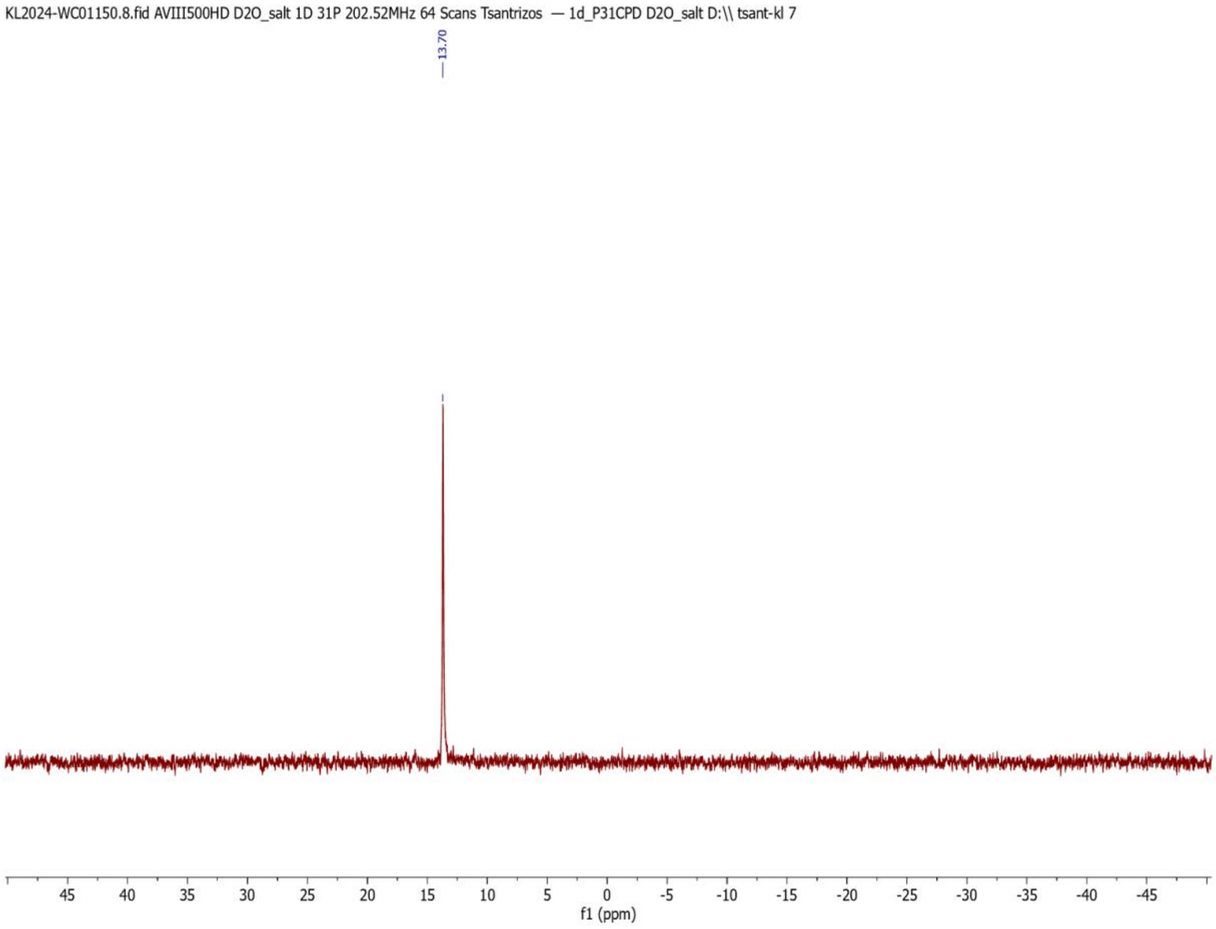

^{13}C NMR (201 MHz, 0.5% ND_4OD in D_2O) (**Compound 13**)

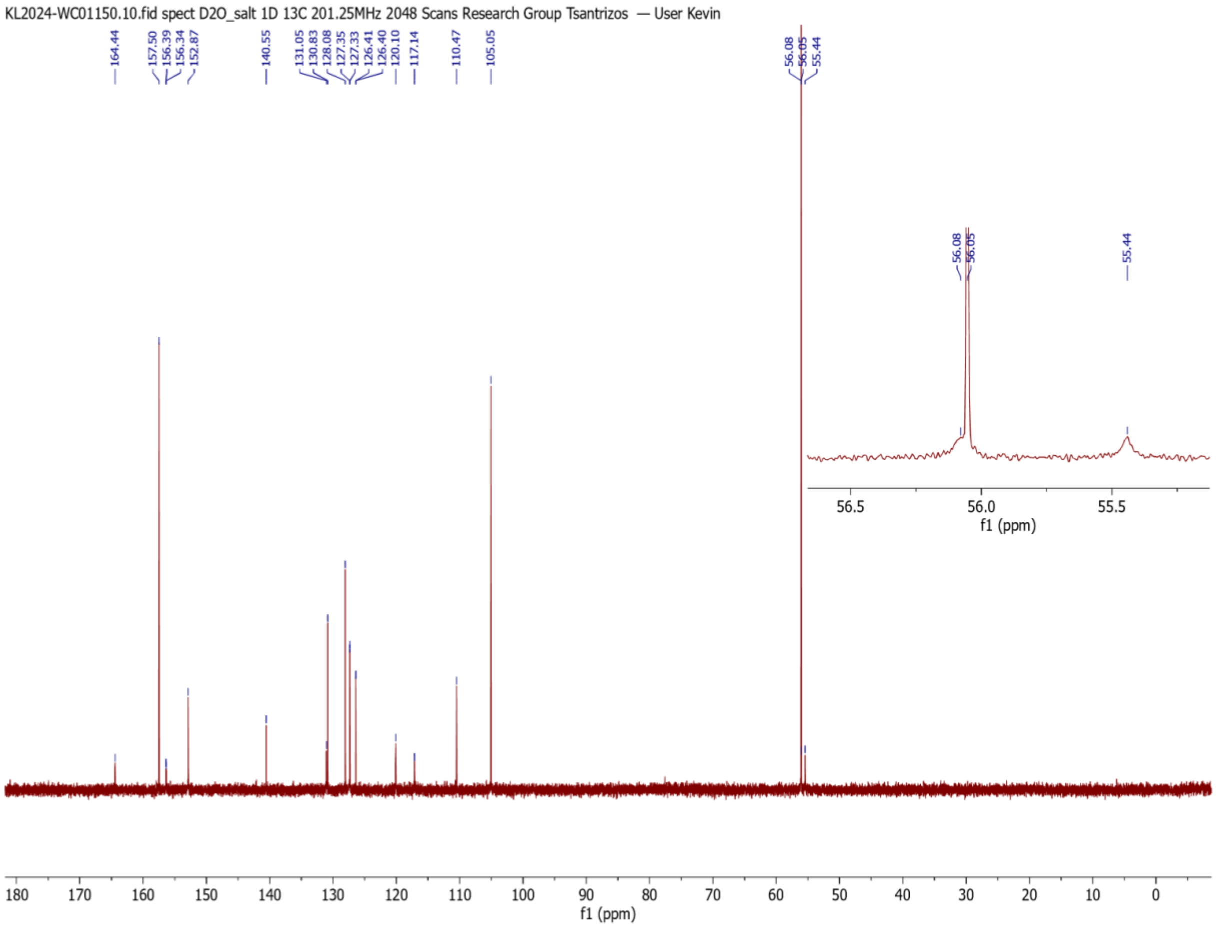

HPLC Chromatogram (**Compound 13**)

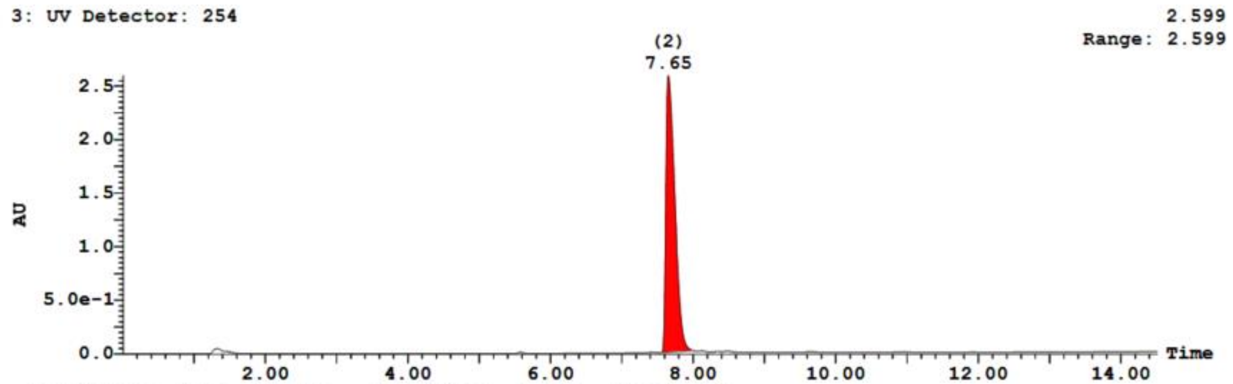

Peak Number	Time	AreaAbs	Area %Total	Height	Mass Found
2	7.65	375864	100.00	2585361	

LRMS (**Compound 13**)

Peak ID	Time	Target
2	7.66	No Target

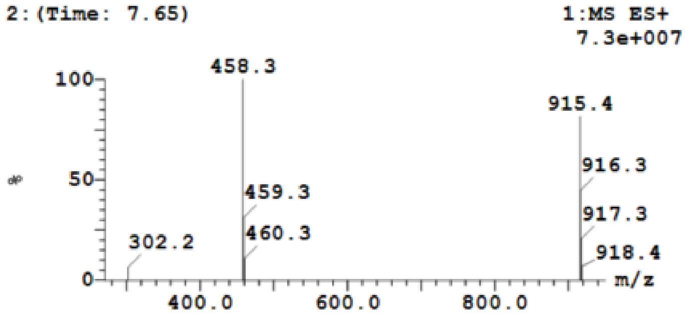

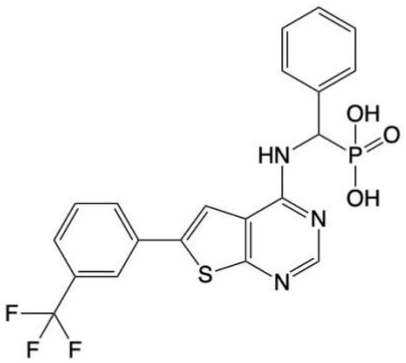

Compound 14

This compound has not been previously reported in the literature.

^{1}H NMR (800 MHz, 0.5% ND_4OD in D_2O) (**Compound 14**)

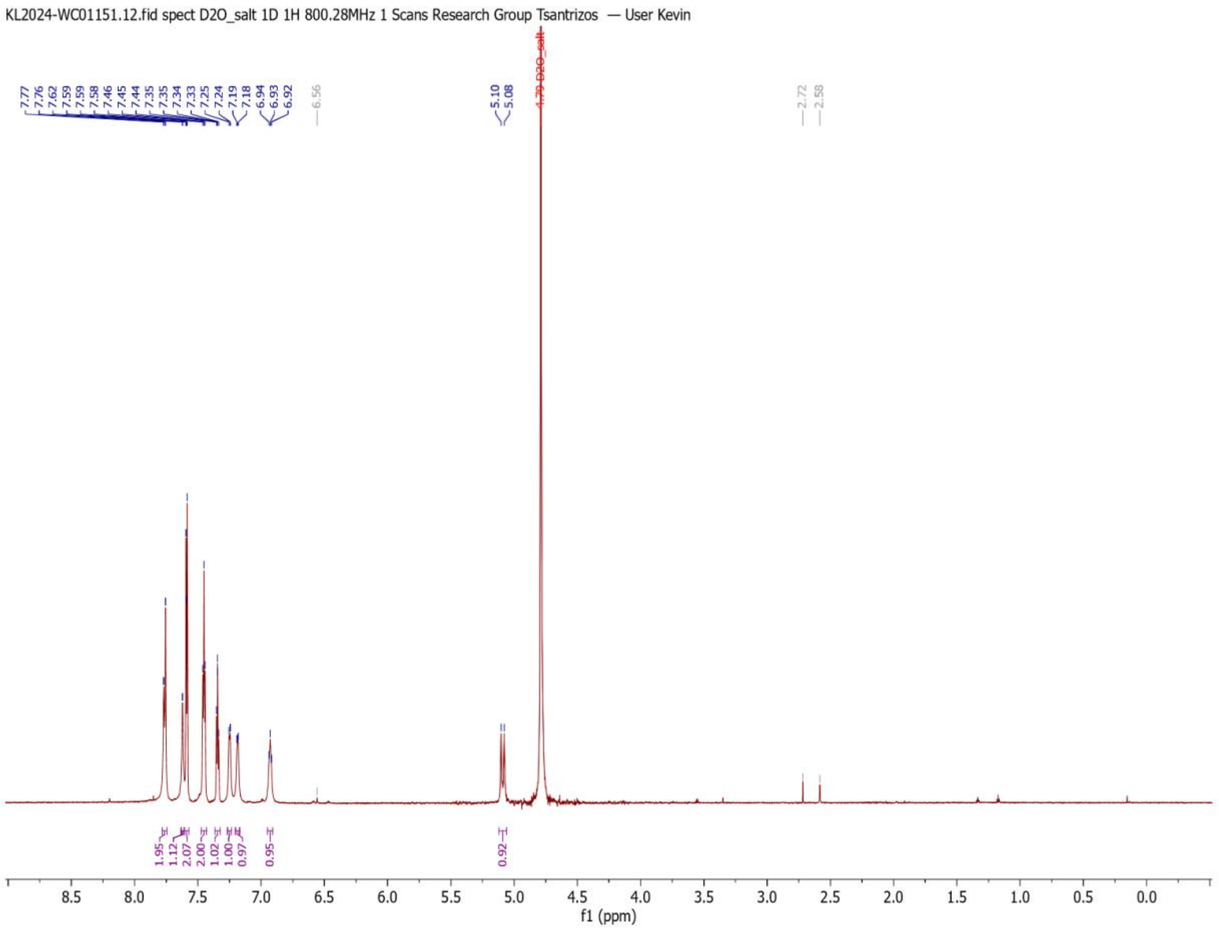

Expansion 1:

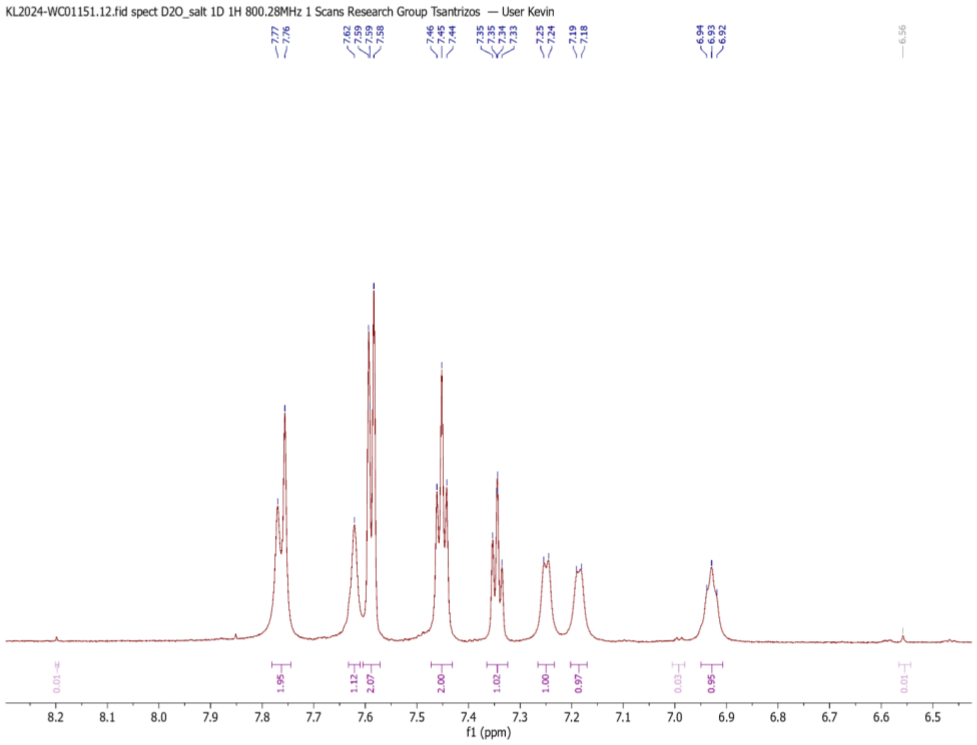

Expansion 2:

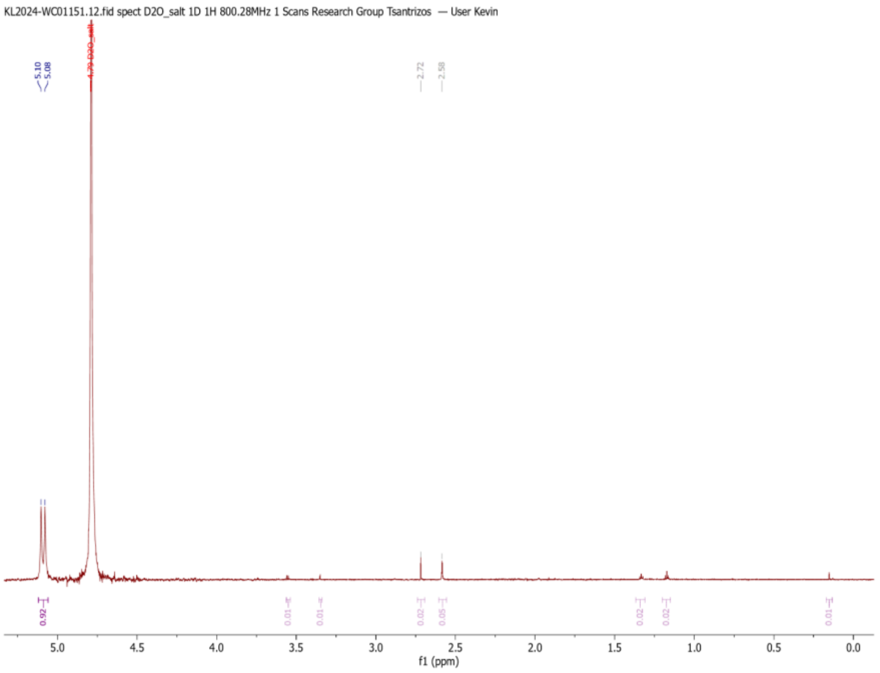

^{31}P NMR (203 MHz, 0.5% ND_4OD in D_2O) (**Compound 14**)

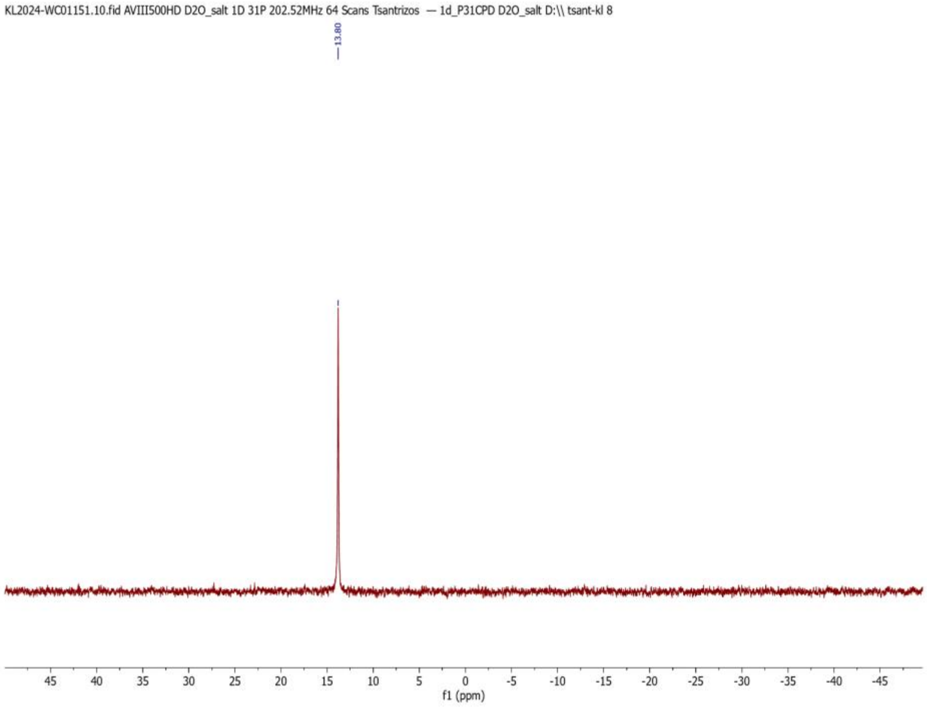

^{19}F NMR (470 MHz, 0.5% ND_4OD in D_2O) (**Compound 14**)

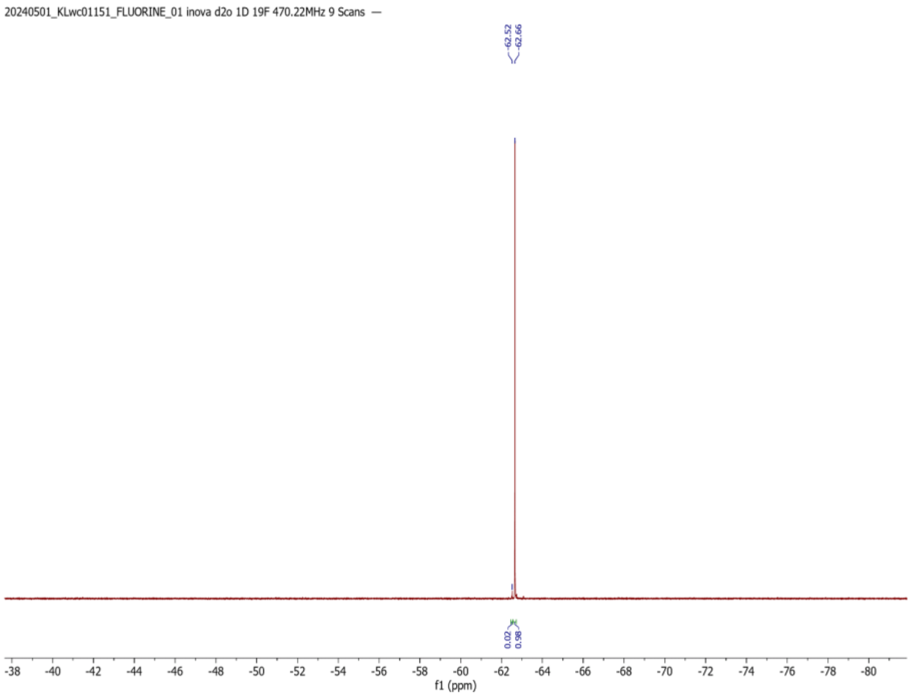

^{13}C NMR (201 MHz, 0.5% ND_4OD in D_2O) (**Compound 14**)

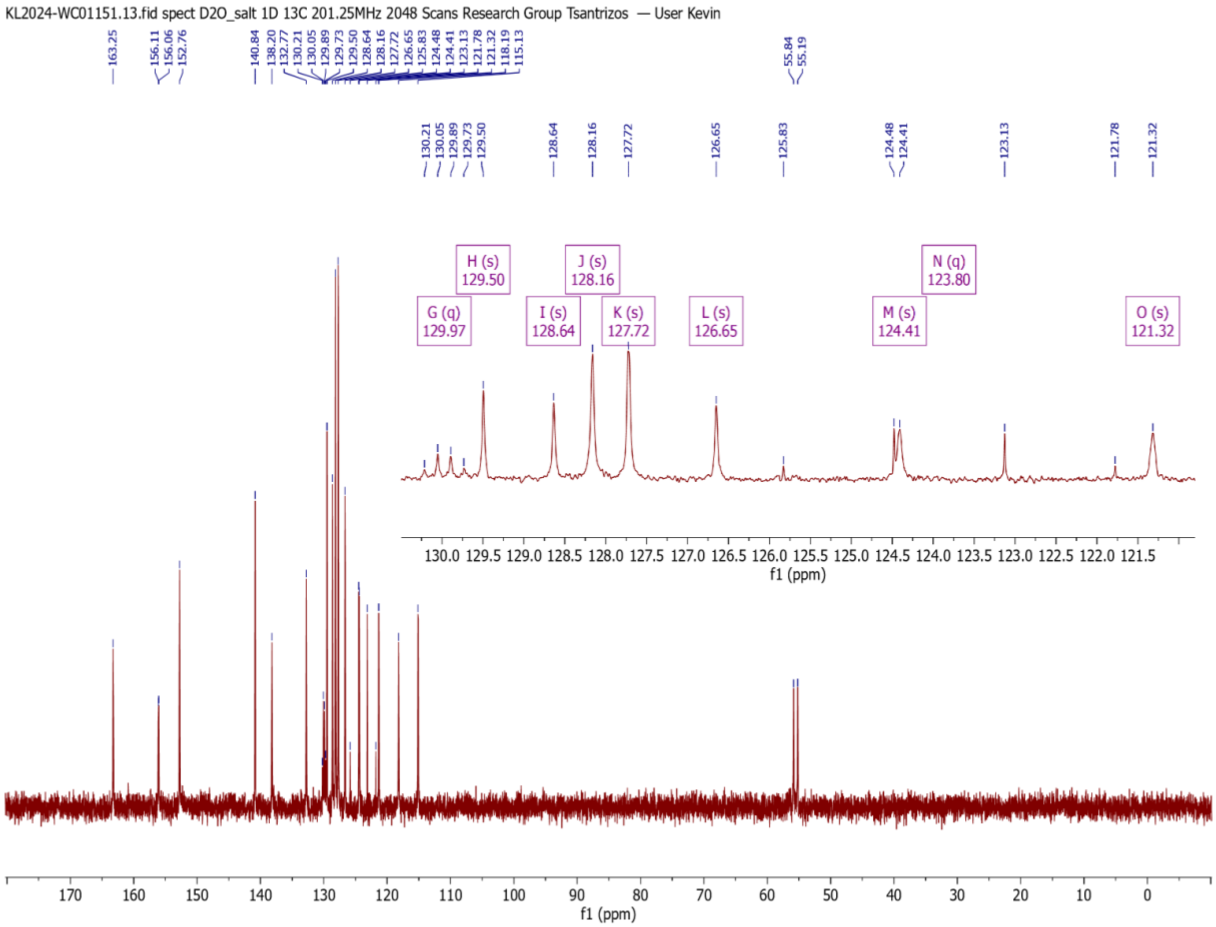

HPLC Chromatogram (**Compound 14**)

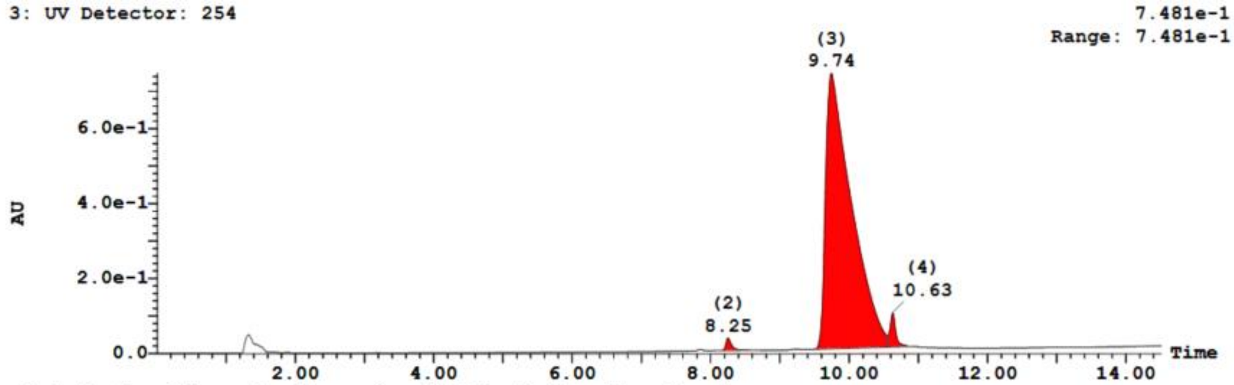

Peak Number	Time	AreaAbs	Area %Total	Height	Mass Found
2	8.25	3384	1.01	33288	
3	9.74	321462	96.25	736293	
4	10.63	9149	2.74	90731	

LRMS (**Compound 14**)

Peak ID	Time	Target
3	9.74	No Target

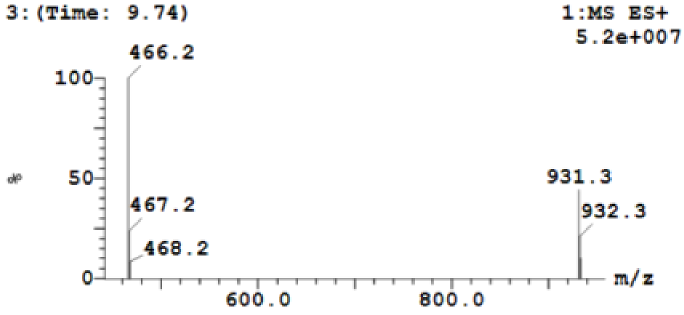

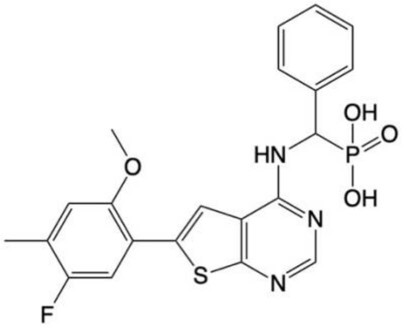

Compound 15

The synthesis of this compound has been previously reported (compound **23** in reference 30). However, the spectra had not been given in the previous publication and are thus included here.

^{1}H NMR (800 MHz, 0.5% ND_4OD in D_2O) (**Compound 15**)

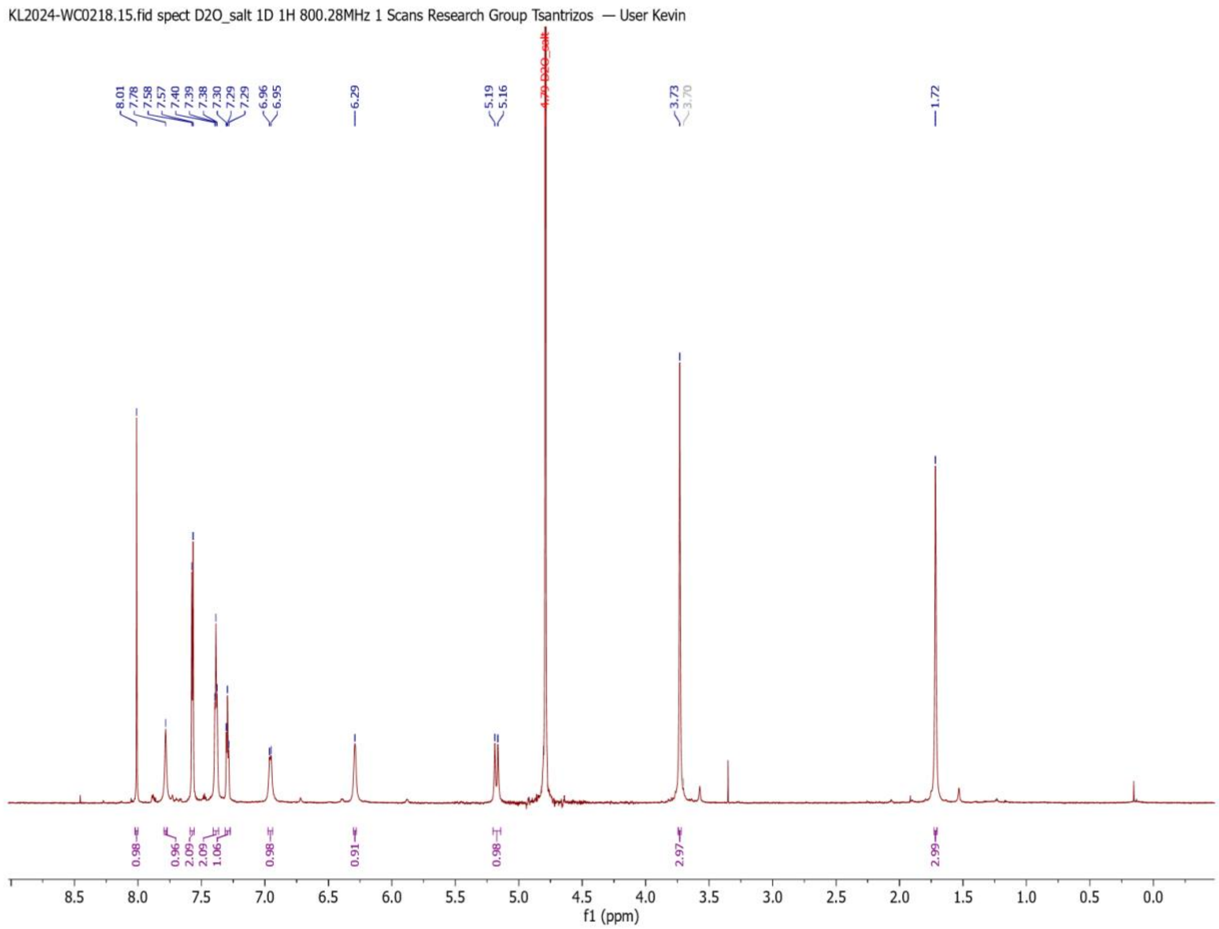

Expansion 1:

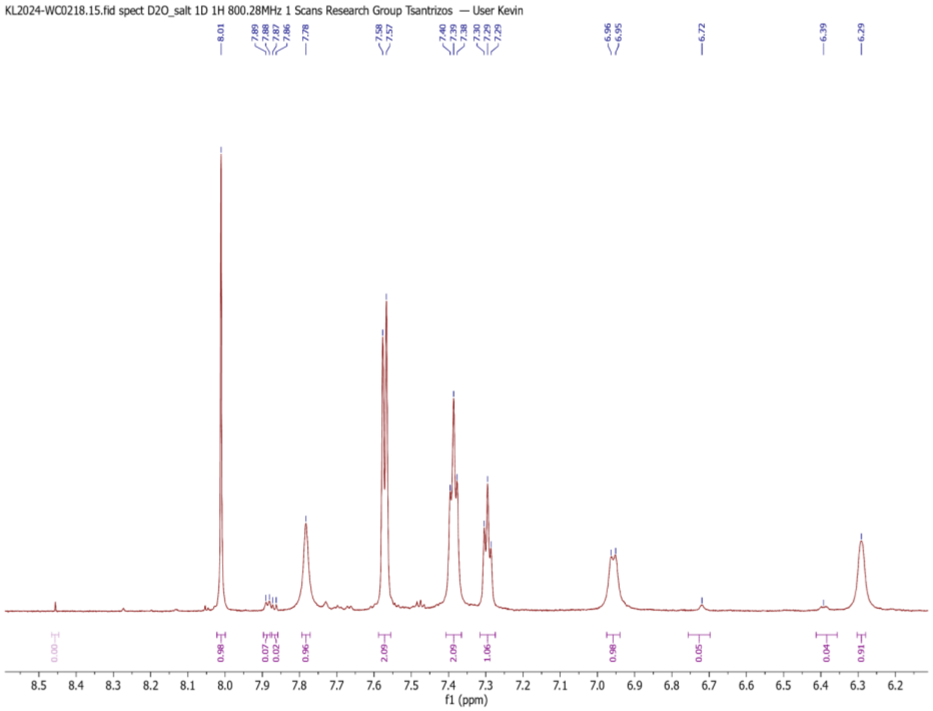

Expansion 2:

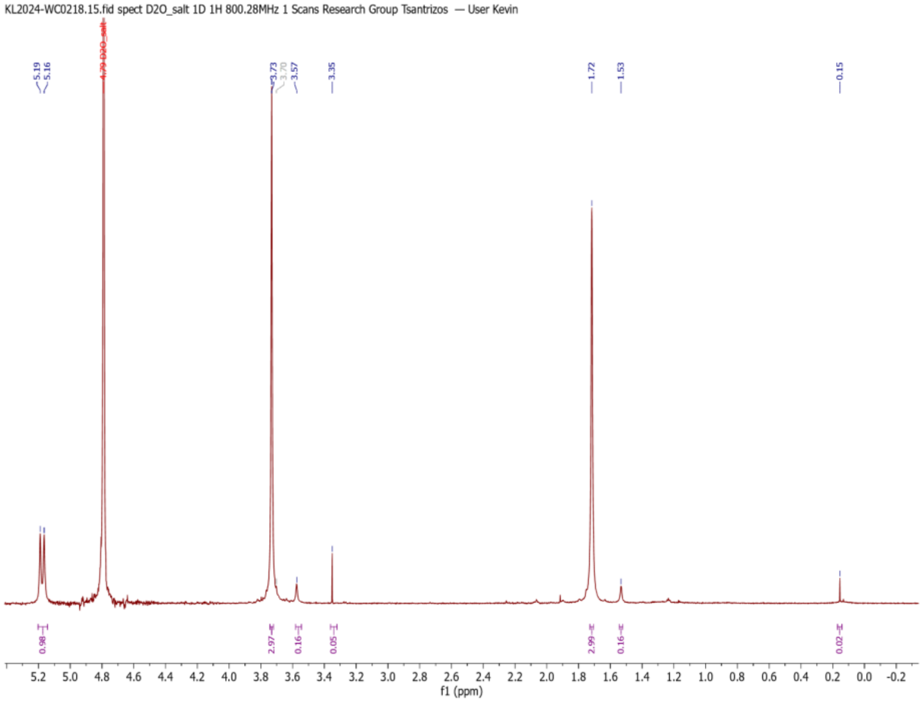

^{31}P NMR (203 MHz, 0.5% ND_4OD in D_2O) (**Compound 15**)

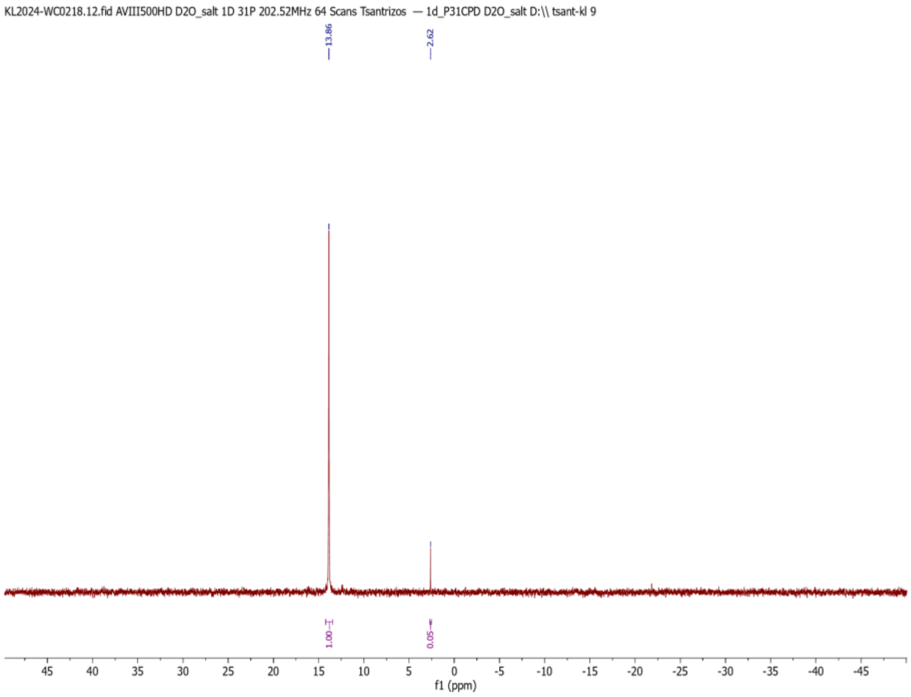

^{19}F NMR (377 MHz, 0.5% ND_4OD in D_2O) (**Compound 15**)

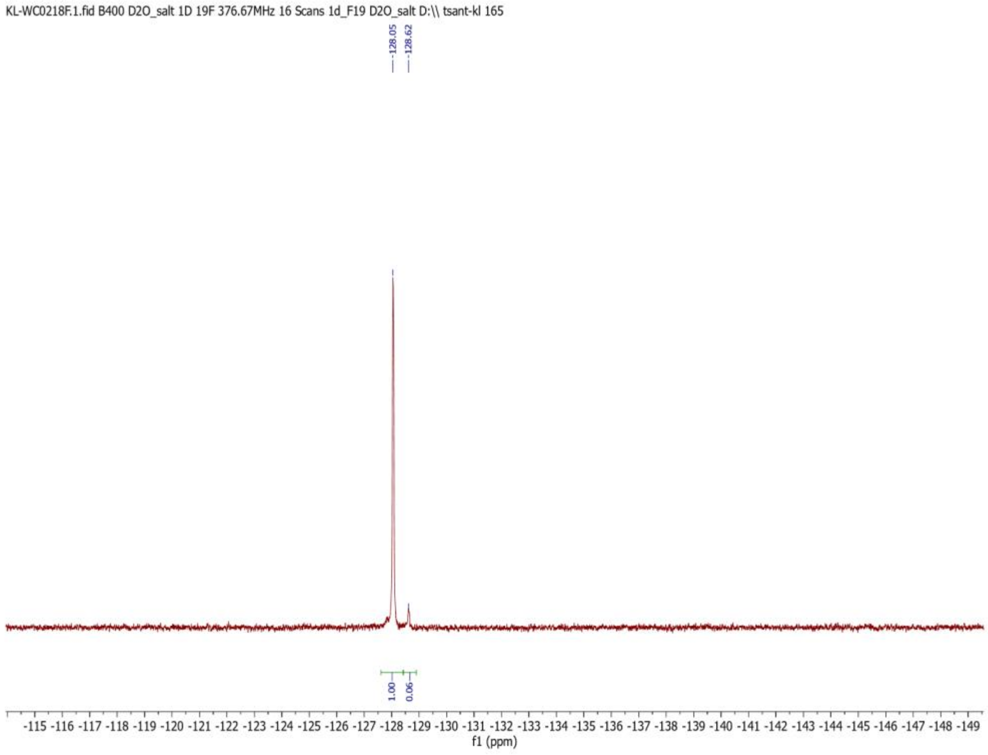

^{13}C NMR (201 MHz, 0.5% ND_4OD in D_2O) (**Compound 15**)

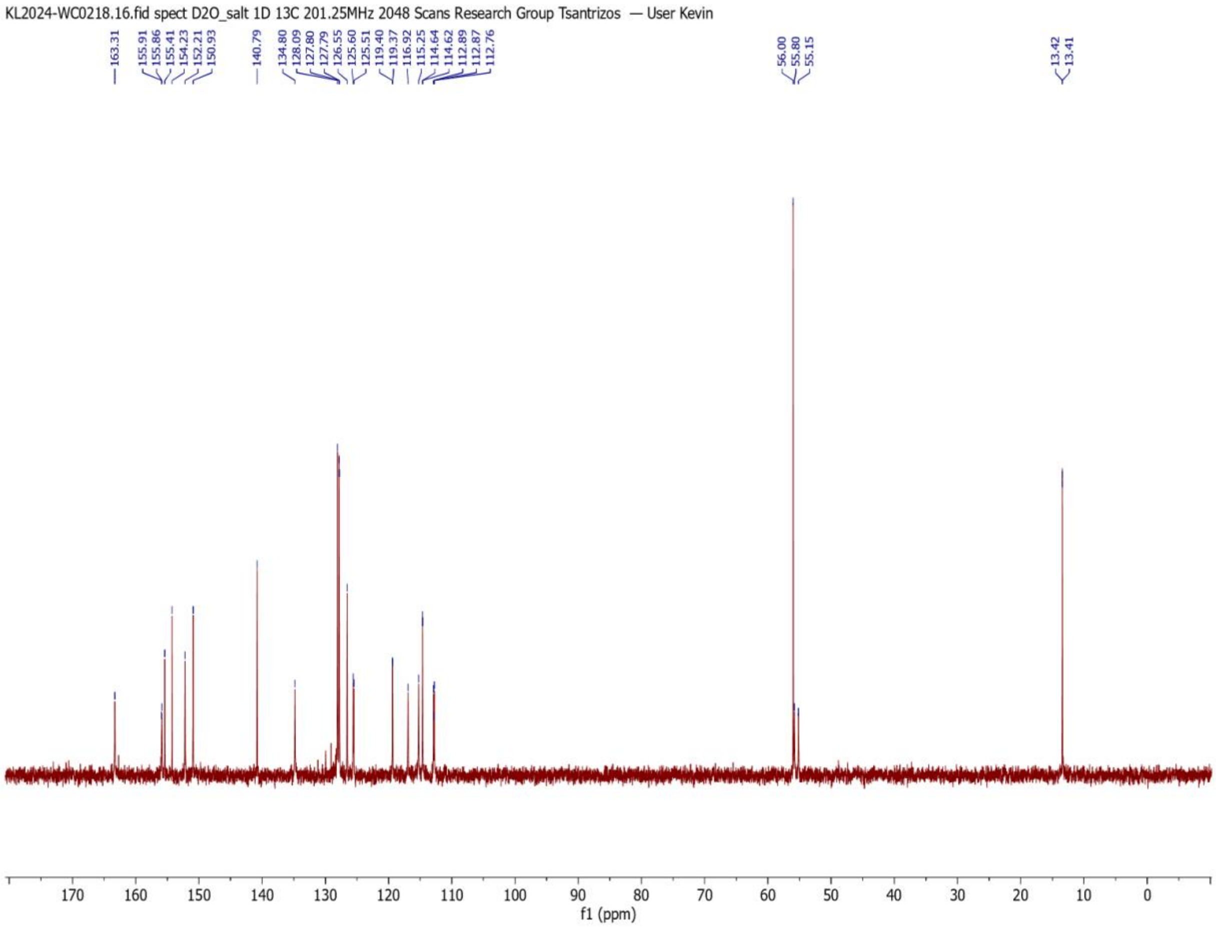

HPLC Chromatogram (**Compound 15**)

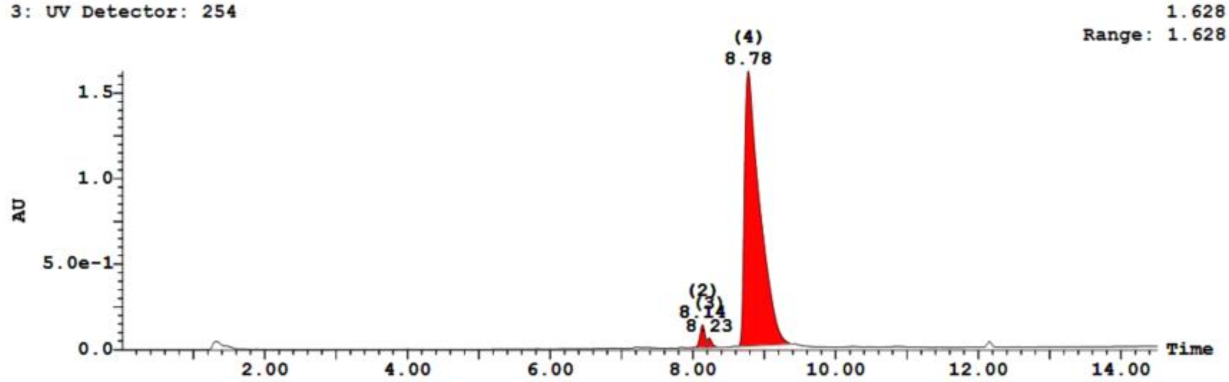

Peak Number	Time	AreaAbs	Area %Total	Height	Mass Found
2	8.14	10656	2.57	134522	
3	8.23	4350	1.05	55293	
4	8.78	399085	96.38	1606430	

LRMS (**Compound 15**)

Peak ID	Time	Target
4	8.78	No Target

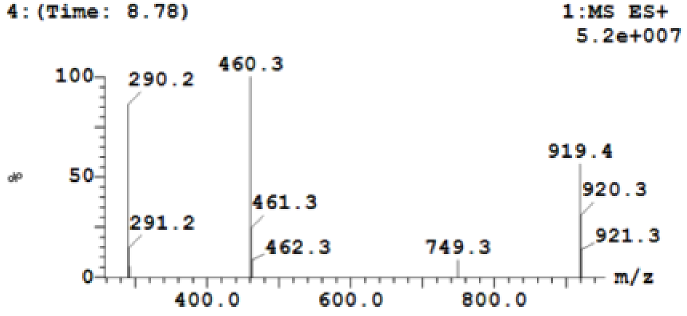

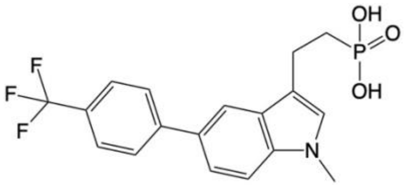

Compound 16

This compound has not been previously reported in the literature.

HPLC Chromatogram (**Compound 16**)

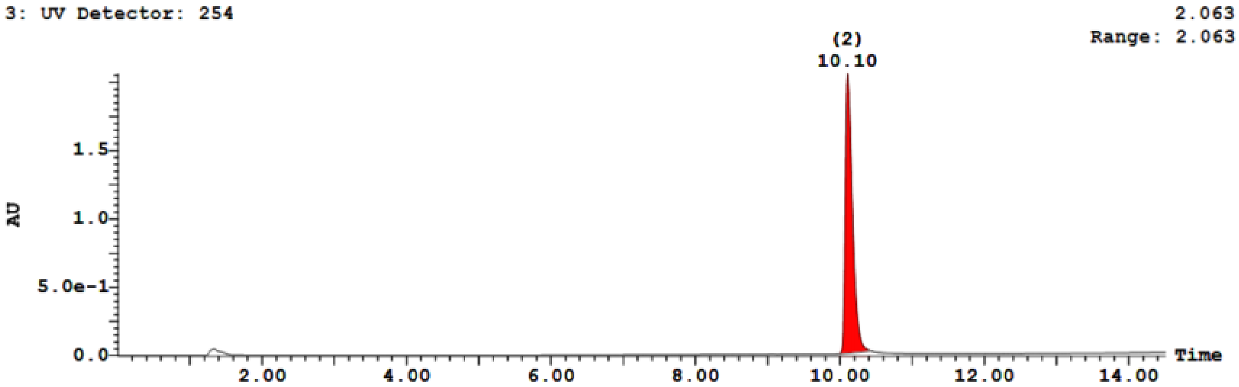

Peak Number	Time	AreaAbs	Area %Total	Height	Mass Found
2	10.10	242311	100.00	2046414	

LRMS (**Compound 16**)

Peak ID	Time	Target
2	10.10	No Target

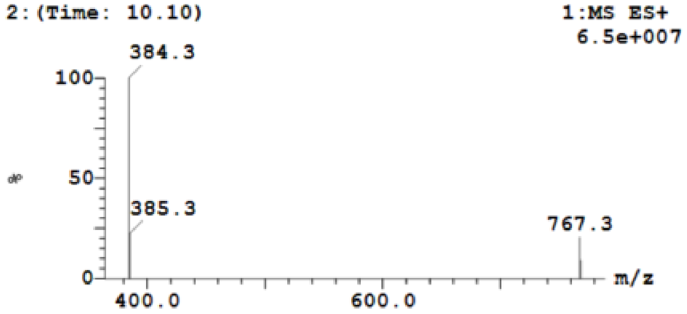

^{1}H NMR (500 MHz) & ^{13}C NMR (125 MHz), 0.5% ND_4OD in D_2O (**Compound 16**)

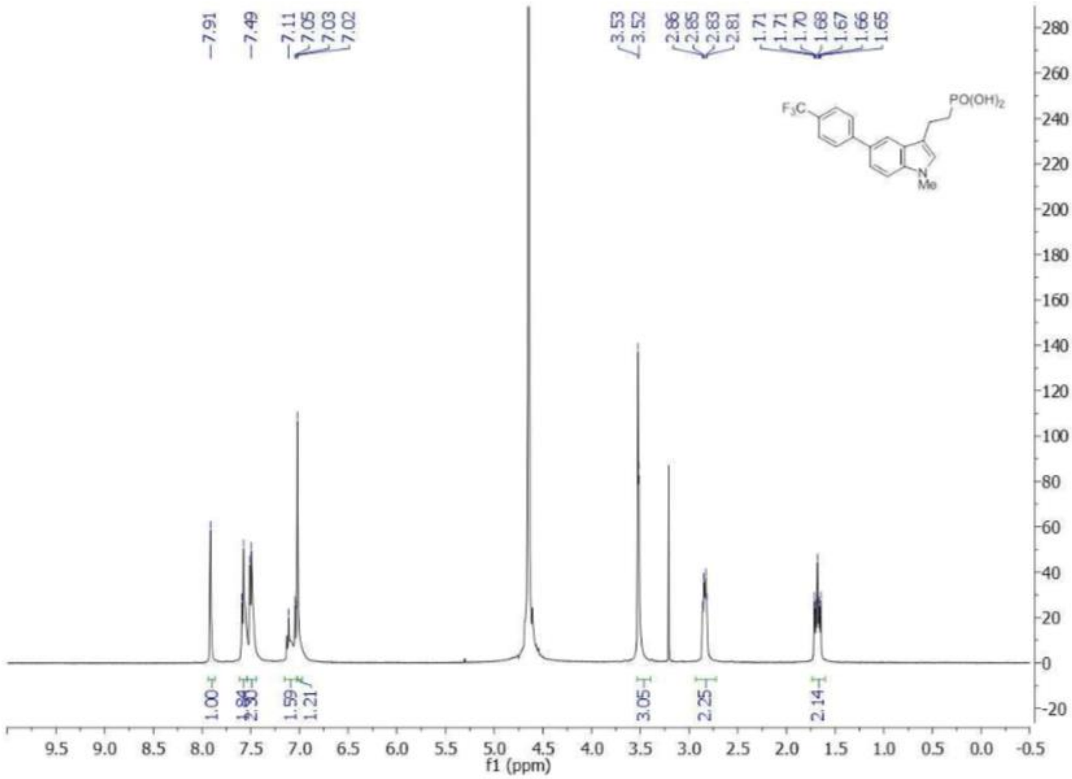

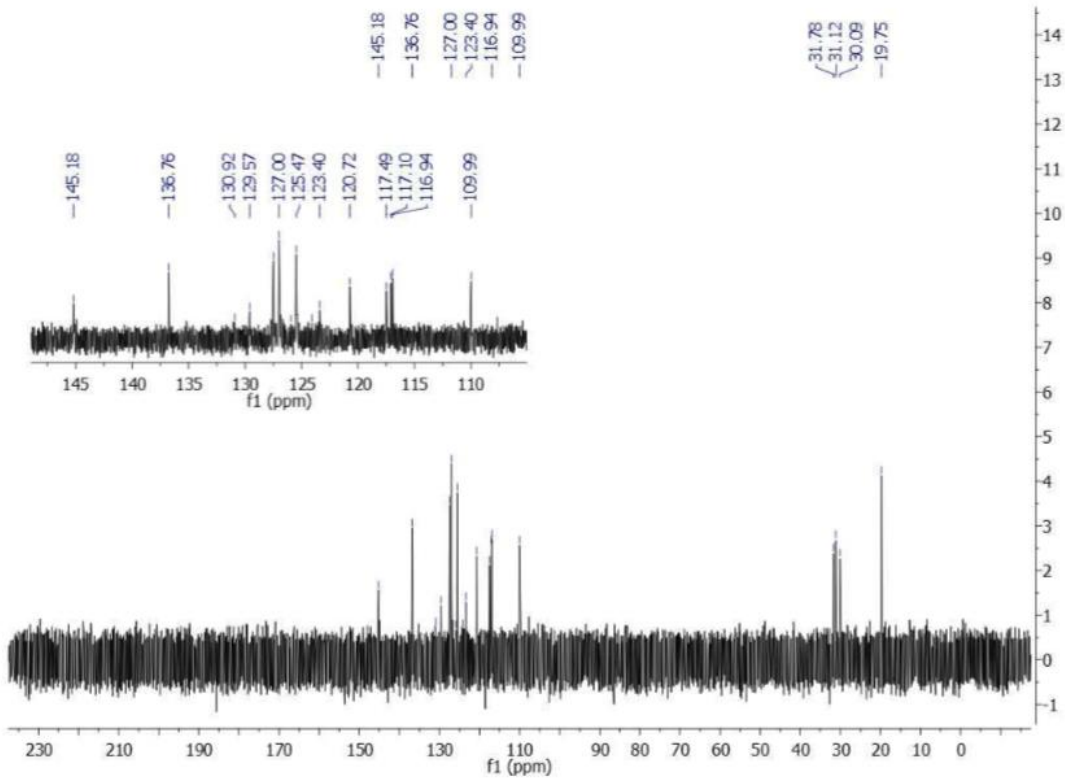

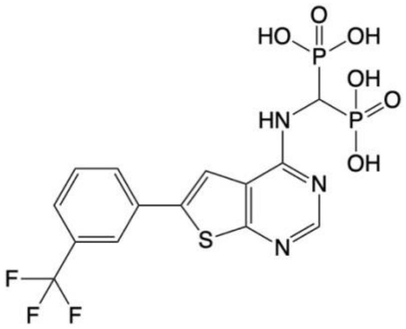

Compound 17

This compound has been previously reported (compound **6i** in reference 36).

HPLC Chromatogram (**Compound 17**)

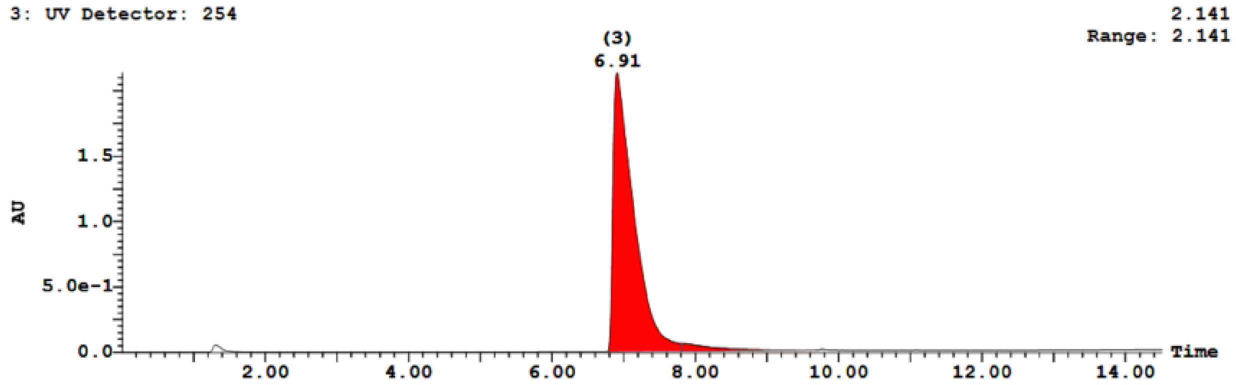

Peak Number	Time	AreaAbs	Area %Total	Height	Mass Found
3	6.91	715240	95.74	2136309	
4	7.85	31789	4.26	57357	

LRMS (**Compound 16**)

Peak ID	Time	Target
3	6.91	No Target

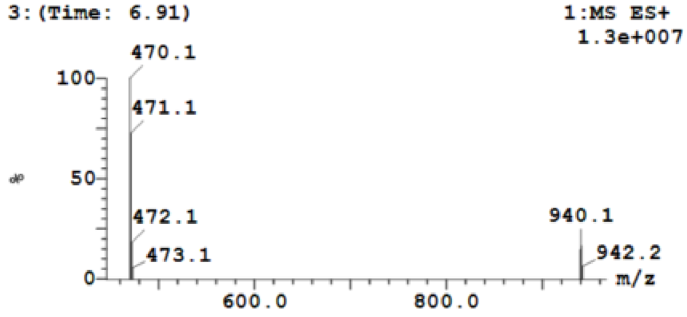

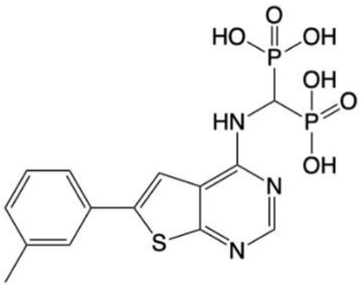

Compound 18

This compound has been previously reported (compound **6g** in reference 36).

HPLC Chromatogram (**Compound 18**)

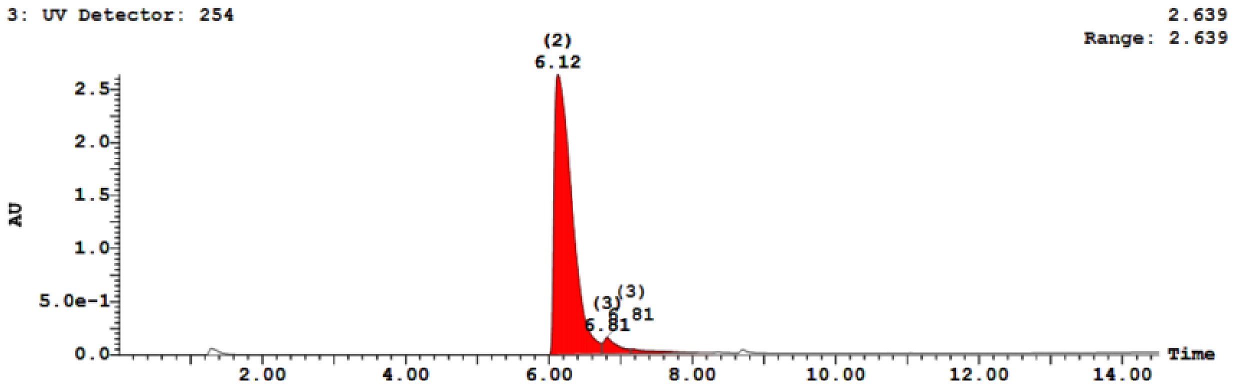

Peak Number	Time	AreaAbs	Area %Total	Height	Mass Found
2	6.12	757298	93.53	2635060	
3	6.81	35156	4.34	153402	
4	7.18	17203	2.12	39170	

LRMS (**Compound 18**)

Peak ID	Time	Target
2	6.13	No Target

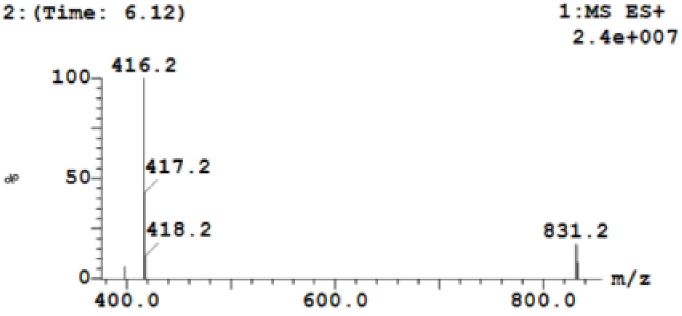

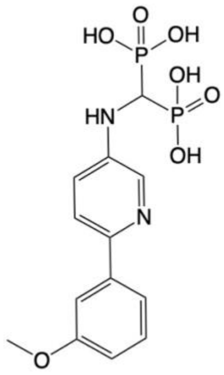

Compound 19

This compound has been previously reported (compound **11m** in reference 33). However, the spectra had not been given in the previous publication and are thus included here.

Due to solubility issues, the HPLC chromatogram was not taken.

^{1}H NMR (800 MHz, 0.5% ND_4OD in D_2O) (**Compound 19**)

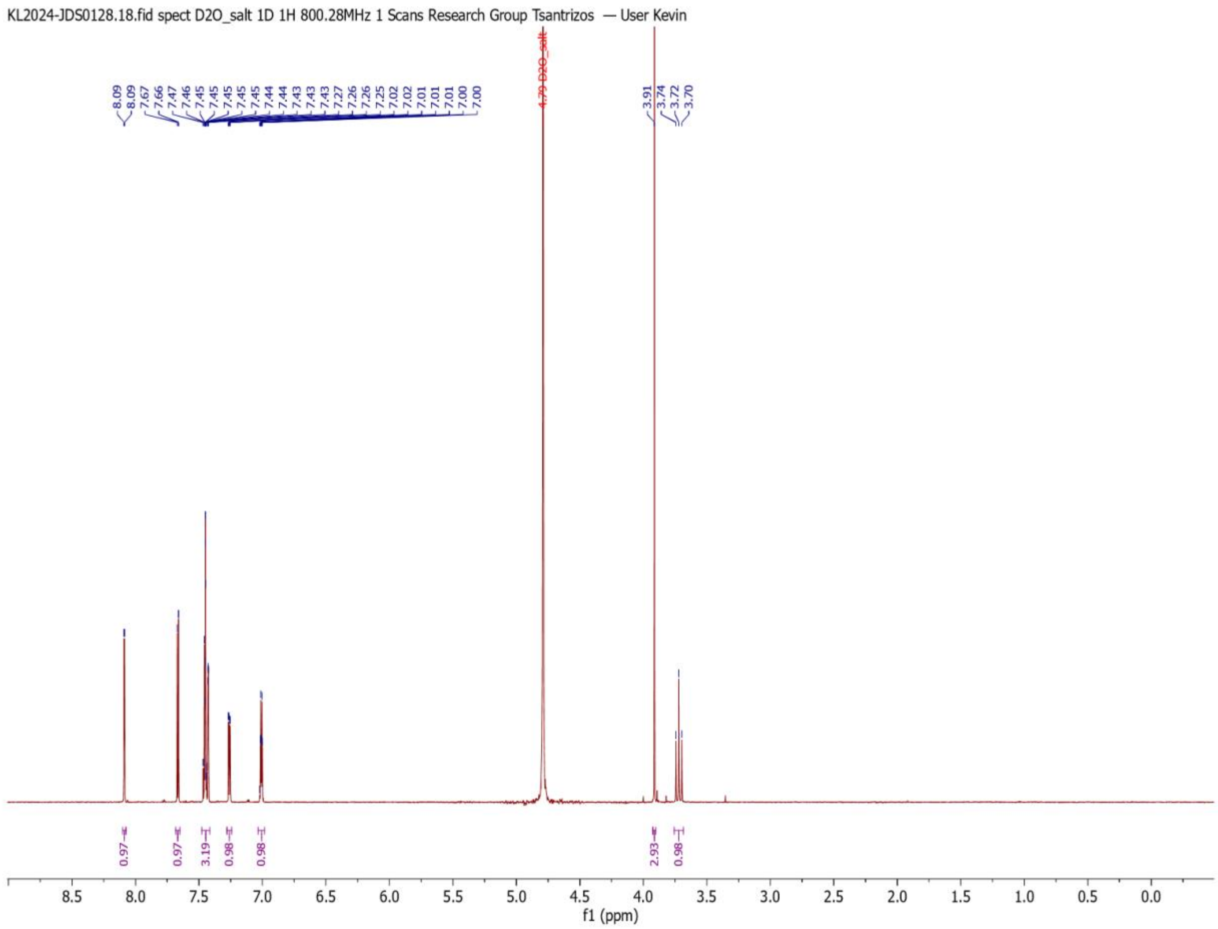

Expansion 1:

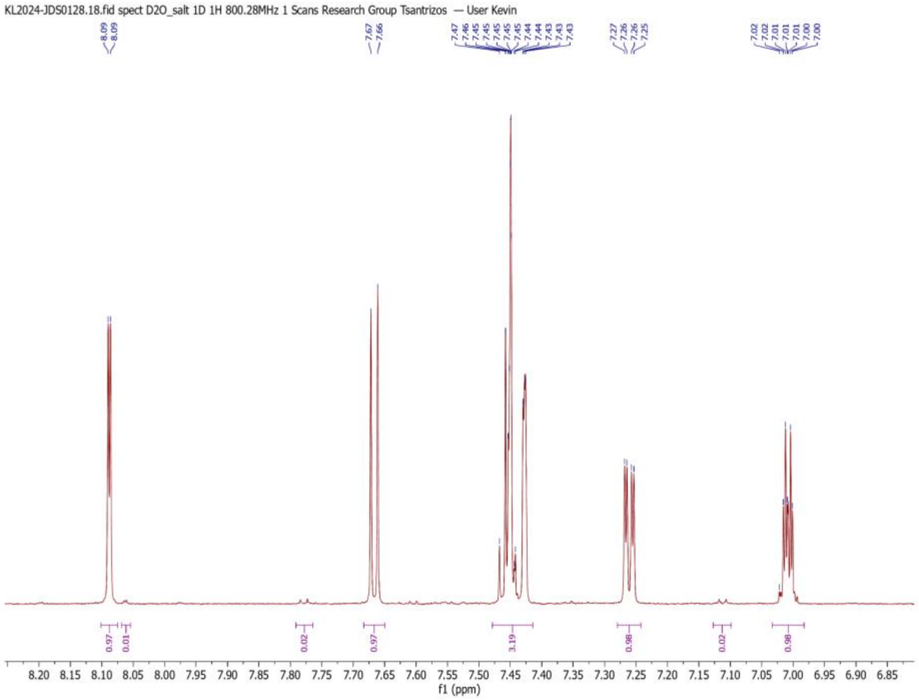

Expansion 2:

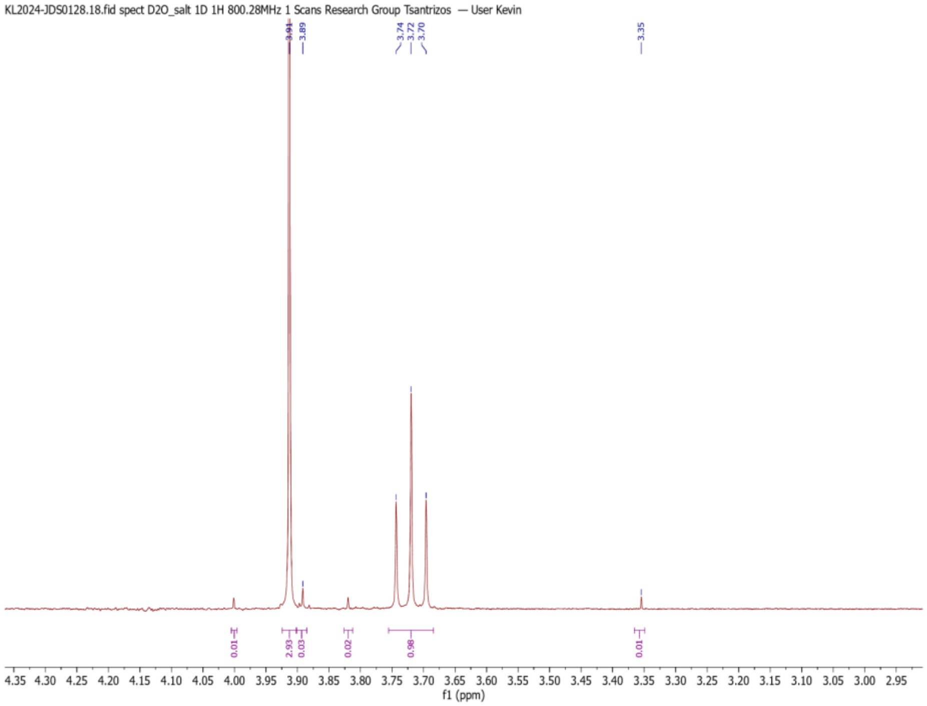

^{31}P NMR (203 MHz, 0.5% ND_4OD in D_2O) (**Compound 19**)

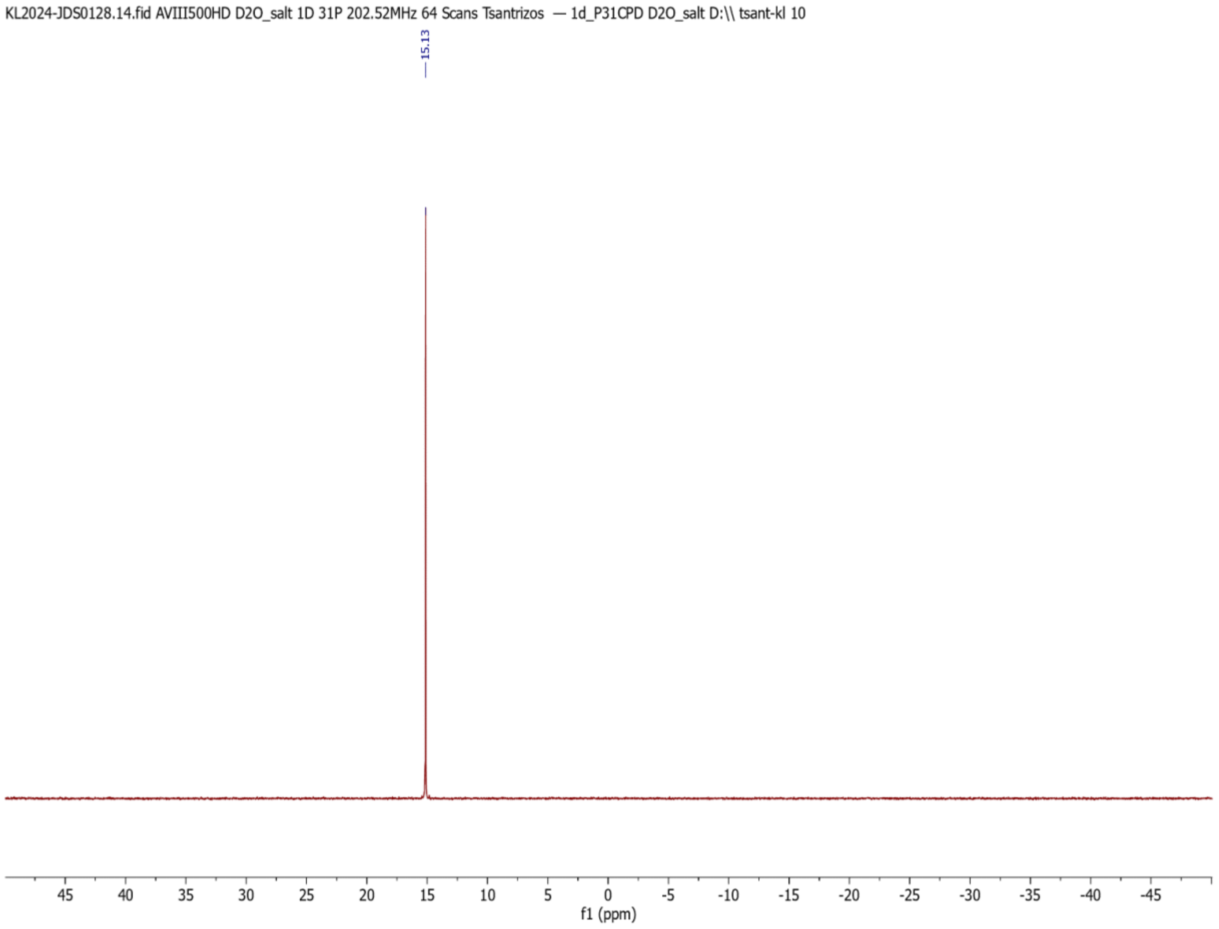

^{13}C NMR (201 MHz, 0.5% ND_4OD in D_2O) (**Compound 19**)

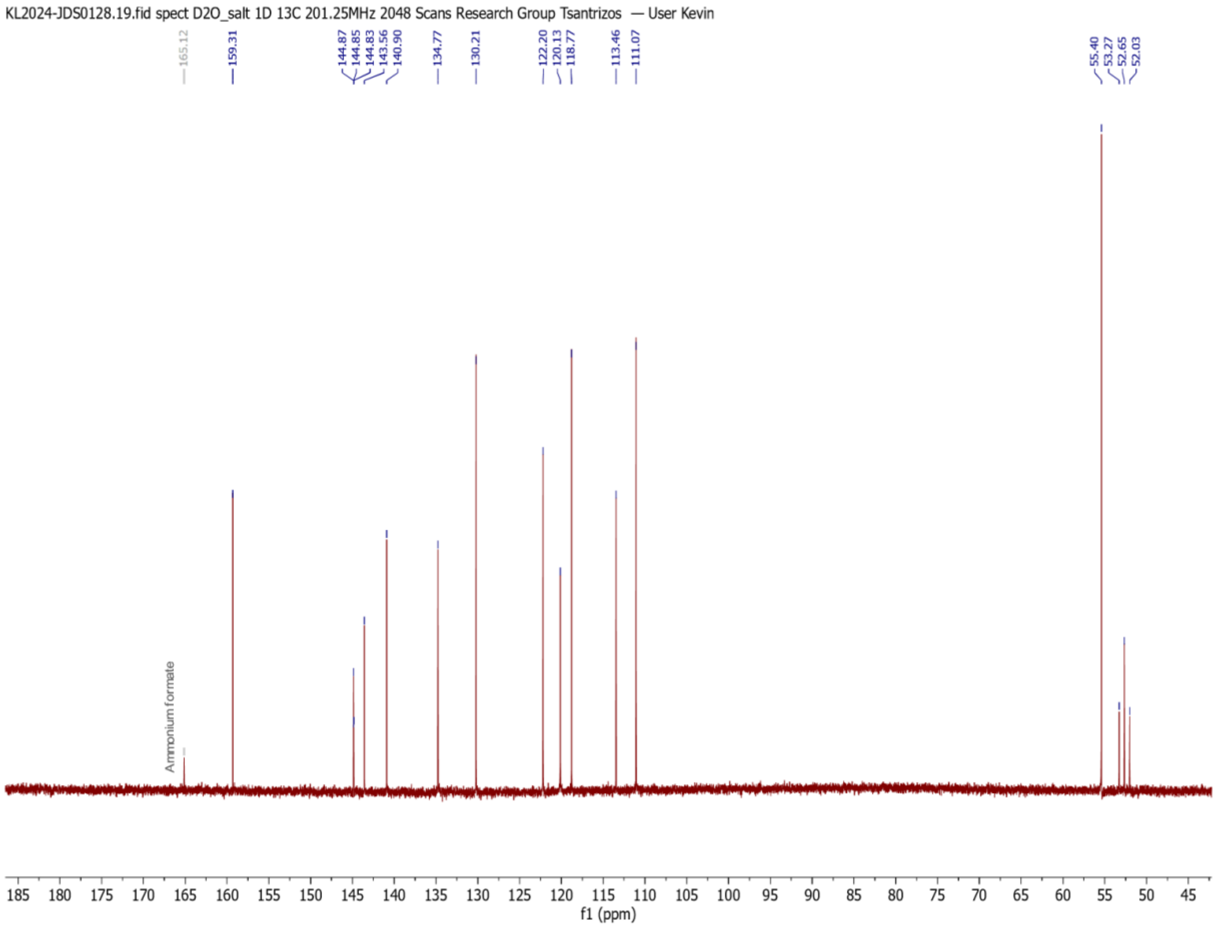

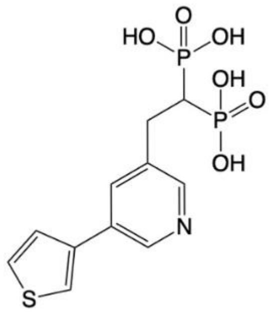

Compound 20

This compound has been previously reported (compound **9a** in reference 35).

HPLC Chromatogram (**Compound 20**)

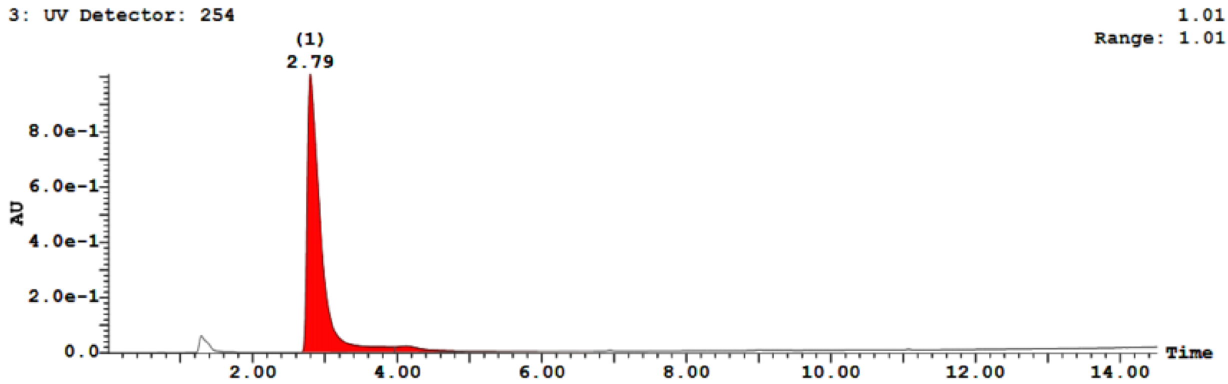

Peak Number	Time	AreaAbs	Area %Total	Height	Mass Found
1	2.79	218141	94.61	1009181	
3	4.13	12421	5.39	21536	

LRMS (**Compound 20**)

Peak ID	Time	Target
1	2.79	No Target

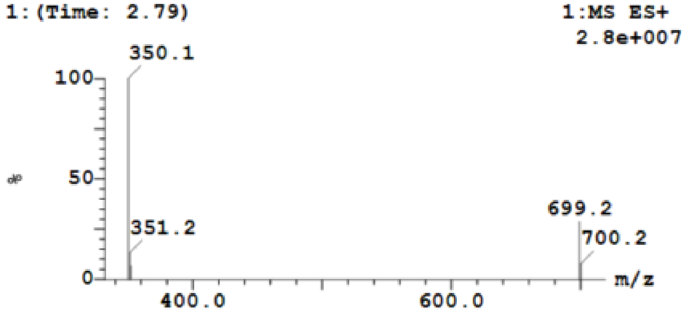

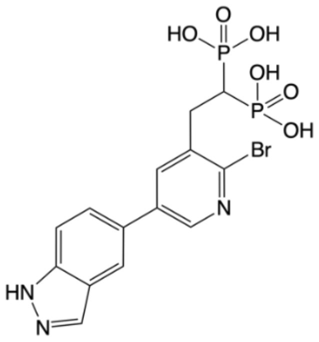

Compound 21

This compound has been previously reported (compound **33** in reference 39). However, the spectra had not been given in the previous publication and are thus included here. This compound forms some aggregates in the NMR solution at fairly high concentrations, leading to a doubling of some peaks in 1:4 ratio.

^{1}H NMR (800 MHz, 0.5% ND_4OD in D_2O) (**Compound 21**)

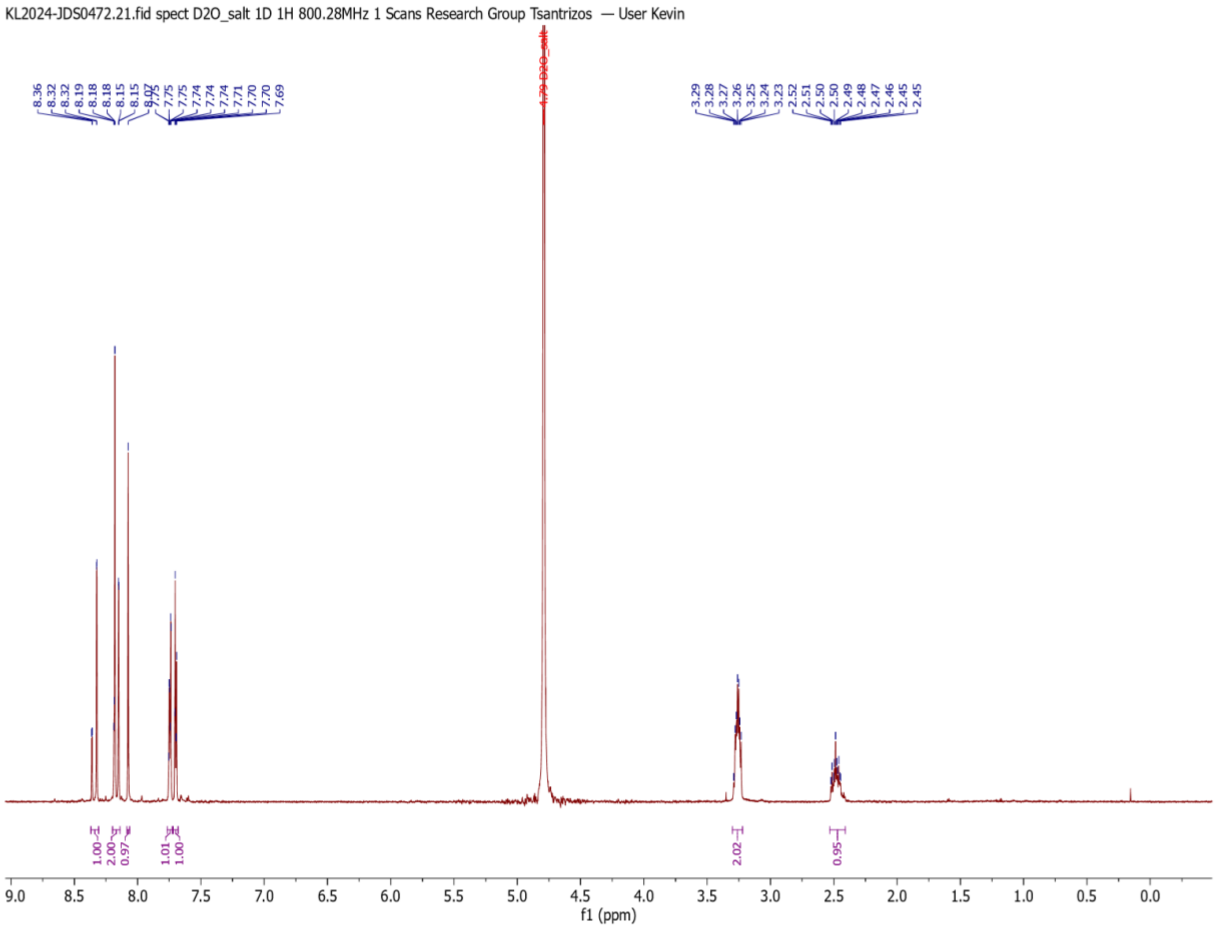

Expansion 1:

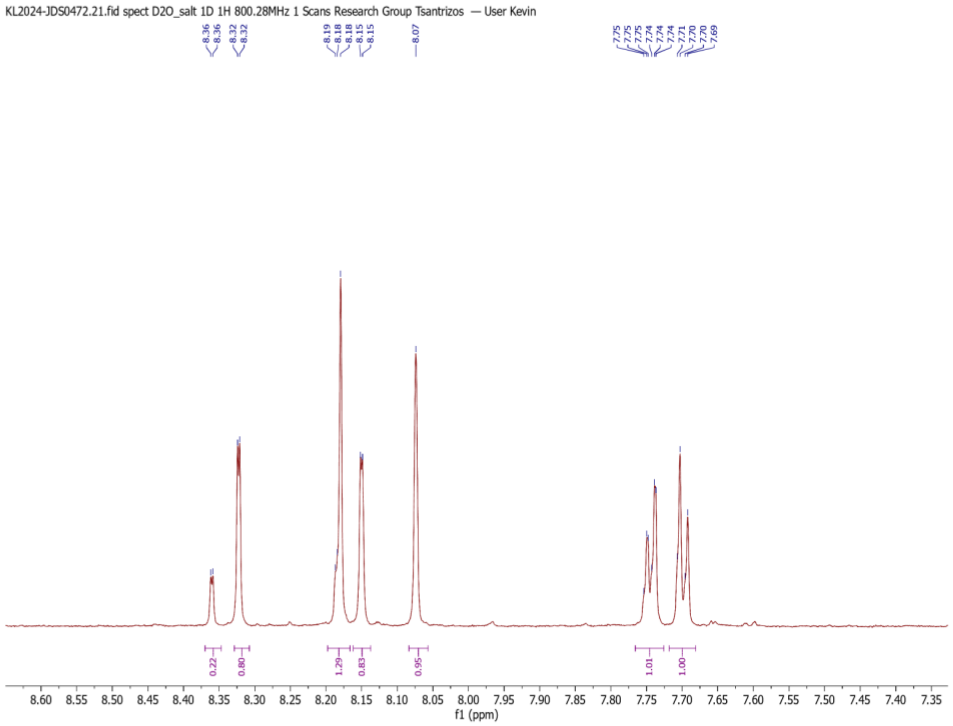

Expansion 2:

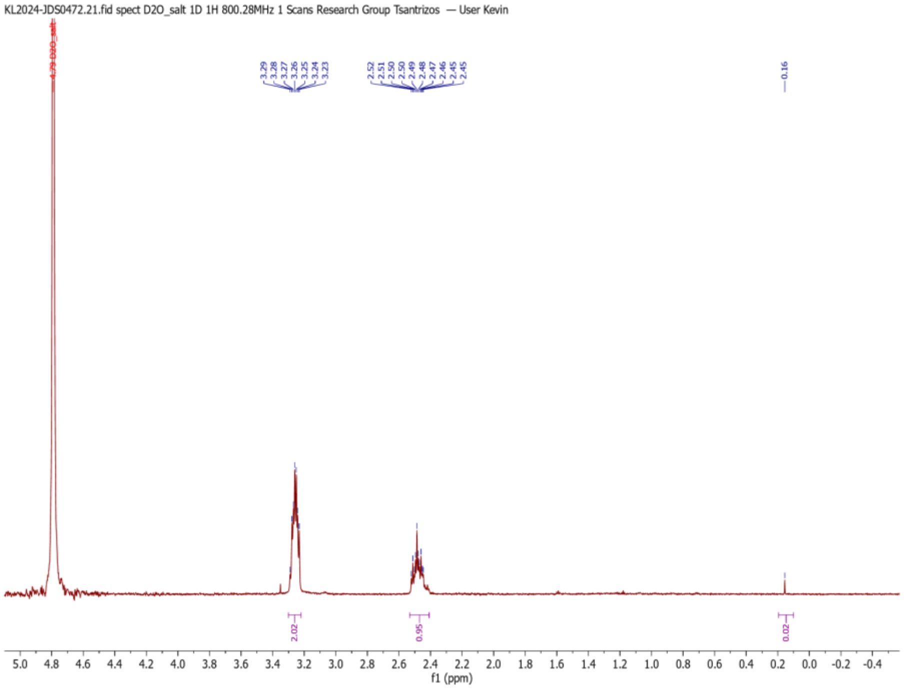

^{31}P NMR (203 MHz, 0.5% ND_4OD in D_2O) (**Compound 21**)

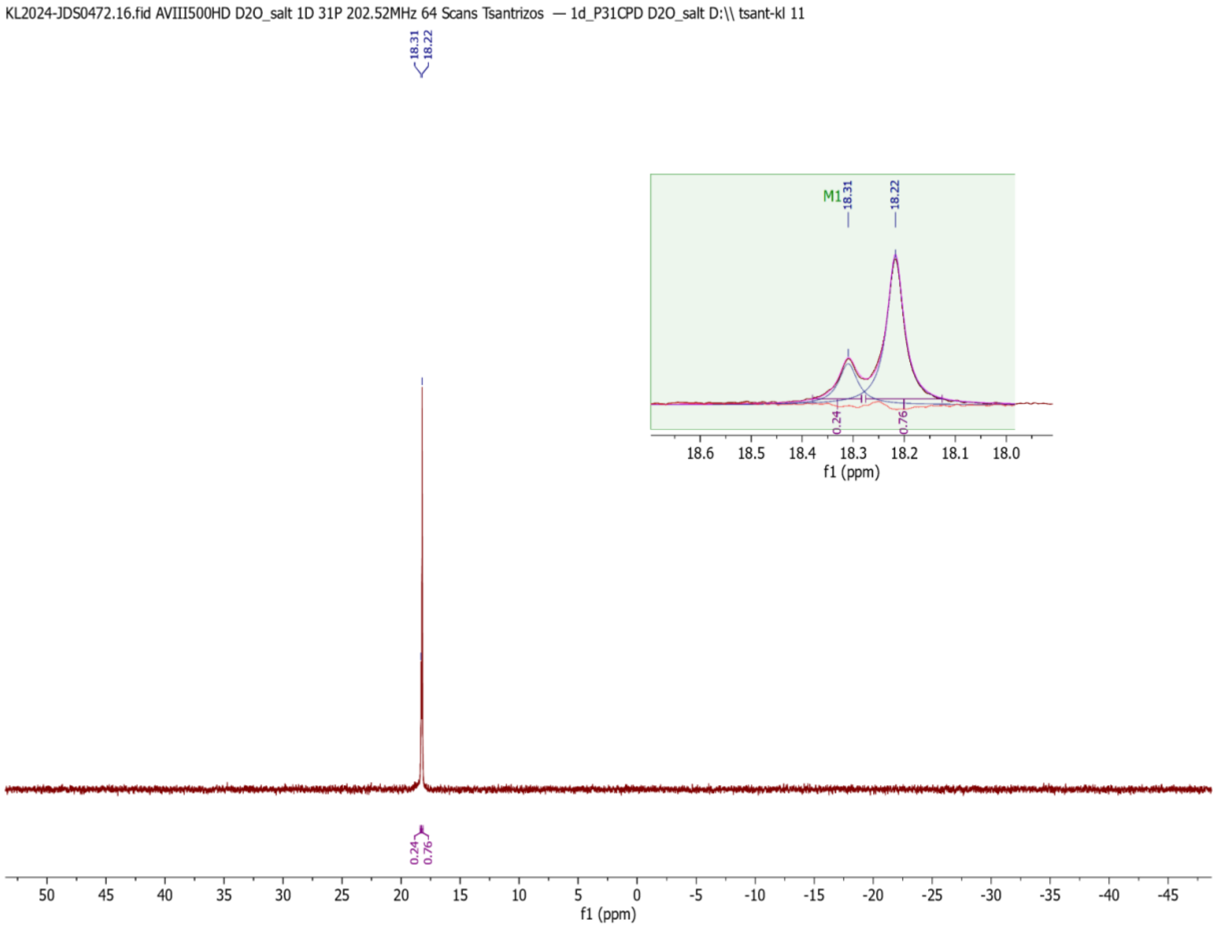

^{13}C NMR (201 MHz, 0.5% ND_4OD in D_2O) (**Compound 21**)

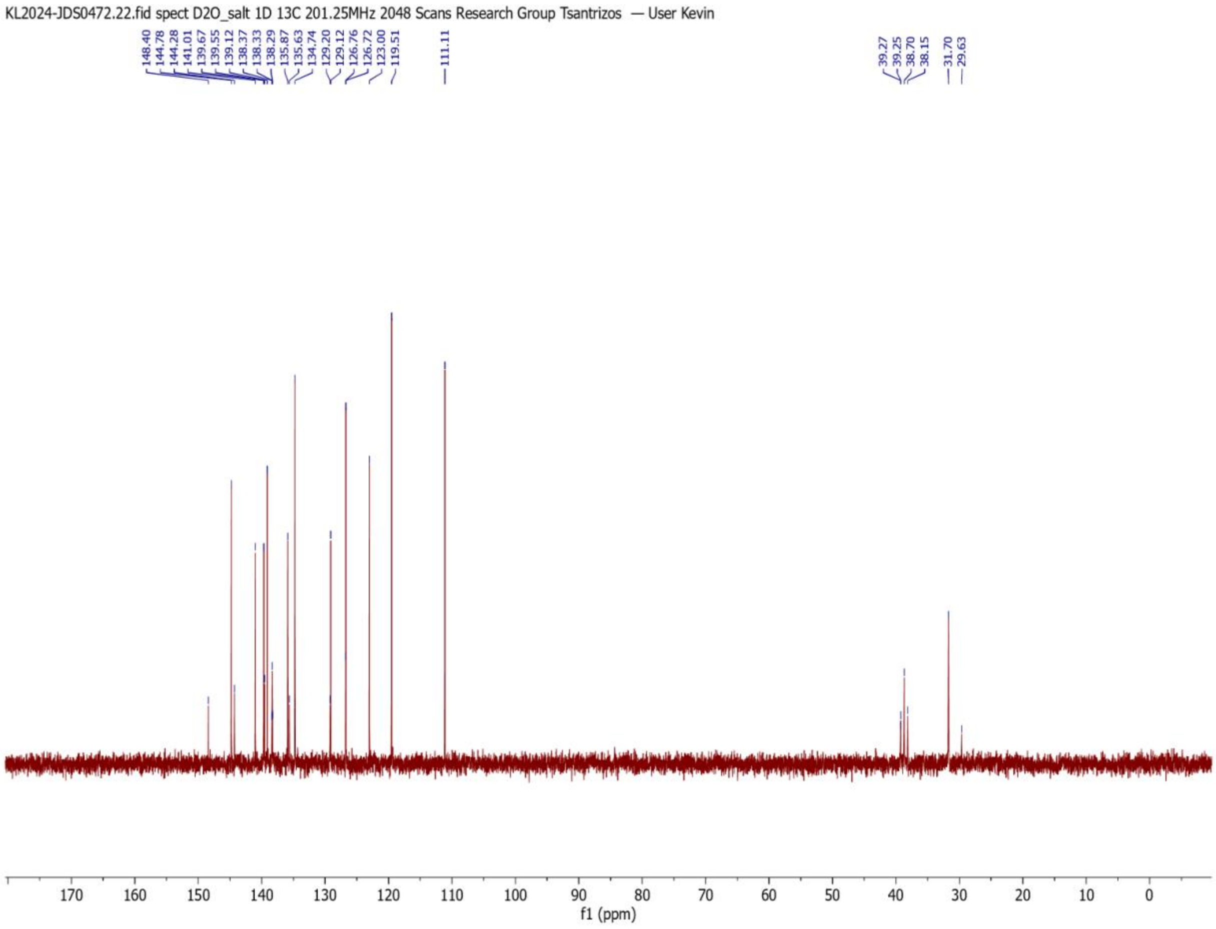

HPLC Chromatogram (**Compound 21**)

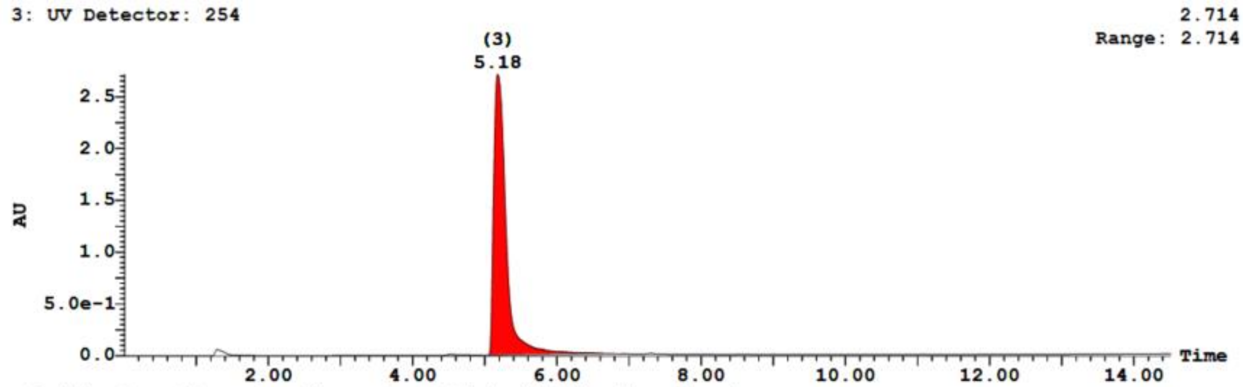

Peak Number	Time	AreaAbs	Area %Total	Height	Mass Found
3	5.18	538905	100.00	2705903	

LRMS (**Compound 21**)

Peak ID	Time	Target
3	5.18	No Target

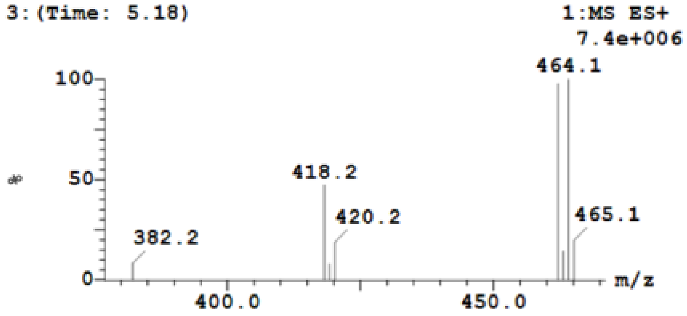

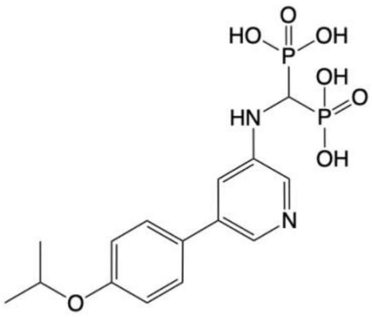

Compound 22

This compound has been previously reported (compound **9f** in reference 34). However, the spectra had not been given in the previous publication and are thus included here.

^{1}H NMR (800 MHz, 0.5% ND_4OD in D_2O) (**Compound 22**)

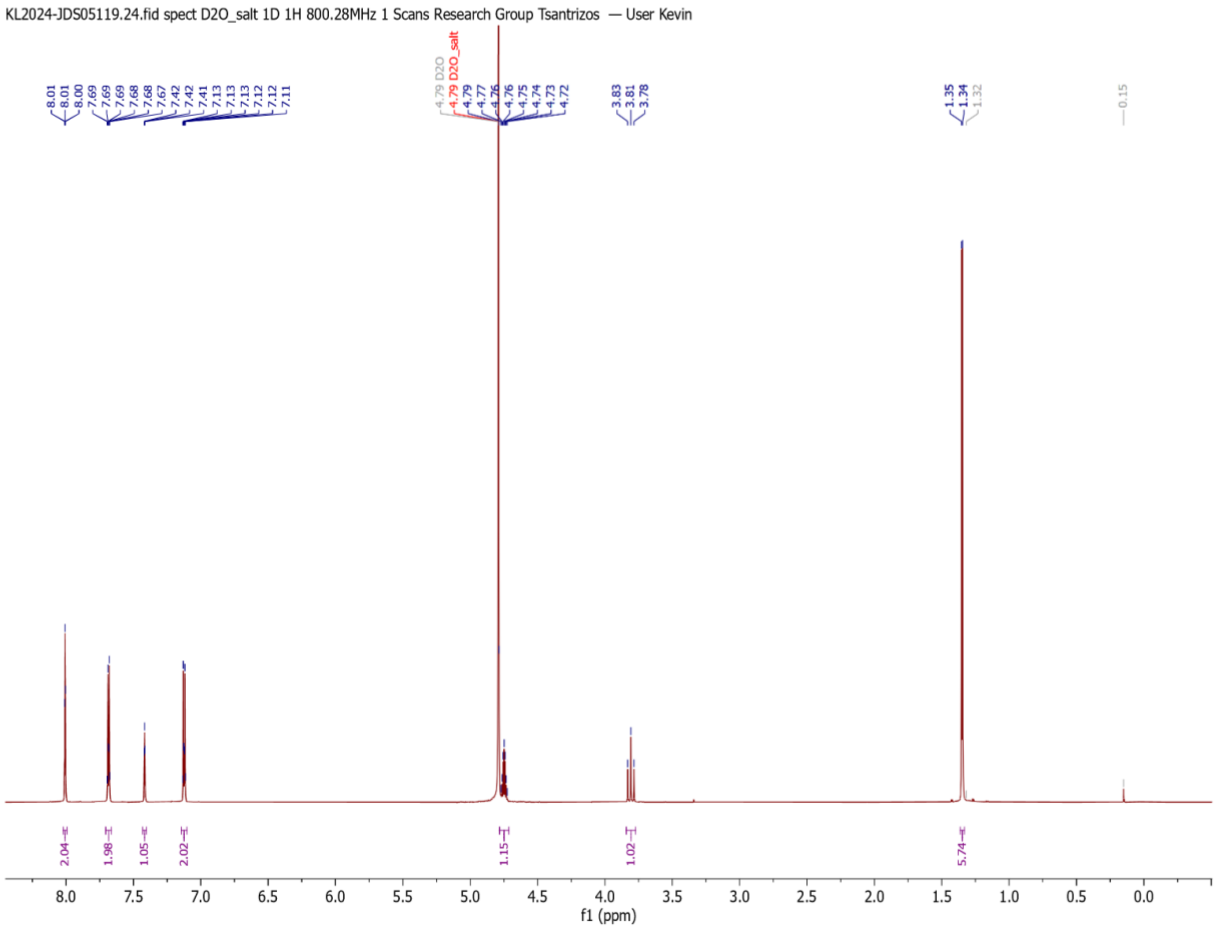

Expansion 1:

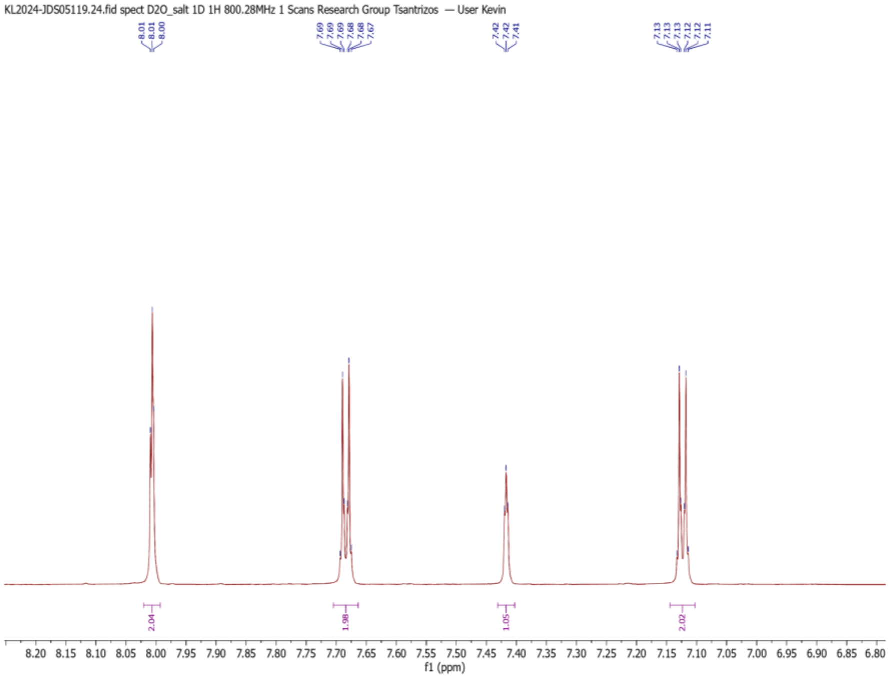

Expansion 2:

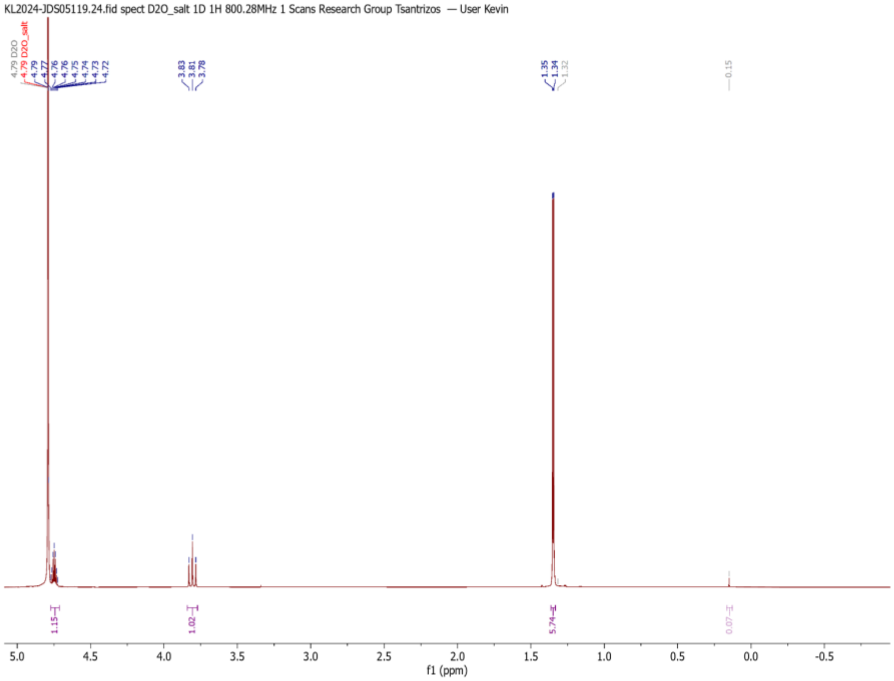

^{31}P NMR (203 MHz, 0.5% ND_4OD in D_2O) (**Compound 22**)

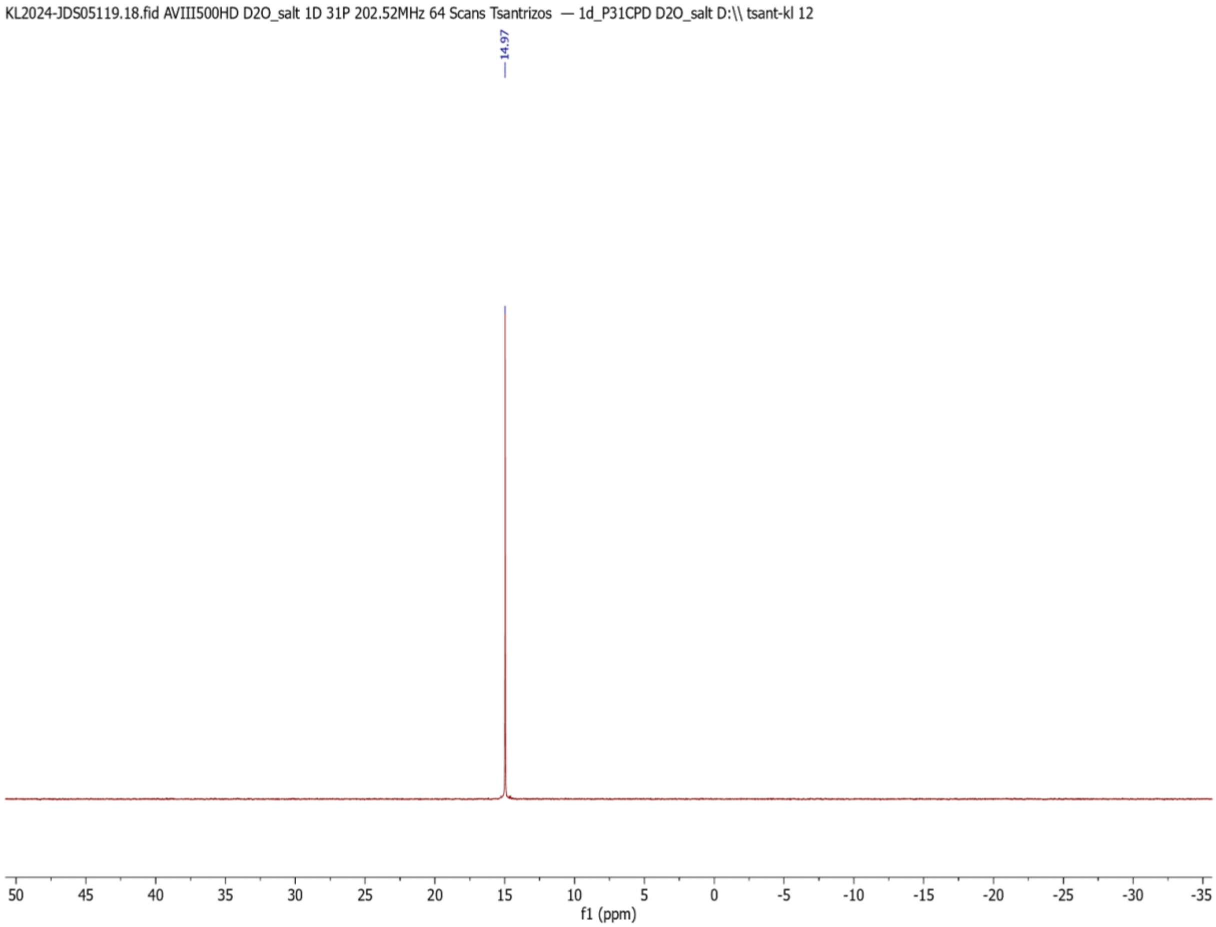

^{13}C NMR (201 MHz, 0.5% ND_4OD in D_2O) (**Compound 22**)

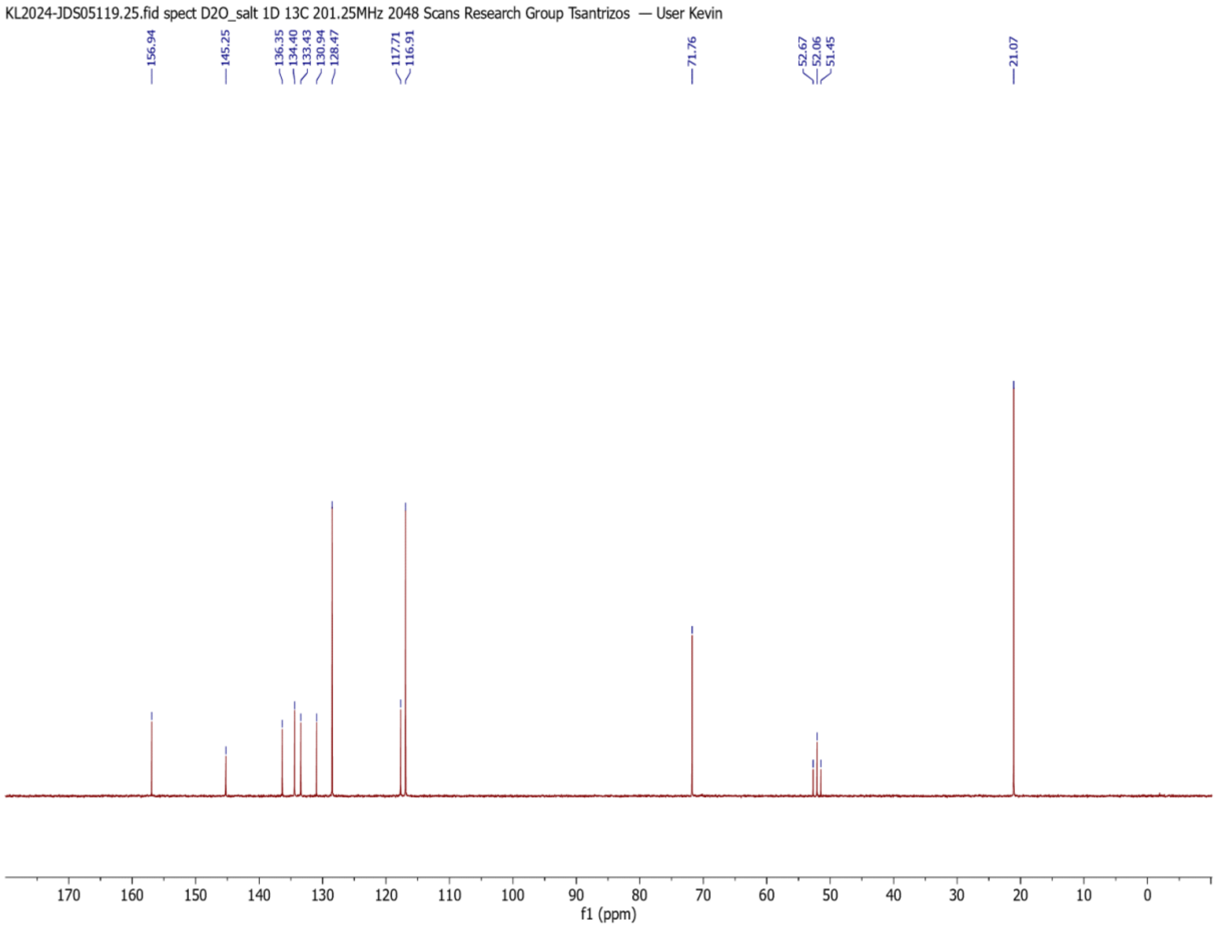

HPLC Chromatogram (**Compound 22**)

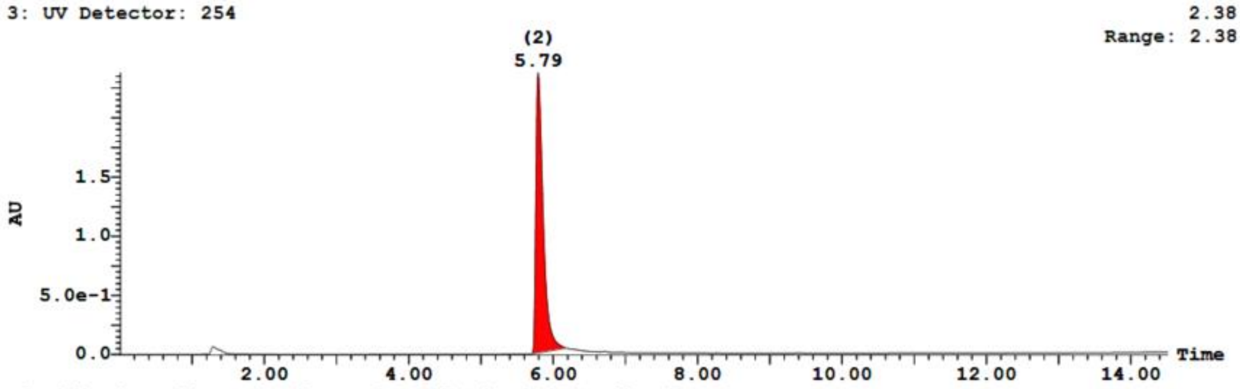

Peak Number	Time	AreaAbs	Area %Total	Height	Mass Found
2	5.79	278464	100.00	2365779	

LRMS (**Compound 22**)

Peak ID	Time	Target
2	5.79	No Target

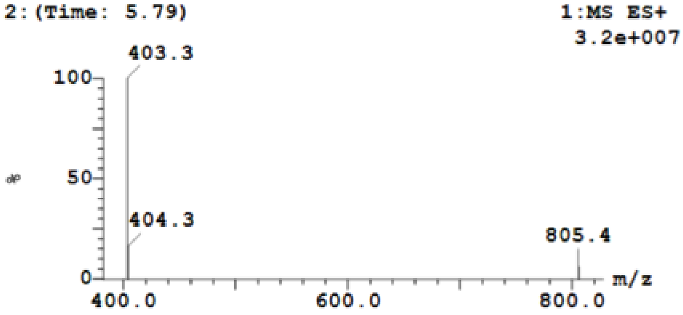

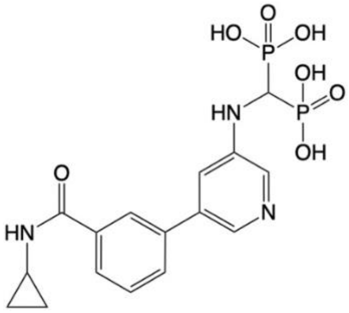

Compound 23

This compound has been previously reported (compound **9h** in reference 34). However, the spectra had not been given in the previous publication and are thus included here.

^{1}H NMR (800 MHz, 0.5% ND_4OD in D_2O) (**Compound 23**)

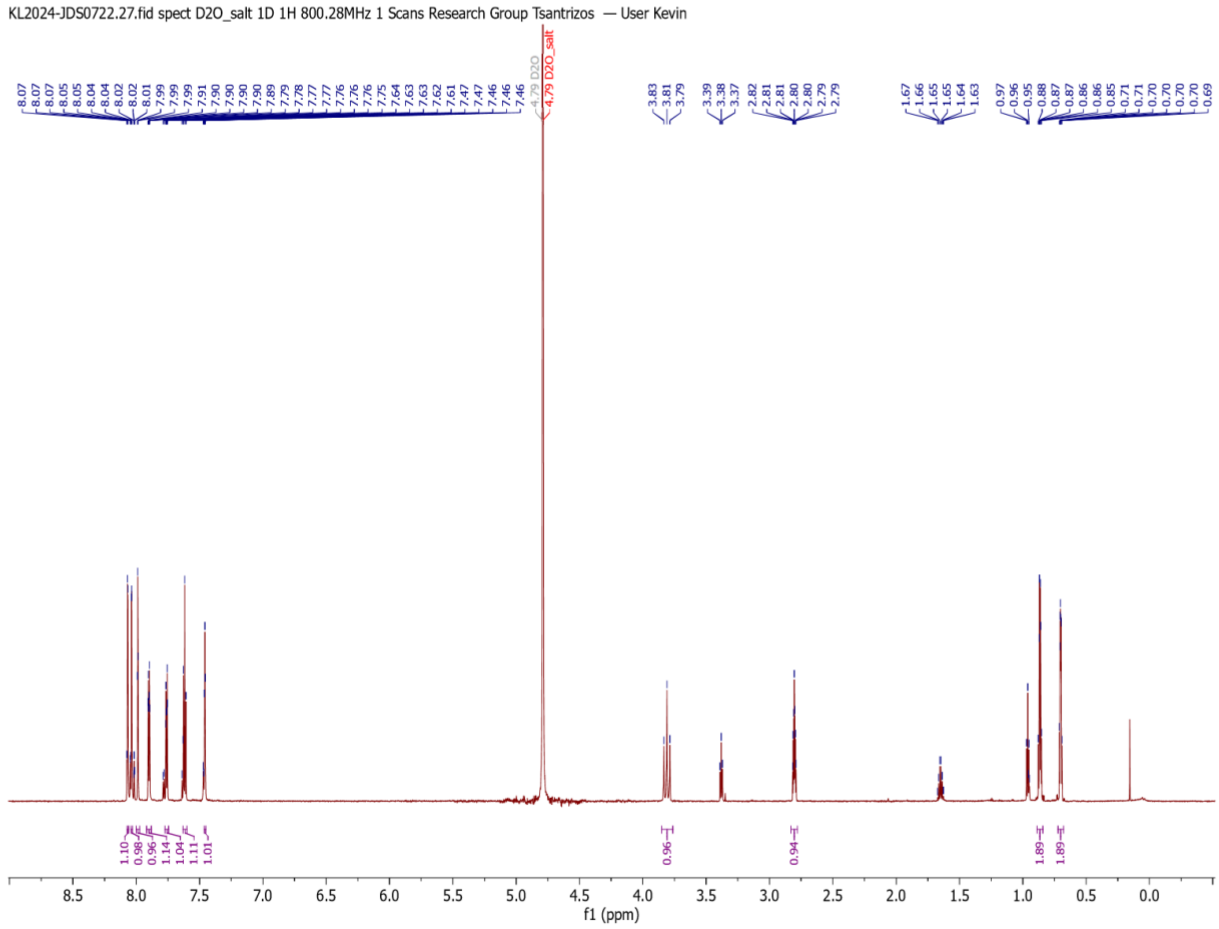

Expansion 1:

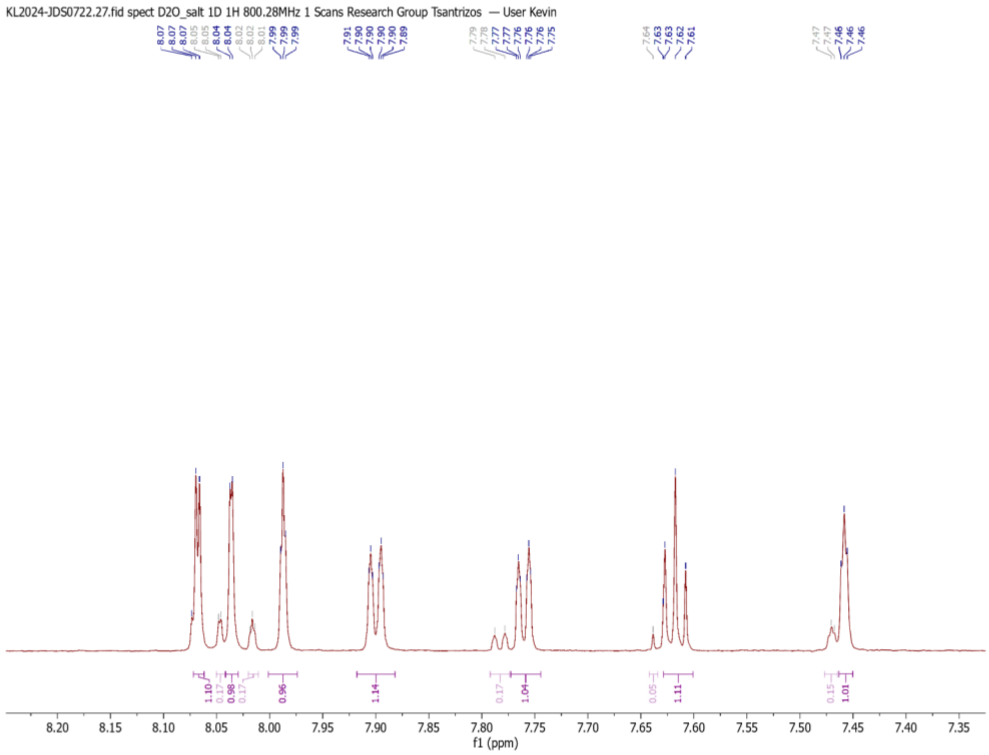

Expansion 2:

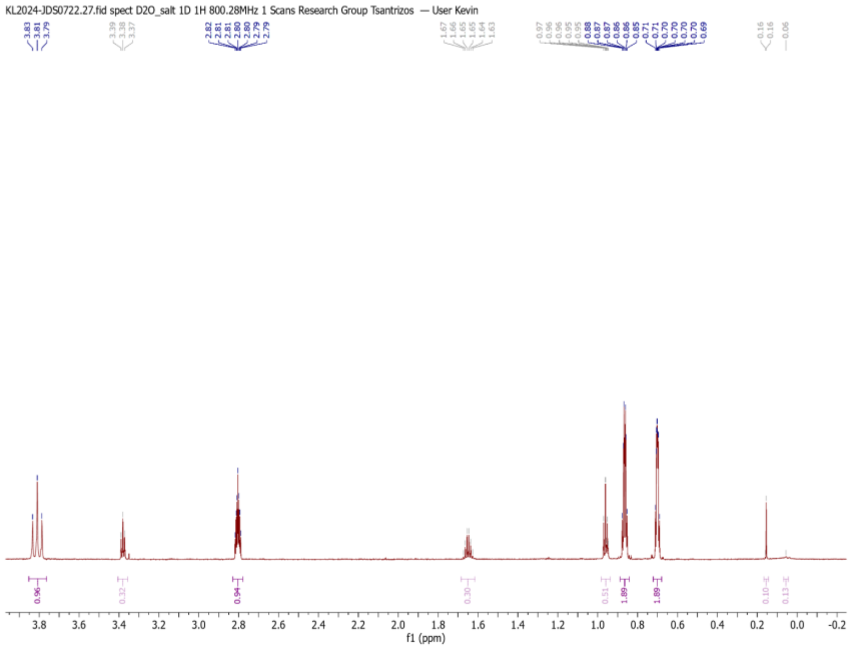

^{31}P NMR (203 MHz, 0.5% ND_4OD in D_2O) (**Compound 23**)

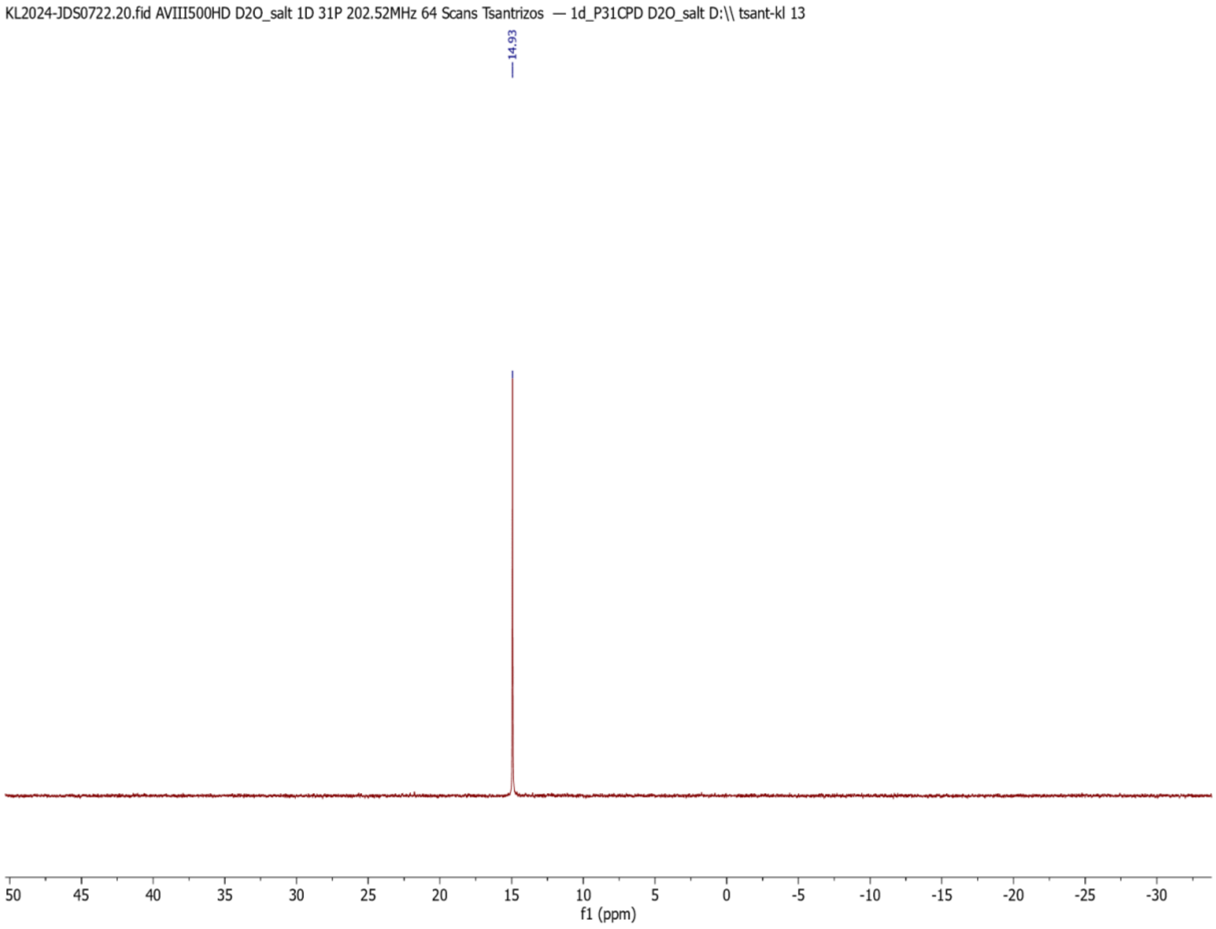

^{13}C NMR (201 MHz, 0.5% ND_4OD in D_2O) (**Compound 23**)

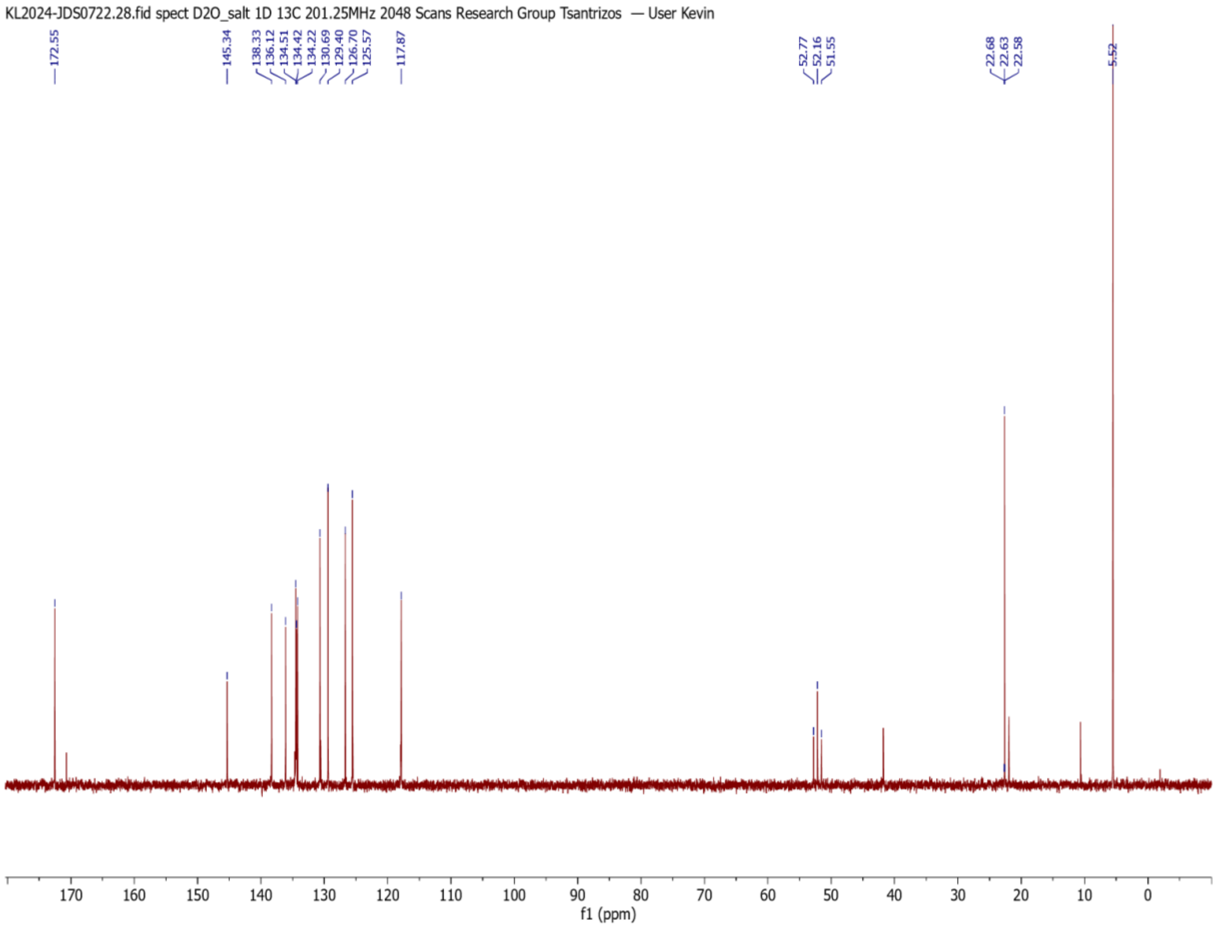

HPLC Chromatogram (**Compound 23**)

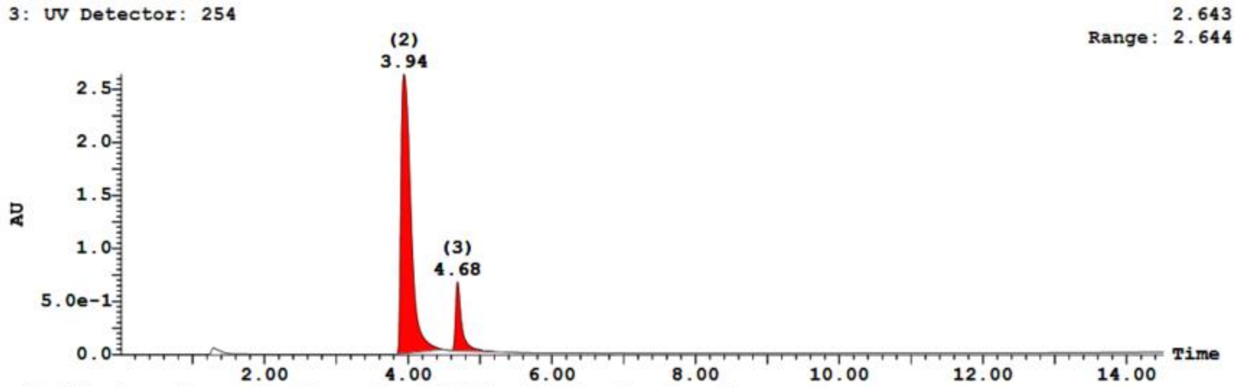

Peak Number	Time	AreaAbs	Area %Total	Height	Mass Found
2	3.94	419514	86.81	2633642	
3	4.68	63737	13.19	648986	

LRMS (**Compound 23**)

Peak ID	Time	Target
2	3.94	No Target

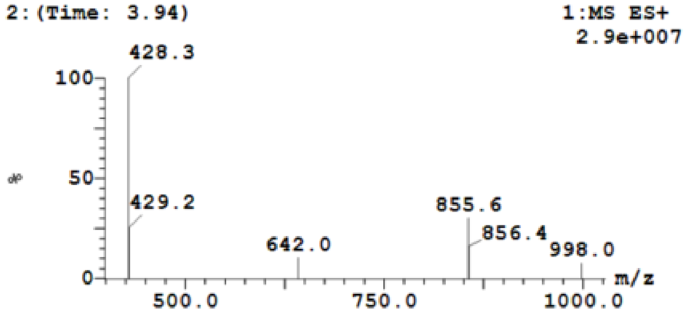

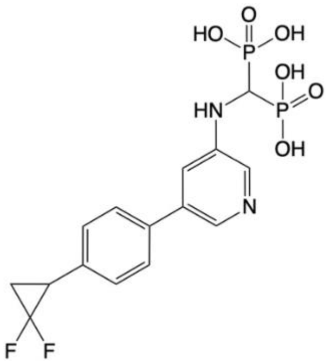

Compound 24

This compound has not been previously reported in the literature.

^{1}H NMR (800 MHz, 0.5% ND_4OD in D_2O) (**Compound 24**)

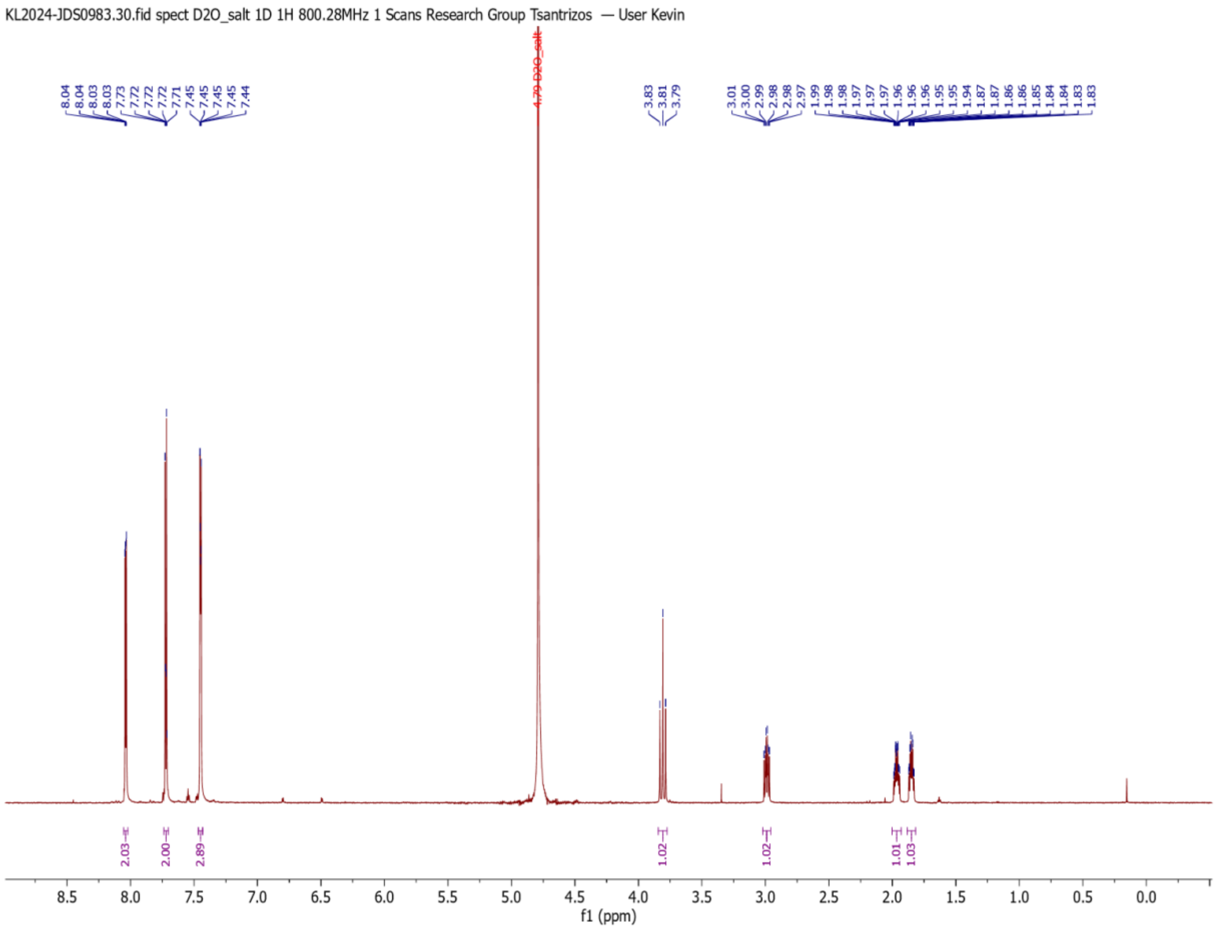

Expansion 1:

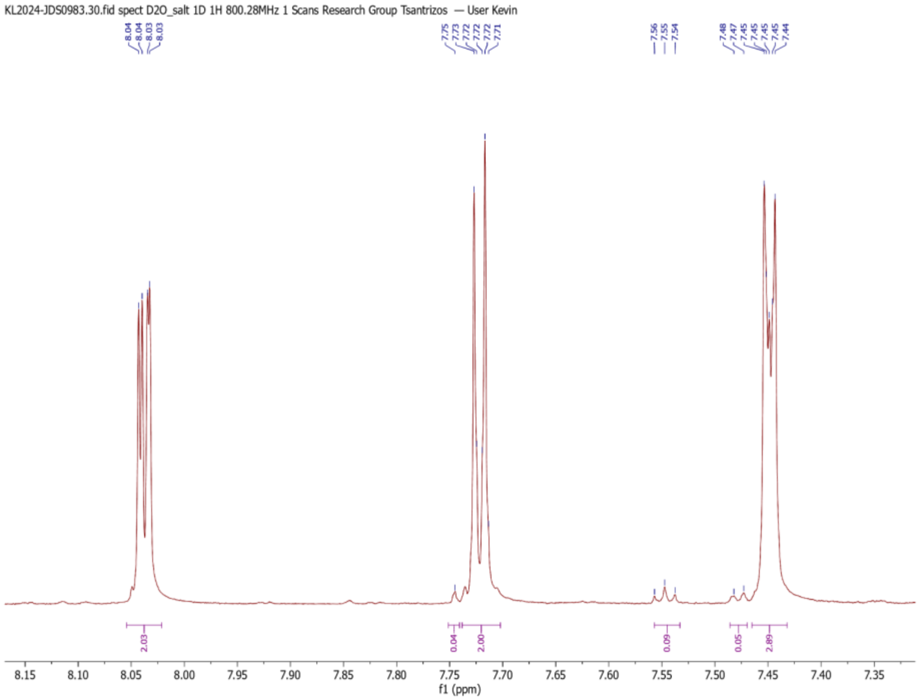

Expansion 2:

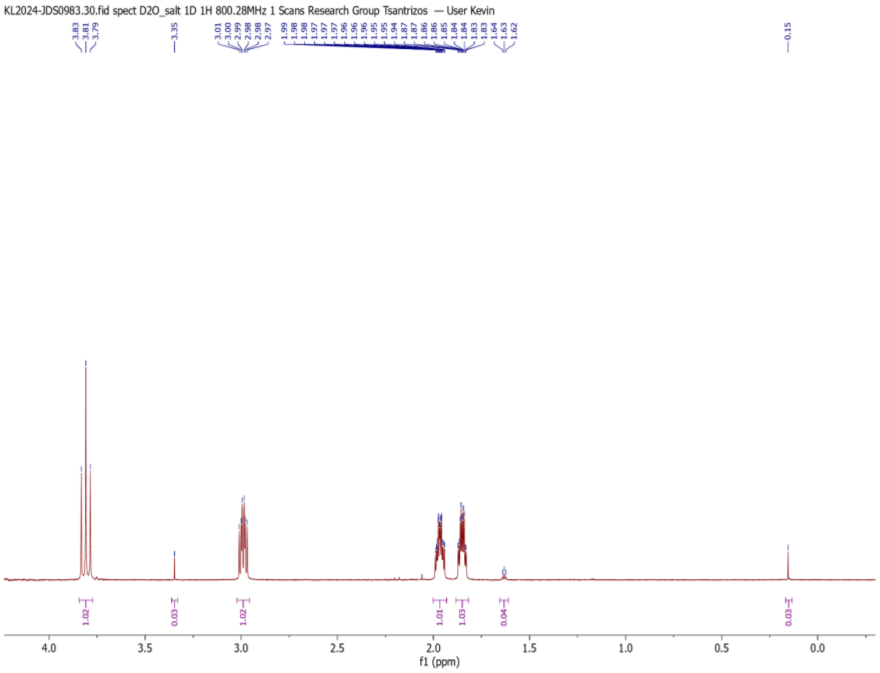

^{31}P NMR (203 MHz, 0.5% ND_4OD in D_2O) (**Compound 24**)

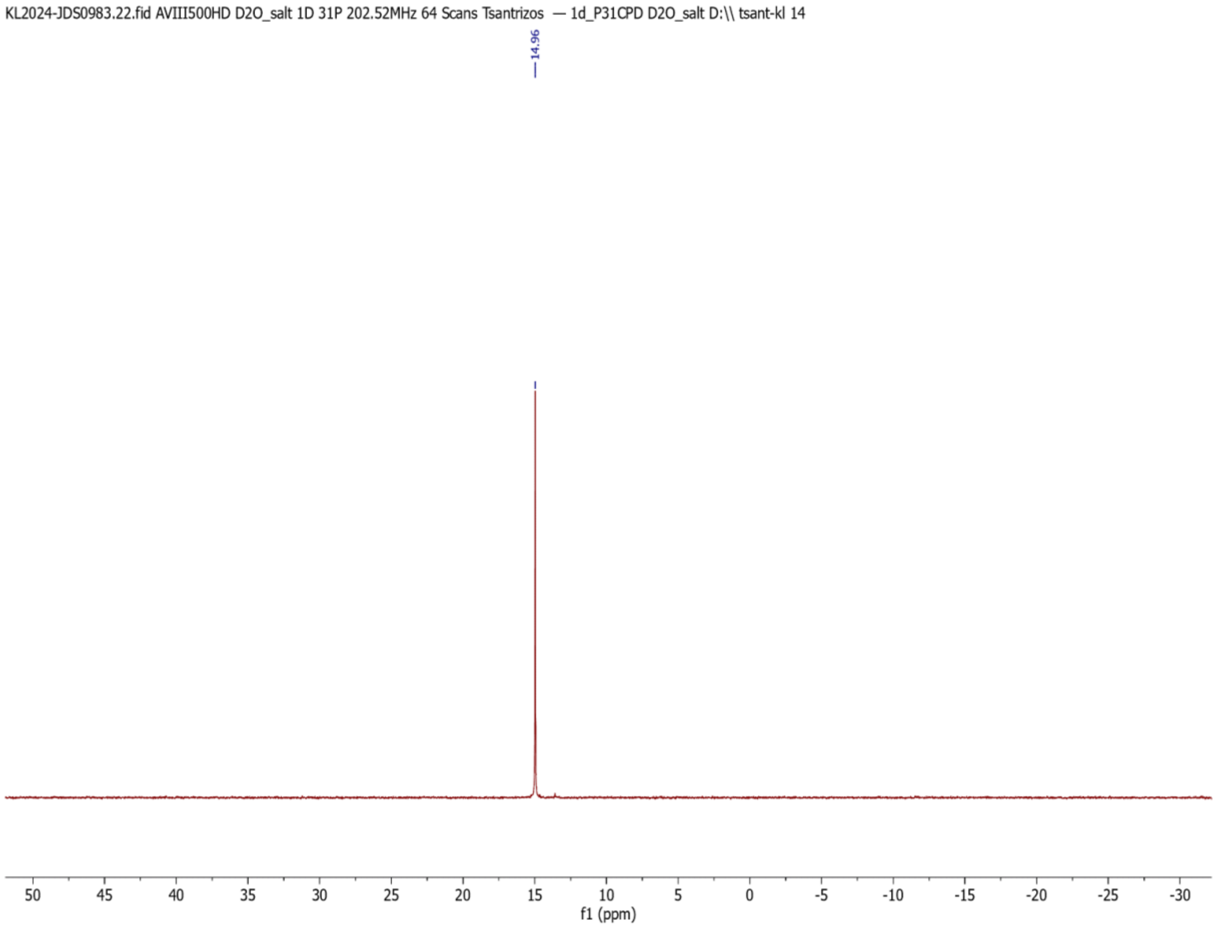

^{13}C NMR (201 MHz, 0.5% ND_4OD in D_2O) (**Compound 24**)

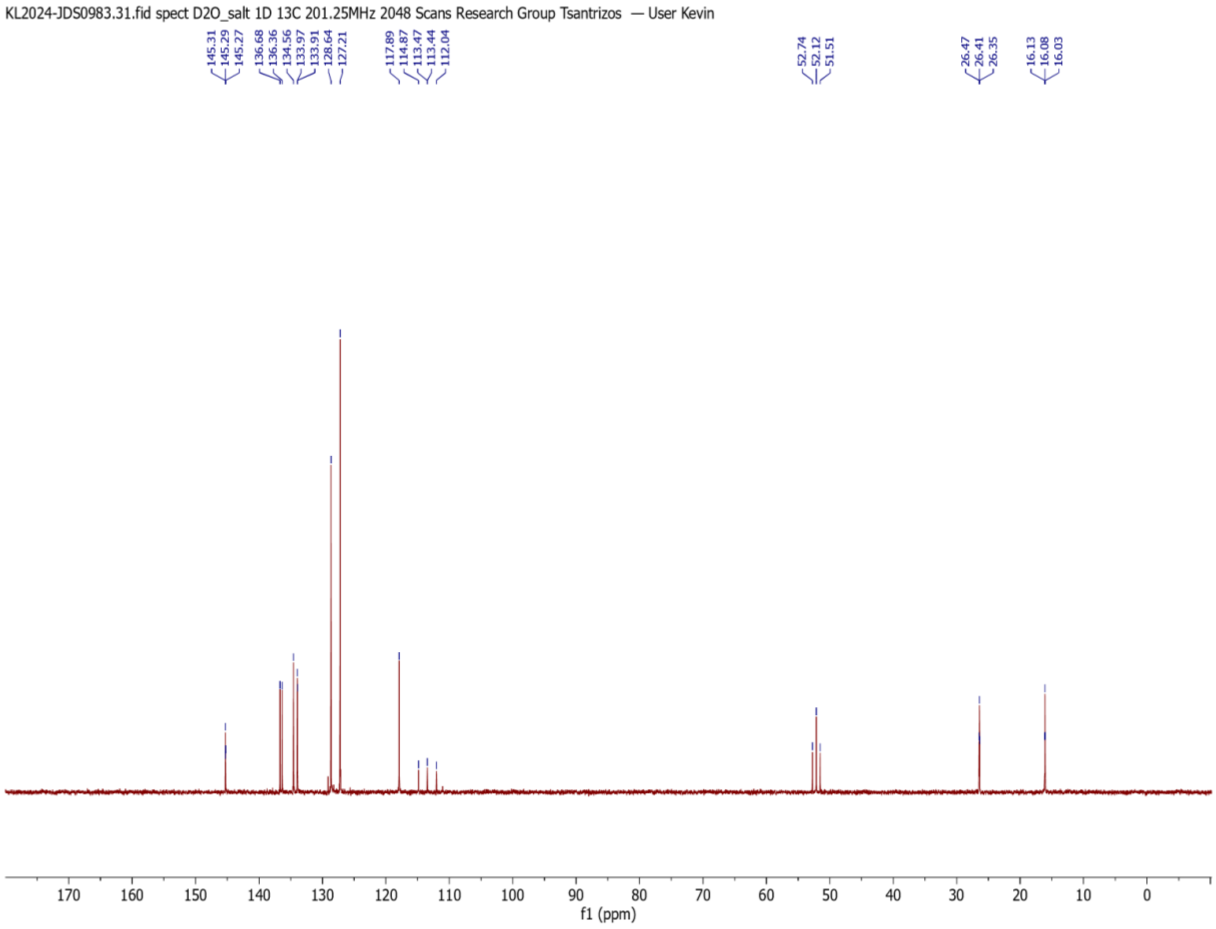

HPLC Chromatogram (**Compound 24**)

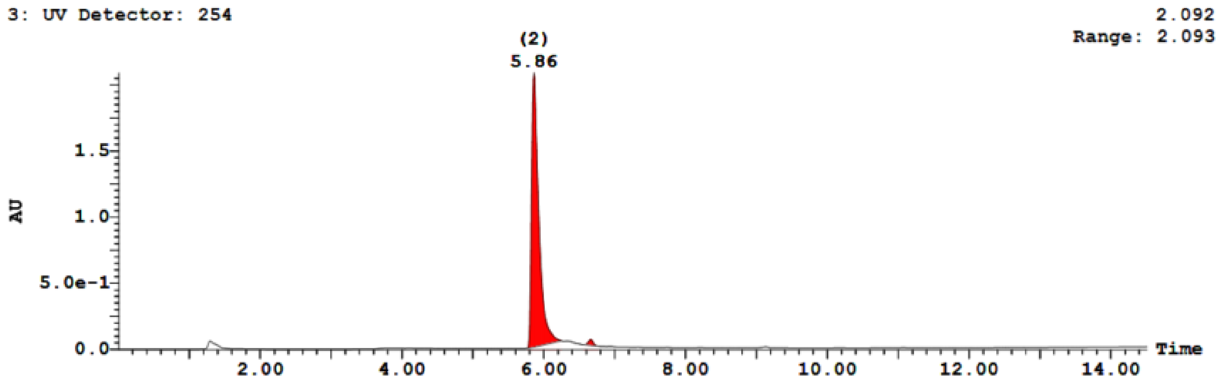

Peak Number	Time	AreaAbs	Area %Total	Height	Mass Found
2	5.86	246399	98.62	2074799	
4	6.66	3456	1.38	45457	

LRMS (**Compound 24**)

Peak ID	Time	Target
2	5.86	No Target

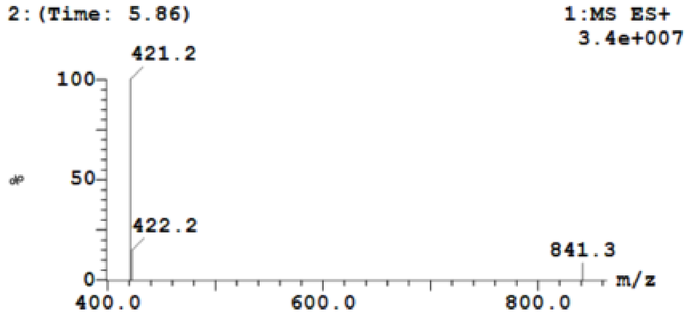

Supplement: Supplementary file 1 — Data S1. Compound characterization data. [file FEB4-14-1320-s001.pdf]
